# New Dimethoxyaryl-Sesquiterpene Derivatives with Cytotoxic Activity Against MCF-7 Breast Cancer Cells: From Synthesis to Topoisomerase I/II Inhibition and Cell Death Mechanism Studies

**DOI:** 10.3390/ijms26104539

**Published:** 2025-05-09

**Authors:** Ileana Araque, Rut Vergara, Jaime Mella, Pablo Aránguiz, Luis Espinoza-Catalán, Cristian O. Salas, Alejandro F. Barrero, José Quílez del Moral, Joan Villena, Mauricio A. Cuellar

**Affiliations:** 1 Facultad de Farmacia, Escuela de Química y Farmacia, Universidad de Valparaíso, Av. Gran Bretaña 1093, Valparaíso 2340000, Chile; ileana.araque@postgrado.uv.cl; 2 Departamento de Química, Universidad Técnica Federico Santa María, Avenida España 1680, Valparaíso 2340000, Chile; luis.espinozac@usm.cl; 3 Centro Interdisciplinario de Investigación Biomédica e Ingeniería para la Salud (MEDING), Escuela de Medicina, Facultad de Medicina, Universidad de Valparaíso, Viña del Mar 2520000, Chile; rut.vergara@postgrado.uv.cl; 4 Instituto de Química y Bioquímica, Facultad de Ciencias, Universidad de Valparaíso, Valparaíso 2340000, Chile; jaime.mella@uv.cl; 5 Centro de Investigación, Desarrollo e Innovación de Productos Bioactivos (CINBIO), Universidad de Valparaíso, Valparaíso 2340000, Chile; 6 Escuela de Ciencias de la Salud, Carrera de Química y Farmacia, Universidad Viña del Mar, Viña del Mar 2520000, Chile; pablo.aranguiz@uvm.cl; 7 Departamento de Química Orgánica, Facultad de Química y de Farmacia, Pontificia Universidad Católica de Chile, Avenida Vicuña Mackenna 4860, Macul, Santiago 702843, Chile; cosalas@uc.cl; 8 Departamento de Química Orgánica, Instituto de Biotecnología, Universidad de Granada, 18071 Granada, Spain; afbarre@ugr.es (A.F.B.); jfquilez@ugr.es (J.Q.d.M.)

**Keywords:** aryl-sesquiterpenes, cytotoxic activity, apoptosis, MCF-7 cells, topoisomerases I/II, in silico studies

## Abstract

Breast cancer is a prevalent type of cancer worldwide, leading to both high incidence and mortality, and hence, effective and safe drugs are needed. Because of this, the use of natural products and their derivatives has become a popular strategy for developing new chemotherapeutic agents. In this study, 17 new sesquiterpene-aryl derivatives were synthesized using (−)-drimenol as the starting material. The cytotoxicity of these semi-synthetic derivatives was determined in MCF-7 cells, a breast cancer model, and in a non-tumor cell line, MCF-10, to evaluate selectivity. The results show that five of these sesquiterpene derivatives had IC_50_ values between 9.0 and 25 µM. Of these, compound **14c** stands out for its higher cytotoxicity in MCF-7 cells but lower cytotoxicity in MCF-10 cells, being more selective than daunorubicin (selective index values of 44 and 28, respectively). In addition, compound **14c** induced oxidative stress in MCF-7 cells, activated caspases-3/7, and selectively inhibited topoisomerase II (TOP2) versus topoisomerase I (TOP1) in MCF-7 cells. In silico studies allowed us to propose a binding mode for **14c** to the TOP2 DNA complex to validate the experimental results. Therefore, this study demonstrated the importance of aryl-sesquiterpene structures and their promising profiles in the search for new bioinspired antitumor drugs in natural products.

## 1. Introduction

Cancer, as the second leading cause of death globally, poses not only a significant challenge to public health, but also a significant social and economic issue in this century. It is believed to account for approximately 16.8% of all noncommunicable disease-related deaths and 22.8% of such deaths globally [1]. Demographic projections suggest that the number of new cancer cases will reach 35 million by 2050, as estimated by GLOBOCAN 2022. Among these cases, breast cancer (BC) has emerged as a significant concern for women in recent years. BC is now the most frequently diagnosed cancer worldwide and the primary cause of cancer-related deaths among patients [2,3].

Throughout medical history, natural products have proven to be valuable sources of novel anticancer agents. Natural products, including various types, can affect several cellular pathways such as apoptotic cell death, proliferation, migration/invasion, angiogenesis, and metastasis. However, current breast cancer drugs often face limitations such as drug resistance, toxicity, and lack of specificity, which underscores the need for new therapeutic alternatives. By triggering intracellular signals that lead to the death of cancer cells, these products can be highly beneficial owing to their chemical diversity, low toxicity, safety, and accessibility [4,5]. Nevertheless, there remains a need for more effective antineoplastic agents. Apoptosis is an intriguing process, and it has been suggested as a promising target for cancer treatment.

**Figure 1:**
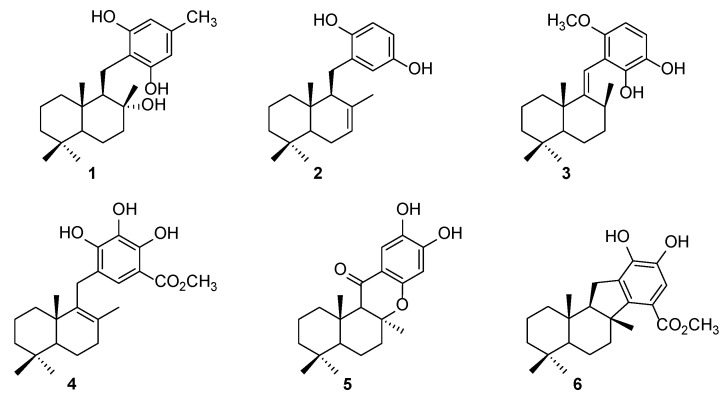
Molecular chemical structures of natural bioactive merosesquiterpenoids **1**–**6**.

There exists a great variety of natural metabolites of marine origin, isolated from sponges and/or marine algae, which include in their structure a drimanic skeleton together with an aromatic fragment, possessing different substitution oxygenated patterns. Merosesquiterpenoids are a class of compounds that have garnered considerable interest due to their diverse range of biological activities, including antimicrobial, antiviral, immunomodulatory, and cytotoxic properties [6,7]. For example, albaconol (**1**, Figure 1), isolated from the basidiomycete *Albatrellus confluens* [8], inhibits the proliferation of leukemia (K562), lung adenocarcinoma (A549), gastric adenocarcinoma (BGC-823), and breast carcinoma (Bcap-37) cancer cells [5]. Albaconol (**1**) specifically targets the catalytic activity of topoisomerase II (TOP2) and functions as a dose-dependent inhibitor of this enzyme by increasing the TOP2-mediated DNA cleavage complex and inhibiting the religation activity of TOP2 [9,10]. In contrast, the marine natural product *ent*-isozonarol (**2**, Figure 1), obtained from the marine sponge Dysidea, has also shown antitumor activity against the tumor lines lymphocytic leukemia (P-388), HT-29, melanoma (MEL-28), lung carcinoma (A-549), and mammary adenocarcinoma (MDA-MB-231) [11,12]. Another example of a merosesquiterpenoid is wiedendiol B (**3**, Figure 1), isolated from the marine sponge *Xestospongia wiedenmayeri*; it impedes cholesterol ester transfer protein (CETP) and thereby promotes the survival of BC cells when confronted with cholesterol-depleting agents [13,14,15]. Similarly, the metabolite dactylospontriol (**4**, Figure 1), also isolated from *Albatrellus confluens*, has shown moderate cytotoxic potential in three colorectal cancer cell models (HCT116, RKO, and HT29 cell lines) [16,17], whereas 15-oxopuupehenol (**5**, Figure 1) from *Hyrtios* species has antimalarial and antitumor activity against several human cancer lines, such as the leukemia cell line (P-338), adenocarcinoma human alveolar basal epithelial cell line (A-549), and colon adenocarcinoma cell line (HT-29), and it has been reported as a TOP2 inhibitor [18,19]. Finally, the metabolite pelorol (**6**, Figure 1), from the marine sponge *Dactylospongia elegans*, induces apoptosis in melanoma cancer cells (501 Mel). Furthermore, it has been discovered that compound **6** serves as an in vitro activator of the inositol-5-phosphatase (SHIP) enzyme, which is known to play a role in suppressing tumor formation during the development of leukemia and lymphoma [20,21].

**Figure 2:**
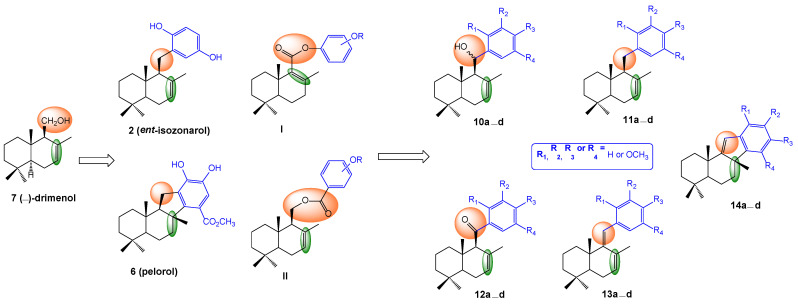
The development of a design strategy for creating new sesquiterpene derivatives inspired by natural products against MCF7 cells.

The focus of medicinal chemistry research has been on developing new analogs through more efficient synthetic methods that involve structural variations of compounds that have already been discovered in nature. All of the bioactive sesquiterpene derivatives depicted in Figure 1 serve as chemical building blocks for the creation of innovative anticancer agents. In this respect, we have previously reported ester-aryl derivatives (**I**–**II**) with promising cytotoxic activity and topoisomerase I (TOP1) inhibition from (−)-drimenol (**7**, Figure 2) [22].

Therefore, in this work, we designed a new series of sesquiterpene derivatives obtained from **7** with the aim of deepening our understanding of the structural requirements for obtaining compounds with anti-breast cancer properties. The design of the proposed compounds is shown in Figure 2 and is based on modifications to the sesquiterpene and aryl scaffolds, including (i) the substitution of the methylene C-11 linker with alternative chemical groups (orange fragment in Figure 2); (ii) the preservation of the double bond position in the drimane skeleton (green fragment in Figure 2); and (iii) the introduction of certain substitutions in the dimethoxy pattern in the arene ring (the blue fragment in Figure 2). Subsequently, the biological effects of all synthesized compounds were evaluated for their biological effect on the MCF7 breast cancer cell line and MCF-10 non-neoplastic cell control. In addition, their ability to inhibit human TOP1 and TOP2, ROS generation and apoptosis induction by caspases-3/7 activity were performed to determine a possible mechanism of action of these sesquiterpene derivatives. Molecular docking studies were performed on TOP2 to understand a possible binding mode for the most promising compound, and the results were later confirmed by molecular dynamic studies.

## 2. Results and Discussion

### 2.1. Chemistry

**Scheme 1:**
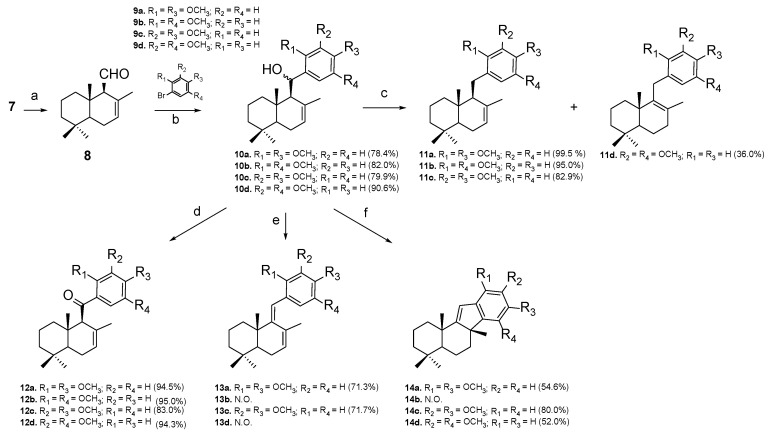
Synthesis of sesquiterpene-aryl ester derivatives. Reagents and conditions: (a) PCC, CH_2_Cl_2_, 0 °C, 1 h; (b) *tert*-BuLi, **9a**–**d**, THF, **8**; (c) Et_3_SiH, TFA, DCM, Ar, 0 °C to 25 °C; (d) PCC, CH_2_Cl_2_, (e) I_2_, DMSO, toluene, reflux; (f) I_2_, toluene, reflux. N.O. = not obtained.

New dimethoxyaryl-sesquiterpene derivatives **10a**–**d**, **11a**–**d**, **12a**–**d**, **13a** and **13c**, and **14a**, **c**–**d** were obtained by the short and efficient synthetic route shown in Scheme 1. (-)-drimenol (**7**) was the initial material and was sourced from the hexane extract of the bark of *Drimys winteri* Forst [23]. Subsequently, **7** was treated with pyridinium chlorochromate (PCC) in dichloromethane at 0 °C to obtain a good yield of drimenal **8** (78%) [24]. Nucleophilic addition of organolithium derivatives **9a**–**d** to aldehyde **8**, according to known procedures [25,26,27,28], gives the corresponding secondary alcohols **10a**–**d** in very good yields (78.4–90.6%).

**Scheme 2:**
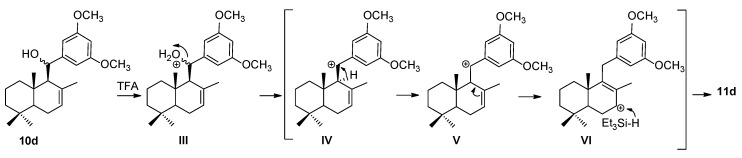
Mechanism proposed to obtain reduced product, **11d**.

With the aim of obtaining new sesquiterpene derivatives of **10a**–**d** with different structural patterns (Figure 2), these compounds were subjected to different reaction conditions, as shown in Scheme 1 **b**–**e**. Firstly, **10a**–**d** were reduced with triethylsilane (Et_3_SiH) in trifluoroacetic acid (TFA) under an inert atmosphere to obtain reduced compounds **11a**–**c** in good to excellent yields (82.9–99.5%), following the methodology described by Barclay with some modifications [29]. However, when **10d** was subjected to the same reaction conditions, compound **11d** was obtained in a moderate yield (36%). An explanation of the differences in reactivity between **10a**–**c** and **10d** is based on the positions of the methoxy groups in the aromatic fragment since, according to the mechanism proposed in Scheme 2, benzylic carbocation (**IV**) cannot be stabilized by the methoxy groups of the ring because they are in the meta-positions. Instead of this possibility, a rearrangement of **IV** allows for the formation of a more stable carbocation **V**, which by resonance gives rise to **VI** and finally to **11d**.

**Scheme 3:**
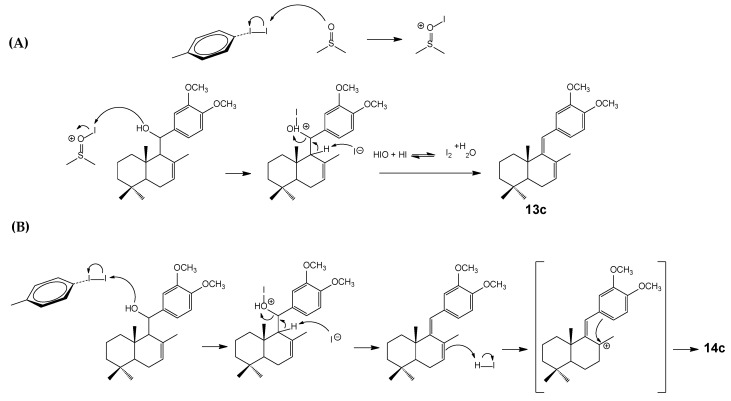
Proposed mechanisms to obtain (**A**) diene **13c**; (**B**) cyclized compound **14c**.

In a second part, the oxidation products of **12a**–**d** were obtained by the oxidation of **10a**–**d** with pyridinium chlorochromate (PCC) in dichloromethane in an efficient step (83–95% yields). Thirdly, dienes **13a** and **13c** and cyclized compounds **14a**, **c**–**d** (Scheme 1) were prepared using a similar methodology described by Domingo et al. [30,31] (Scheme 2). Dienes **13a** and **13c** (71.3–71.7%) were obtained using iodine and dimethylsulfoxide (DMSO) in reflux using the mechanism shown in Scheme 3A [30]. On the other hand, the use of iodine and toluene in reflux favors an acidic medium in which the cyclized products **14a**, **14c**, and **14d** are obtained in good yields (52–80%) using the mechanism proposed in Scheme 3B [32].

**Figure 3:**
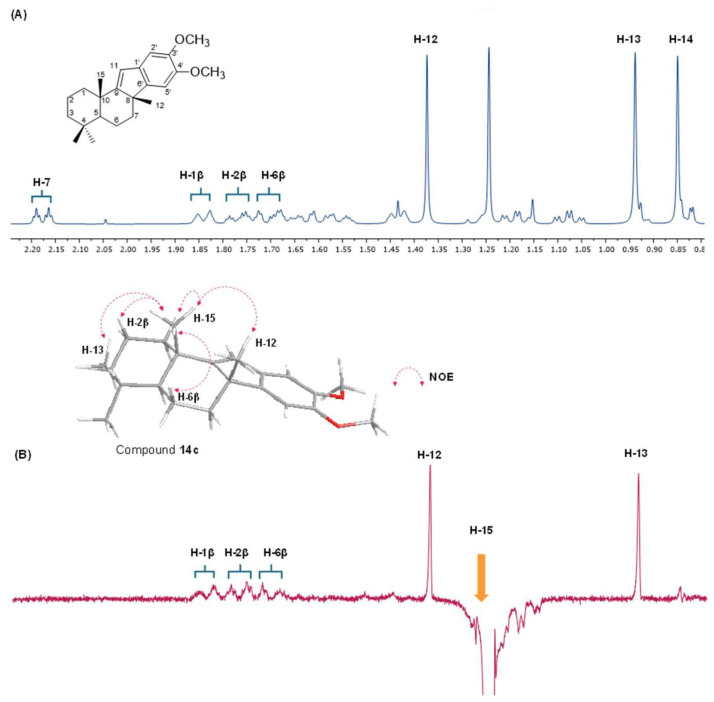
(**A**) ^1^H NMR (CDCl_3_) spectrum of compound **14c**. (**B**) Selective ^1^D NOE spectrum, showing the selective excitation of CH_3_-15 and NOE effects observed with CH_3_-12 and CH_3_-13.

The unambiguous structural identification of compound **14c** was conclusively established through the application of ^1^H and ^13^C NMR spectroscopic techniques, as demonstrated in the Supplementary Material. Additionally, these signals were correlated with 2D HSQC and 2D HMBC, and the spatial orientation of CH_3_-12 was determined using a selective 1D NOESY experiment, where the signal of CH_3_-15 (δ_H_ = 1.24 ppm) showed spatial correlations with CH_3_-13 and CH_3_-12, indicating that these H atoms have the same spatial β-orientation (Figure 3).

### 2.2. Biological Evaluation

In this study, we wondered whether semi-synthetic compounds have potential antitumoral properties against BC. Therefore, we were interested in investigating the effect of the compounds on the MCF-7 cell line. Firstly, we studied the effect on cell viability using a colorimetric MTT assay, which was performed to quantify the cytotoxic activity; each compound was evaluated at different concentrations (500−100−20−4−0.8−0.16−0.032−0 µM). Daunorubicin was also used as a positive control for comparison with a reference drug. To demonstrate selectivity towards MCF-7 cells, the non-tumoral cell line MCF-10 was used as a control by calculating the selectivity index (SI). Although the MCF-10 cell line provides a useful selectivity model, it may not fully represent the varying sensitivities of different normal cell types within a multicellular organism. The SI value indicates the ratio of toxicity in mammalian host cells to that in a tumoral cell. The data obtained in response to different concentrations of compounds were used to calculate the IC_50_ and corresponding SI values, as shown in Table 1.

Table 1 displays the IC_50_ values for all compounds analyzed. From a chemical point of view, the analysis of the cytotoxicity and SI data showed that in each subset of compounds, those presenting a 3,4-dimethoxy chemical pattern in the aromatic ring were **10c**, **11c**, **12c**, **13c**, and **14c**. Independent of the linker between the sesquiterpene fragment and the aryl moiety, there was a higher cytotoxic effect on MCF-7 cells, as well as higher selectivity compared to the normal cell line (IC_50_ and SI values between 9.0 and 30.1 μM and between 4.3 and 44, respectively). An exception was compound **14a** (with a 2,4-dimethoxy pattern), which has a cytotoxic effect on MCF-7 cells like that of **14c** (IC_50_ = 8.4 and 9.0 μM) but with a lower SI value, which was compared to **13c** (6.3 versus 6.0). Therefore, a slight structure–activity relationship between these compounds can be proposed. The compounds with the highest potency in terms of cytotoxicity follow the order of potency: 3,4-dimethoxy > 2,4-dimethoxy > 2,5-dimethoxy or 3,5-dimethoxy. In terms of selectivity, the presence of a new cycle in the system leads to a significant increase in these values in the final compounds, with the most active and selective compound being **14c**. The rigidity of the structures together with the 3,5-dimethoxy substitutions are key to the potency and selectivity for the MCF-7 tumor cell line.

While none of these sesquiterpene derivatives were more potent than daunorubicin on MCF-7 cells (IC_50_ = 0.33 μM), **14c** stood out because it was much less toxic than this drug in MCF-10 cells (IC_50_ = 393 versus 9.32 μM) and therefore much more selective than daunorubicin (SI values 44 versus 28). This result is important because a required property of an anticancer drug is its ability to selectivity kill cancer cells, and not just the ability to kill cancer cells at low concentrations. These results are consistent with the National Cancer Institute (NCI)’s protocols, where compounds with IC_50_ values < 10 μM or 15 μM are considered active [34]. Interestingly, the base sesquiterpene system of these derivatives, (-)-drimenol, was inactive, as previously reported by our group [34]. Therefore, it was shown that the incorporation of the aromatic ring in the targeted products increased cytotoxicity in most cases. Furthermore, in a previous investigation, we evaluated aryl-sesquiterpene ester-derived compounds that showed lower activity and selectivity compared to the studied compounds [22].

Consequently, **14c** emerged as the most compelling substance in this study, exhibiting strong potency and a substantial selectivity index profile. However, **10c**, **11c**, **12c**, **13c**, and **14c** were selected for further investigation. The respective IC_50_ values of each compound were used to perform the following experiments.

### 2.3. Intracellular ROS Generation

**Figure 4:**
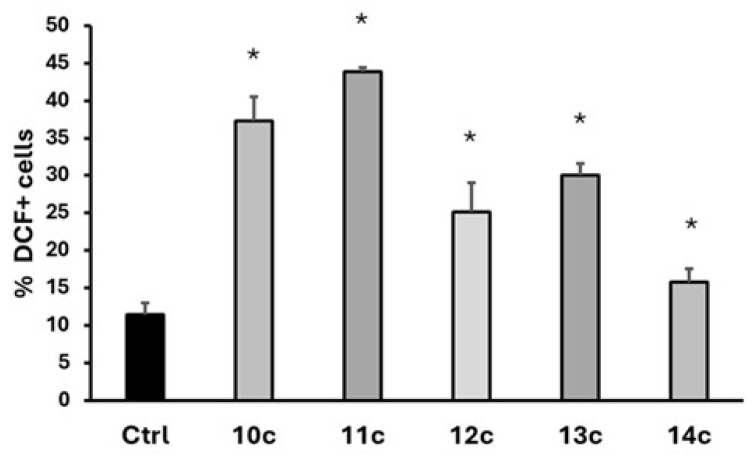
The effect of the compound’s treatment on intracellular ROS generation. Cells were treated with compounds (respective IC_50_ values for each compound for 24 h), and intracellular ROS levels were determined in the human cancer cell line MCF-7 by flow cytometry using dichloro-fluorescein diacetate (DCFH_2_-DA). * *p* < 0.05 versus control cells (Ctrl). The data represent the means ± S.D. of at least three experiments with triplicate samples.

An increase in intracellular ROS may be one of the factors that contribute to the cytotoxicity of certain compounds in cancer cell lines [35,36,37,38,39]. In fact, ROS and mitochondrial damage are often associated with the induction of cell death. Therefore, ROS levels were evaluated by flow cytometry after 24 h of treatment with compounds having the 3,4-dimethoxy pattern in the aromatic ring (**10c**, **11c**, **12c**, **13c**, and **14c**), at their respective IC_50_ values, using the fluorescent probe dichlorofluorescein diacetate (DCFH_2_-DA). DCF fluorescence was then measured by flow cytometry. The difference in the percentage of DCF-positive cells between treated and control cells was used to indicate an increase in intracellular ROS, as shown in Figure 4. For all of these compounds, treatment significantly increased the fluorescence in MCF-7 cells compared to the control, indicating an increase in intracellular ROS levels in the MCF-7 cell line (Figure 4). Compound **11c** was the most effective in this assay.

### 2.4. Mitochondrial Membrane Potential Analysis

**Figure 5:**
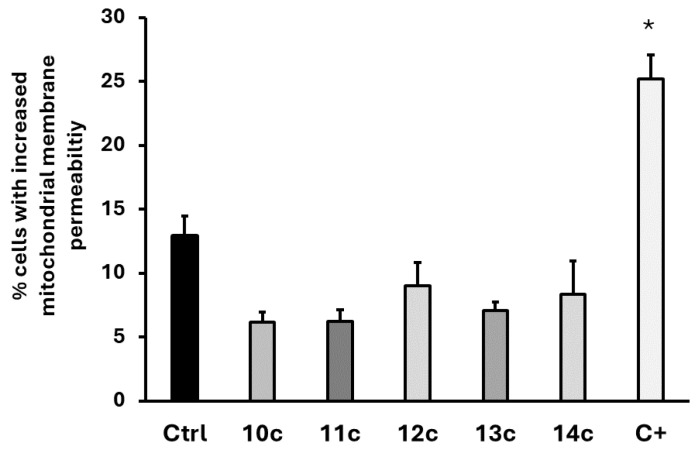
The effect of the compound’s treatment on mitochondrial membrane potential. Cells were treated with **10c**, **11c**, **12c**, **13c**, and **14c** (respective IC_50_ values for each compound for 24 h), and mitochondrial membrane permeability was determined in the human breast cancer cell line MCF-7 by flow cytometry using rhodamine 123. * *p* < 0.05 versus control cells (Ctrl). The data represent the means ± S.D. of at least three experiments with triplicate samples.

To determine whether the toxic effects of selected sesquiterpenes (**10c**, **11c**, **12c**, **13c**, and **14c**) are due to the production of ROS and subsequent apoptosis, the mitochondrial membrane potential was measured. With this aim, a protocol using rhodamine 123 staining and flow cytometry was established to study the effect of the selected compounds [40]. The accumulation of rhodamine 123 in mitochondria is associated with the maintenance of the electrochemical gradient in mitochondria [40]. Daunorubicin was used as a positive control (C+). The results are presented as the percentage of cells with increased mitochondrial membrane permeability. The data indicate that the selected compounds did not increase the mitochondrial membrane permeability in the MCF-7 cell line at the concentrations tested, showing that mitochondrial functionality is not affected (Figure 5).

### 2.5. Determination of Caspases 3/7 Activation (Caspase-Glo 3/7 Assay)

**Figure 6:**
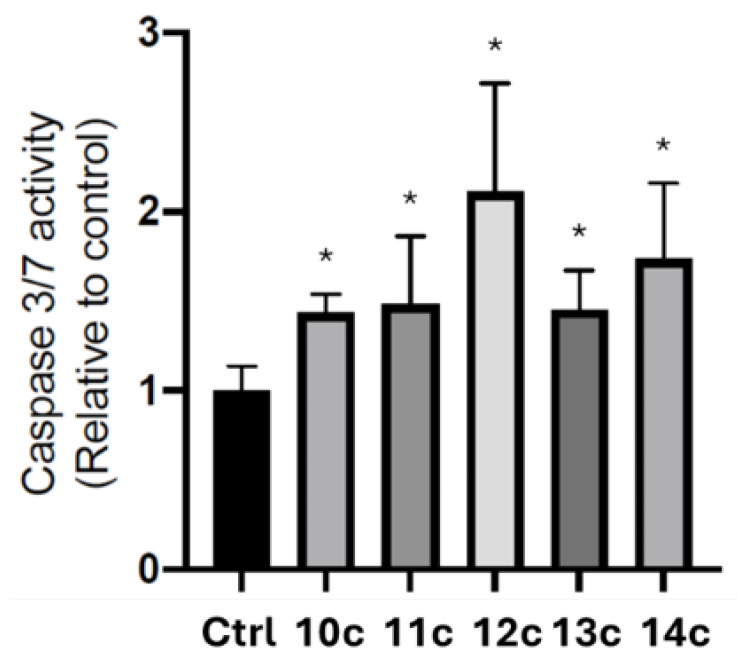
The effect of the compounds on caspases-3/7 activity in cells from the human breast cancer cell line MCF-7. Caspases-3/7 activity was determined in MCF-7 cells following treatment with the compounds (50 μM) for 24 h. The data are reported as the ratio of activity in treated cells vs. the control (1% ethanol), which were arbitrarily assigned a unitary value. * *p* < 0.05 versus control cells (Ctrl). The data are reported as the mean values ± S.D. of three different experiments with samples in triplicate.

Since ROS levels were increased in the MCF-7 cancer cell line in response to the compounds, it is important to determine whether this effect is associated with the onset of apoptotic cell death. To identify the type of cell death triggered by the most effective sesquiterpene derivatives (**10c**, **11c**, **12c**, **13c**, and **14c**), we assessed their aptitude for activating caspases 3/7 on MCF-7 cells, thereby inducing apoptosis. Cells were treated with the selected compounds at 50 μM for 24 h, incubated in situ with the Caspase-Glo 3/7 marker assay, and then analyzed in a luminometer. As shown in Figure 6, exposure to the compounds considerably enhanced caspases-3/7 activity in the MCF-7 cell line. These results suggest the activation of the apoptotic pathway.

### 2.6. Assays of TOP 1/2 Activity

**Figure 7:**
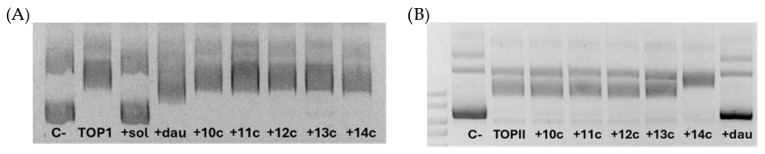
The effect of sesquiterpene aryl esters on TOP1- and TOP2-induced DNA relaxation was assessed. (**A**) The TOP1 inhibition assay evaluated the compounds (**10c**, **11c**, **12c**, **13c**, and **14c**) at a concentration of 20 μM in a DNA relaxation assay. (**B**) The TOP2 inhibition assay examined the same compounds at the same concentration in a DNA relaxation assay. The solvent used to dilute the compounds in the study and the absence of TOP1 or 2 are referred to as Sol. The presence of TOP1 or 2 indicates the assay conditions in the absence of compounds. C—the negative control without TOP1 or 2. Dau = daunorubicin as positive control of TOP1/2 inhibition.

The ability to relax DNA conformations is a central function of topoisomerase enzymes. Several studies have associated some proapoptotic compounds with the ability to inhibit topoisomerase enzymes. The ability of the selected compounds to inhibit TOP1 or TOP2 activity was determined. In this experiment, daunorubicin was used as a positive control for TOP1/2 inhibition, as previously reported [41]. As shown in Figure 7A, the selected compounds do not inhibit TOP1-induced DNA relaxation at 20 μM. Figure 7B shows that only daunorubicin and **14c** modified the TOP2-induced DNA relaxation pattern. It is important to note the potential for cardiotoxicity associated with some TOP2 inhibitors, which highlights the relevance of assessing compound **14c**’s interaction with TOP2B. While the current study focused on general TOP2 inhibition and did not specifically test compound **14c** against TOP2B, the isoform selectivity of **14c** is an important consideration given the clinical significance of TOP2B-related cardiotoxicity. Future studies should include assays to evaluate the specific activity of **14c** against both TOP2A and TOP2B to provide a more comprehensive safety profile, as isoform-selective TOP2 inhibitors may offer a therapeutic advantage by reducing adverse effects.

### 2.7. Molecular Docking and Molecular Dynamics Studies

**Figure 8:**
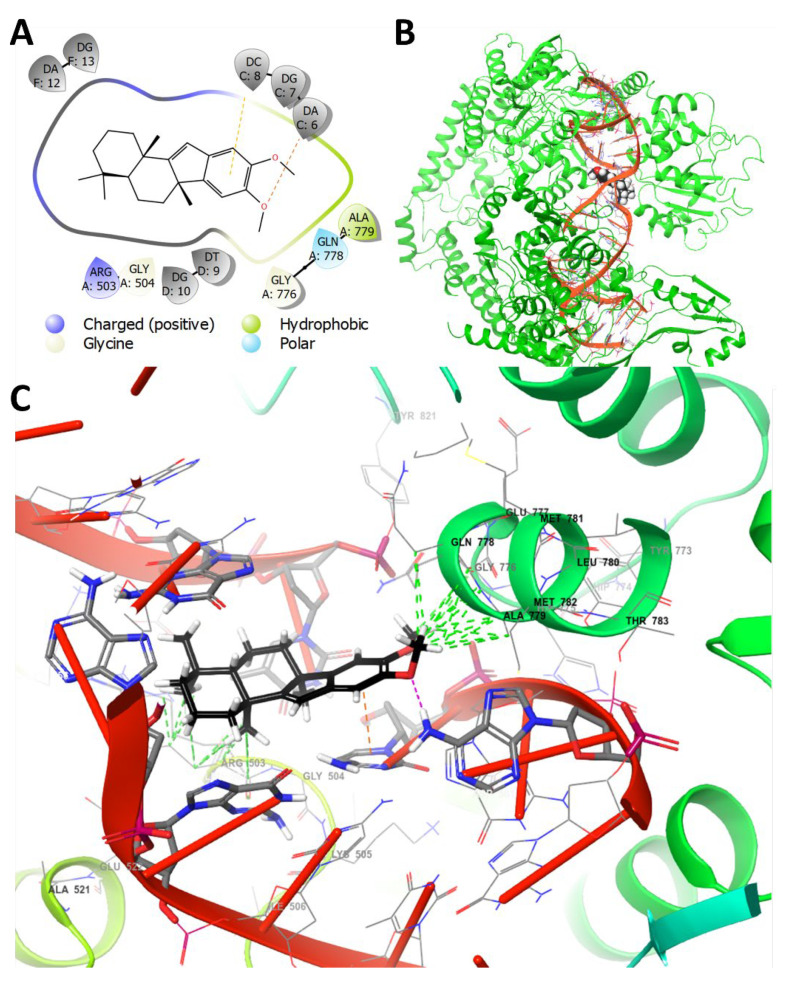
The molecular docking result for compound **14c** and DNA-TOP2 complex (PDB ID: 3QX3). (**A**) Two-dimensional view. The environment around a 4 Å distance from the ligand is depicted. (**B**) A far 3D view of compound **14c** in the complex. The molecule is inserted between the protein and DNA chain. (**C**) A close 3D view for the best pose. Hydrophobic interactions are depicted in green; π-stacking is shown with the orange dotted line, and the hydrogen bond is shown with the red dotted line.

Since compound **14c** was the most promising of the series in terms of cytotoxicity and selectivity on MCF-7 cells as well as in TOP2 inhibition, docking studies were carried out on this enzyme (PDB ID: 3QX3). The aim was to evaluate the potential intercalation capacity of the compound in the DNA-TOP2 complex, as well as to explore possible chemical modifications that could be made to this type of structure to generate new derivatives with higher inhibitory potency. Figure 8 shows the best binding mode of **14c** in the complex in both 2D (Figure 8A) and 3D (Figure 8B,C) representations.

Figure 8A shows the environment of nitrogenous bases and amino acid residues around compound **14c** within a 4 Å radius. The blue line represents polar and positively charged regions. The presence of an Arg503 residue suggests that the incorporation of electronegative or negatively ionized atoms at pH 7.0 would be a favorable chemical modification to increase affinity. It is therefore suggested that the insertion of fluorine atoms and carboxylate groups be investigated. The green line represents the hydrophobic environment around the methoxy groups attached to the benzene ring. Residues Ala779, Gln778, and Gly776 are important in the interaction with the molecule. Additionally, the compound closely aligns with several DNA nitrogen bases (DA), such as DA6, DG7, DC8, DT9, DG10, DA12, and DG13, with which it can establish p-stacking interactions. This suggests a capacity for steric perturbation by compound **14c** in the DNA–topoisomerase complex.

The 3D representation (Figure 8B,C) shows that molecule **14c** is inserted between the nitrogen bases of the DNA, disrupting the DNA–topoisomerase interaction. The benzo-cyclopentadiene core of **14c** is positioned between the bases, while hydrophobic contacts with the protein are established by methoxy groups and the drimane moiety (Figure 8C). The benzene ring is found to be 3.92 Å away from the DC8 base, interacting through potential π-stacking. Additionally, an oxygen atom of methoxy is situated 3.16 Å away from the NH_2_ group of the DA6 base, potentially forming a hydrogen bond or dipole–dipole interaction. Multiple hydrophobic contacts with Gly779, Gln778, and Ala779 are formed by the methoxy groups. Furthermore, multiple hydrophobic contacts with the side chain of Arg503 are formed by the axial methyl groups of the drimane system.

**Figure 9:**
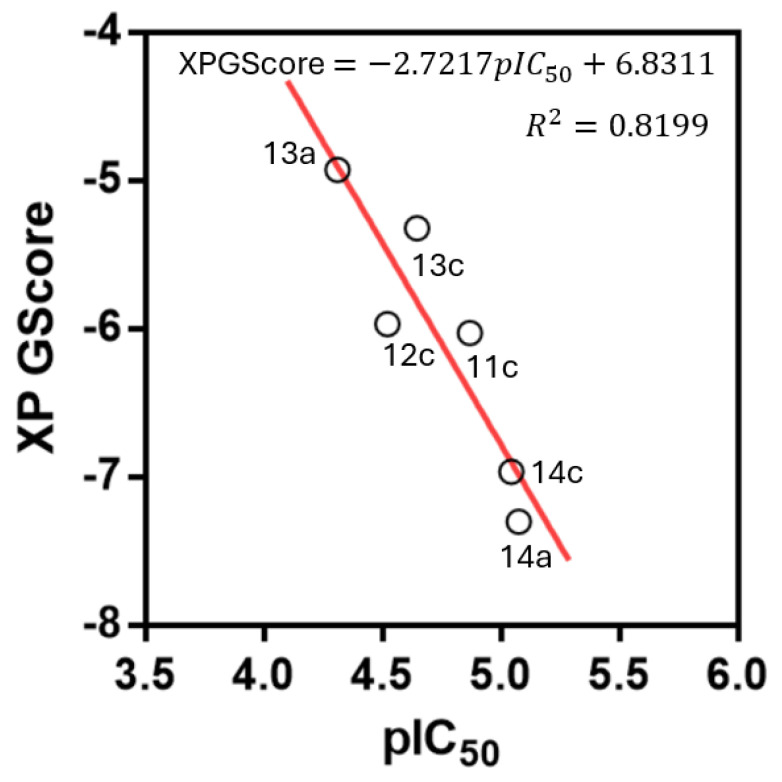
Correlation between pIC_50_ and XP GScore. The equation for the line is shown, as well as the r^2^ value for the linear regression.

To obtain comparative information with **14c**, the docking of compound **13c** in the DNA-TOP2 complex was performed. Compound **13c** showed the best IC_50_ value in MCF-7 cells (22.5 µM) within the family of non-tetracyclic molecules. However, **13c** showed no inhibition of the TOP2-complex inhibition according to Figure 7B. Compound **13c** showed an XP GScore of −5.321 kcal/mol, compared to −6.964 kcal/mol for **14c**. This difference could be attributed to the fact that **13c** failed to properly intercalate between the nitrogenous bases of DNA, as shown in Figures S1 and S2, which show several unfavorable steric contacts between the compound and the enzyme complex. Additionally, the docking study of **14c** in the DNA-TOP1 complex was performed (PDB ID: 1K4T) to explain why **14c** did not show significant inhibition of the DNA-TOP1 complex compared to TOP2. In Figures S3 and S4 show the best docking pose for **14c** with TOP1, where this molecule is oriented parallel to the DNA double strand and fails to intercalate between the nitrogenous bases. In Figure 9, we present the correlation between the pIC_50_ values and the XP GScore. Linear regression was observed for the most active compounds of the series: **11c**, **12c**, **13a**, **13c**, **14a**, and **14c**. Only compound **10c** did not fit the correlation.

**Figure 10:**
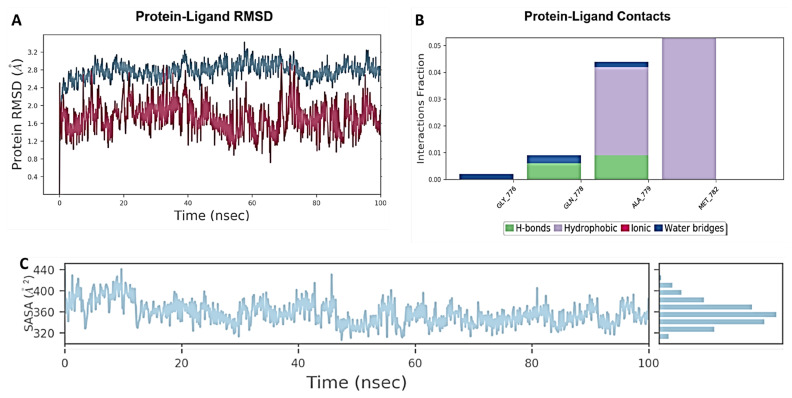
The molecular dynamics (100 ns) results for the topoisomerase–DNA complex and **14c**. (**A**) The RMSD plot. The protein RMSD is shown in blue, and the complex ligand RMSD is shown in red. (**B**) The protein–ligand contacts’ plot. (**C**) The Solvent Accessible Surface Area (SASA) plot.

To further assess the feasibility of this potential binding mode of **14c** in TOP2, a molecular dynamics study was performed. Figure 10 shows the results of the 100 ns molecular dynamics for **14c** and the DNA-TOP2 complex. The complex underwent an oscillation averaging between 1.2 and 2.4 Å (Figure 10A). This relative stability of the RMSD fluctuation can be explained by the high rigidity of the compound. The primary interactions over time were seen with Gly777 via a water bridge; with Gln778 through hydrogen and water bridges; with Ala779 via hydrogen and water bridges; and finally, with Met782 through hydrophobic interactions (Figure 10B). Regarding the docking results, a new interaction with Met782 was observed, while contacts with Arg503 were lost, possibly due to the solvation effect of this amino acid. Finally, the Solvent Accessible Surface Area (SASA) graph (Figure 10C) indicates that the molecule experienced a reduction in solvation degree from 60 ns onwards, with an average value of 360 Å^2^. Based on these results, it could be suggested that the perturbation of the complex by compound **14c** occurs through steric and hydrophobic factors, suggesting a function as a topoisomerase 2 poison, although it is also possible to hypothesize a potential and more favorable allosteric interaction between the protein and the compound.

### 2.8. Calculated Physicochemical Properties and ADME Parameters

**Figure 11:**
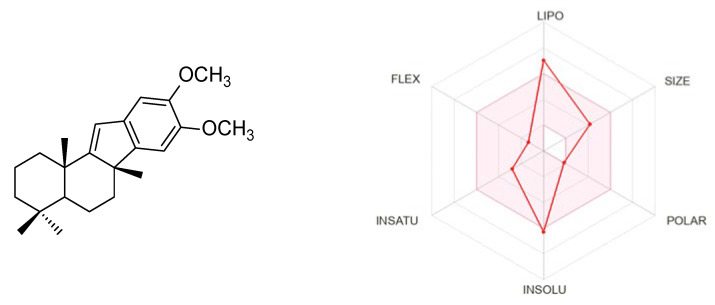
A radar chart illustrating the bioavailability of **14c**. The pink range represents the optimal values for each property related to oral bioavailability. The forecasted properties, marked in red, include flexibility (FLEX), lipophilicity (LIPO), solubility (INSOLU), size (SIZE), polarity (POLAR), and saturation (INSATU). If all parameters fall within the designated range, it is anticipated that the compound will have good oral absorption.

One key aspect to consider in the search for antitumor drugs is their pharmacokinetic properties. Not only are cytotoxic properties, the mechanism of action, and cell death important, but physicochemical properties that influence drug absorption, distribution, metabolism, and excretion (ADME) must also be considered. By doing so, these properties can be taken into account during the optimization of a bioactive compound, ultimately making it a suitable candidate for preclinical studies [42]. To assess the physicochemical properties of compound **14c**, the free online platform SwissADME (http://www.swissadme.ch/index.php, accessed on 15 March 2025) was utilized in accordance with the Lipinski and Veber rules. According to these criteria, compound **14c** exhibited promising permeability and bioavailability due to its HBD, HBA, and MW values. However, its cLogP value was found to be close to the optimum limit [43]. Additionally, **14c** has a topological polar surface area (TPSA) and a number of rotatable bonds (NRB) value that are both less than 140 Å^2^ and ≤10 NRB, respectively (Table 2), and these values meet the criteria set forth by Veber’s rules [44]. Based on the results provided by both models, it can be concluded that **14c** has a high potential for penetrating cell membranes and demonstrates good oral absorption. Furthermore, the bioavailability radar plot generated by the SwissADME platform suggests that **14c** is likely to exhibit good oral absorption (Figure 11). Predicted ADME and toxicity parameters are summarized in Table 2. Compound **14c** fits well within Lipinski’s rule of five for good oral bioavailability, with a low number of hydrogen bond acceptors (HBAs) and hydrogen bond donors (HBDs), a topological polar surface area (TPSA) below 140 Å^2^, and fewer than 10 rotatable bonds (NRB). The only exception is the cLogP value, which is slightly above 5.0.

Regarding toxicity, compound **14c** is predicted not to inhibit the hERG potassium ion channel. Moreover, it does not contain the typical pharmacophoric features of known hERG inhibitors, which usually involve basic and positively charged nitrogen atoms rather than acidic or neutral functionalities.

Finally, the compound is expected to be a substrate only of CYP3A4, suggesting good metabolic stability against first-pass metabolism. As for P-glycoprotein (Pgp), which acts as an efflux pump, it would be desirable for the compound to inhibit this transporter to enhance intestinal absorption and achieve higher intracellular concentrations in neoplastic cells values of ADME and toxicity.

## 3. Materials and Methods

### 3.1. General Information

The reagents and solvents used in the experiments were purchased from commercial suppliers and were not further purified. HPLC-grade solvents were purchased from Merck (Darmstadt, Germany) and Fisher Scientific (Branchburg, NJ, USA). The melting points (m.p.) were measured on an SMP3 apparatus (Stuart Scientific, Merck KGaA, Darmstadt, Germany) and were uncorrected. The ^1^H-, ^13^C-, ^13^C DEPT-135, *gs* 2D HSQC, and *gs* 2D HMBC NMR spectra were recorded in CDCl_3_ solutions and were referenced to the residual peaks of CHCl_3_ at δ = 7.26 ppm and δ = 77.00 ppm for ^1^H and ^13^C, respectively, on an Advance Neo 400 Digital NMR spectrometer (Bruker, Rheinstetten, Germany) operating at 400.1 MHz for ^1^H and 100.6 MHz for ^13^C. Chemical shifts are reported in δ ppm, and coupling constants (*J*) are given in Hz. The HRMS-ESI data of the final products were acquired in positive ion mode with a scanning range of *m*/*z* 300.00–1510.40 and a resolution of 140.000 using a Bruker Compact QTOF MS + Elute UHPLC (Bruker, Karlsruhe, Germany) with a constant nebulizer temperature of 250 °C. The samples were dissolved in acetonitrile and injected directly into the ESI source through an injection valve and syringe pump at a flow rate of 5 µLmin^−1^. Thin layer chromatography (TLC) was performed using Merck GF-254 type 60 silica gel, a 0.25 mm layer was used, and TLC spots were detected by heating after spraying with 10% H_2_SO_4_ in H_2_O. Chromatographic separations were carried out by conventional column on silica gel 60 (230–400 mesh) using hexane–EtOAc mixtures of increasing polarity. The compound (-)-drimenol (**7**) was obtained from the hexane extract of the bark of *Drimys winteri Forst* [23]. The details of the synthetic procedures and characterization of all the synthesized compounds are available in the Supplementary Material.

### 3.2. Synthetic Procedures

#### 3.2.1. Synthesis of Drimenal (**8**)

PCC (500 mg, 2.32 mmol) was slowly added to a solution of (−)-drimenol **7** (300 mg, 1.35 mmol) in CH_2_Cl_2_ (5 mL), and the mixture was stirred at 0 °C for 20 min. The mixture was filtrated through a short silica gel filter column with a mixture of hexane/ethyl acetate (9:1). The solvent was evaporated under reduced pressure to obtain **8** (yield 78%). The NMR spectra signals agree with the reported values [45]. ^1^H NMR (400 MHz, CDCl_3_, δ, ppm): 0.85 (3H, s, CH_3_-14), 0.90 (3H, s, CH_3_-13), 1.04 (3H, s, CH_3_-15), 1.65 (3H, bs, CH_3_-12), 2.56 (1H, bs, H-9), 5.67 (1H, bs, H-7), 9.66 (1H, d, *J* = 5.16 Hz, CHO). ^13^C NMR (101 MHz, CDCl_3_, δ, ppm): 15.61 (C-15), 18.18 (C-2), 21.52 (C-14), 21.98 (C-12), 23.55 (C-6), 32.93 (C-4), 33.20 (C-13), 36.91 (C-10), 40.26 (C-1), 41.90 (C-3), 48.95 (C-5), 67.48 (C-9), 125.38 (C-7), 127.68 (C-8), 206.61 (C-11).

#### 3.2.2. The General Procedure for the Synthesis of Alcohols **10a**–**d**

The procedure used by Alvarez-Manzaneda et al. [45] was followed with some modifications. A 1.7 M solution of *tert*-butyllithium in pentane (2.4 mL) at −80 °C was dripped into a solution of the correspondent bromo benzene derivative **9a**–**d** (3.68 mmol) in THF (25 mL) under an argon atmosphere. After stirring for 1.5 h at this temperature, drimenal (0.4 g, 1.82 mmol) was added, and the mixture was further stirred for 1.5 h at −80 °C. Water (12 mL) was added, the mixture was diluted with ether (80 mL), and the organic phase was washed with brine, dried, and concentrated to obtain a crude, which was taken to silica gel chromatography to afford the product.

(1*S*)-(2,4-Dimethoxyphenyl)((1*S*,8a*S*)-2,5,5,8a-tetramethyl-1,4,4a,5,6,7,8,8a-octahydronaphthalen-1-yl) methanol (**10a**).

White solid, yield: 78.4%, m.p.: 81.9–82.5 °C. ^1^H NMR (400 MHz, CDCl_3_, δ, ppm): 0.81 (3H, s, CH_3_-14), 0.86 (3H, s, CH_3_-13), 1.05 (3H, s, CH_3_-15), 1.48 (3H, s, CH_3_-12), 2.50 (1H, bs, CH-9), 3.72 (3H, s, OCH_3_-4′), 3.73 (3H, s, OCH_3_-2′), 5.15 (1H, d, *J* = 4.2 Hz, H-11), 5.50 (1H, m, CH-7), 6.34 (1H, d, *J* = 2.4 Hz, CH-3′), 6.39 (1H, dd, *J* = 8.5, 2.5 Hz, CH-5′), 7.42 (1H, dd, *J* = 8.5, 1.0 Hz, CH-6′). ^13^C (101 MHz, CDCl_3_, δ, ppm): 15.21 (C-15), 18.95 (C-2), 22.42 (C-13), 23.39 (C-6), 24.61 (C-12), 32.97 (C-4), 33.51 (C-14), 37.68 (C-10), 39.95 (C-1), 42.27 (C-3), 50.13 (C-5), 54.61 (OCH_3_-4′), 55.22 (OCH_3_-2′), 58.06 (C-9), 67.03 (C-11), 98.07 (C-3′), 103.27 (C-5′), 126.26 (C-1′), 126.41 (C-7), 128.09 (C-6′), 133.24 (C-8), 156.76 (C-2′), 159.36 (C-4′). HRMS for (C_23_H_34_O_3_ [M + H]^+^). Calcd: 359.2581. Found: 359.2576.

(1*S*)-(2,5-Dimethoxyphenyl)((1*S*,8a*S*)-2,5,5,8a-tetramethyl-1,4,4a,5,6,7,8,8a-octahydronaphthalen-1-yl)methanol (**10b**).

White solid, yield: 82.04%, m.p.: 83.9–85 °C. ^1^H NMR (400 MHz, CDCl_3_, δ, ppm): 0.88 (3H, s, CH_3_-14), 0.93 (3H, s, CH_3_-13), 1.12 (3H, s, CH_3_-15), 1.54 (3H, s, CH_3_-12), 2.62 (1H, d, *J* = 3.8 Hz, CH-9), 3.76 (6H, s, OCH_3_-2′ + OCH_3_-5′) 5.23 (1H, s, H-11), 5.58 (1H, m, CH-7), 6.71 (1H, dd, *J* = 8.8, 2.8 Hz, CH-4′), 6.74 (1H, d, *J* = 8.7 Hz, CH-3′), 7.24 (1H, dd, *J* = 2.8, 0.8 Hz, CH-6′). ^13^C NMR (101 MHz, CDCl_3_, δ, ppm): 15.39 (C-15), 19.03 (C-2), 22.53 (C-13), 23.45 (C-6), 24.76 (C-12), 33.05 (C-4), 33.61 (C-14), 37.77 (C-10), 39.97 (C-1), 42.30 (C-3), 50.15 (C-5), 55.15*, 55.74* 58.22 (C-9), 67.43 (C-11), 110.86 (C-20), 111.48 (C-19), 114.47 (C-17), 126.72 (C-7), 133.14 (C-8), 135.26 (C-16), 150.19^#^, 153.19^#^. *Interchangeable signals: OCH_3_-3′ + OCH_3_-5′. ^#^Interchangeable signals: C-3′ + C-5′. HRMS for (C_23_H_34_O_3_ [M + H]^+^). Calcd: 359.2581. Found: 341.2488 ([M + H]^+^ − H_2_O).

(1*S*)-(3,4-Dimethoxyphenyl)((1*S*,8a*S*)-2,5,5,8a-tetramethyl-1,4,4a,5,6,7,8,8a-octahydronaphthalen-1-yl) methanol (**10c**).

White solid, yield: 79.91%, m.p.: 83–85.2 °C. ^1^H NMR (400 MHz, CDCl_3_, δ, ppm): 0.83 (3H, s, CH_3_-14), 0.87 (3H, s, CH_3_-13), 1.05 (3H, s, CH_3_-15), 1.37 (3H, s, CH_3_-12), 2.38 (1H, bs, CH-9), 3.80 (3H, s, CH_3_-4′), 3.82 (3H, s, OCH_3_-3′), 5.01 (1H, d, *J* = 4.6 Hz, H-11), 5.51 (1H, m, CH-7), 6.75 (1H, d, *J* = 8.4 Hz, CH-5′), 6.86 (1H, ddd, *J* = 8.4, 2.2, 1.1 Hz, CH-6′), 6.95 (1H, d, *J* = 2.1 Hz, CH-2′). ^13^C (101 MHz, CDCl_3_, δ, ppm): 15.39 (C-15), 18.79 (C-2), 22.28 (C-13), 23.28 (C-6), 23.86 (C-12), 32.89 (C-4), 33.40 (C-14), 36.81 (C-10), 40.74 (C-1), 42.08 (C-3), 50.09 (C-5), 55.82 (OCH_3_-4′), 55.83 (OCH_3_-3′), 62.45 (C-9), 69.09 (C-11), 108.74 (C-2′), 110.67 (C-5′), 116.95 (C-6′), 126.84 (C-7), 132.13 (C-8), 139.38 (C-1′), 147.01 (C-4′), 148.46 (C-3′). HRMS for (C_23_H_34_O_3_ [M + H]^+^). Calcd: 359.2581. Found: 341.2464 ([M + H]^+^ − H_2_O).

(1*S*)-(3,5-Dimethoxyphenyl)((1*S*,8a*S*)-2,5,5,8a-tetramethyl-1,4,4a,5,6,7,8,8a-octahydronaphthalen-1-yl)methanol (**10d**).

White solid, yield: 90.6%, m.p.: 83.5–86 °C.^1^H NMR (400 MHz, CDCl_3_, δ, ppm): 0.89 (3H, s, CH_3_-14), 0.93 (3H, s, CH_3_-13), 1.10 (3H, s, CH_3_-15), 1.46 (3H, s, CH_3_-12), 2.46 (1H, bs, CH-9), 3.80 (6H, s, OCH_3_-3′ + OCH_3_-5′), 5.03 (1H, s, CH-11), 5.58 (1H, m, CH-7), 6.30 (1H, t, *J* = 2.3 Hz, CH-4′), 6.61 (2H, dd, *J* = 2.3, 1.0 Hz, CH-6′ + CH-2′). ^13^C NMR (101 MHz, CDCl_3_, δ, ppm): 15.48 (C-15), 18.85 (C-2), 22.35 (C-13), 23.34 (C-6), 23.84 (C-12), 32.95 (C-4), 33.48 (C-14), 36.84 (C-10), 40.78 (C-1), 42.14 (C-3), 50.15 (C-5), 55.31 (OCH_3_-3′ + OCH_3_-5′), 62.55 (C-9), 69.47 (C-11), 97.45 (C-4′), 103.53 (C-6′ + C-2′), 126.86 (C-7), 132.16 (C-8), 149.81 (C-1′), 160.50 (C-5′ + C-3′). HRMS for (C_23_H_34_O_3_ [M + H]^+^). Calcd: 359.2581. Found: 341.2483 ([M + H]^+^ − H_2_O).

#### 3.2.3. The General Procedure for the Synthesis of Reduced Compounds **11a**–**d**

The reduction procedure used by Barclay et al. [45] was followed with some modifications. In a solution of the alcohol **10a**–**d** (0.43 mmol) in anhydrous DCM (10 mL) over an ice bath, 0.8 mL (5 mmol) of triethylsilane and 0.02 mL (0.26 mmol) of trifluoroacetic acid were dripped under an argon atmosphere. After 30 min, the ice bath was removed, the mixture was left in agitation, and the reaction was monitored by TLC. When it was found that no starting product was present, the mixture was diluted with ether (30 mL), and the organic phase was washed with saturated sodium bicarbonate solution and brine, and then dried and concentrated to obtain a crude, which underwent silica gel chromatography to afford the product.

(4a*S*,5*S*)-5-(2,4-Dimethoxybenzyl)-1,1,4a,6-tetramethyl-1,2,3,4,4a,5,8,8a-octahydronaphthalene (**11a**).

White solid, yield: 99.5%, m.p.: 108.6–190.2 °C. ^1^H NMR (400 MHz, CDCl_3_, δ, ppm): 0.88 (3H, s, CH_3_-14), 0.89 (3H, s, CH_3_-13), 0.91 (3H, s, CH_3_-15), 1.44 (3H, bs, CH_3_-12), 2.6 (1H, m, CH_2_-11), 3.79 (3H, s, OCH_3_-4′), 3.80 (3H, s, OCH_3_-2′), 5.36 (1H, m, H-7), 6.43 (2H, m, CH-3′ + CH-5′), 7.12 (1H, d, *J* = 7.9 Hz, CH-6′). ^13^C NMR (101 MHz, CDCl_3_, δ, ppm): 13.85 (C-13), 19.03 (C-2), 22.04 (C-15), 22.43 (C-12), 23.82 (C-6), 25.71 (C-11), 33.11 (C-4), 33.34 (C-14), 36.95 (C-10), 39.51 (C-1), 42.37 (C-3), 50.38 (C-5), 54.12 (C-9), 55.20 (OCH_3_-4′), 55.31 (OCH_3_-2′), 98.34 (C-3′), 103.83 (C-5′), 121.95 (C-7), 124.54 (C-1′), 129.74 (C-6′), 136.19 (C-8), 157.95 (C-2′), 158.53 (C-4′). HRMS for (C_23_H_34_O_2_ [M + H]^+^). Calcd: 343.2632. Found: 343.2625.

(4a*S*,5*S*)-5-(2,5-Dimethoxybenzyl)-1,1,4a,6-tetramethyl-1,2,3,4,4a,5,8,8a-octahydronaphthalene (**11b**).

Colorless oil, yield: 95%. ^1^H NMR (400 MHz, CDCl_3_, δ, ppm): 0.88 (3H, s, CH_3_-14), 0.89 (3H, s, CH_3_-13), 0.91 (3H s, CH_3_-15), 1.44 (3H, bs, CH_3_-12), 2.36 (1H, d, *J* = 9.3 Hz, CH-9), 2.57 (1H, m, CH_2_-11), 2.72 (1H, dd, *J* = 15.4, 9.4 Hz, CH_2_-11), 3.77 (3H, s, OCH_3_-5′), 3.78 (3H, s, OCH_3_-2′), 5.37 (1H, m, CH-7), 6.66 (1H, dd, *J* = 8.9, 3.0 Hz, CH-4′), 6.74 (1H, d, *J* = 8.7 Hz, CH-3′), 6.84 (1H, d, *J* = 3.0 Hz, CH-6′). ^13^C NMR (101 MHz, CDCl_3_): 14.06 (C-13), 19.13 (C-2), 22.15 (C-15), 22.41 (C-12), 23.93 (C-6), 26.36 (C-11), 33.22 (C-4), 33.45 (C-14), 37.03 (C-10), 39.68 (C-1), 42.47 (C-3), 50.48 (C-5), 54.51 (C-9), 55.81 (OCH_3_-5′), 56.00 (OCH_3_-2′), 110.05 (C-4′), 111.31 (C-3′), 116.37 (C-6′), 122.12 (CH-7), 133.91 (C-1′), 136.06 (C-8), 151.69 (C-2′), 153.55 (C-5′). HRMS for (C_23_H_34_O_2_ [M + H]^+^). Calcd: 343.2632. Found: 343.2620.

(4a*S*,5*S*)-5-(3,4-Dimethoxybenzyl)-1,1,4a,6-tetramethyl-1,2,3,4,4a,5,8,8a-octahydronaphthalenene (**11c**).

Colorless oil, yield: 72.9%. ^1^H NMR (400 MHz, CDCl_3_, δ, ppm): 0.87 (3H, s, CH_3_-14), 0.88 (3H, s, CH_3_-13), 0.91 (3H, s, CH_3_-15), 1.47 (3H, s, CH_3_-12), 2.20 (1H, m, CH-9), 2.85 (1H, m, CH_2_-11), 3.86 (3H, s, OCH_3_-4′), 3.87 (3H, s, OCH_3_-3′), 5.38 (1H, m, CH-7), 6.76 (1H, s, CH-2′), 6.77 (2H, d, *J* = 1.0 Hz, CH-5′ + CH-6′). ^13^C NMR (101 MHz, CDCl_3_, δ, ppm): 13.84 (C-14), 18.88 (C-2), 21.88 (C-15), 23.21 (C-12), 23.68 (C-6), 29.61 (C-4), 33.01 (C-11), 33.17 (C-13), 36.67 (C-10), 39.76 (C-1), 42.20 (C-3), 50.17 (C-5), 55.66 (OCH_3_-4′), 55.79 (OCH_3_-3′), 55.82 (C-9), 110.97 (C-2′), 112.00 (C-5′), 120.44 (C-6′), 122.23 (C-7), 135.54 (C-8), 136.70 (C-1′), 146.68 (C-4′), 148.55 (C-3′). HRMS for (C_23_H_34_O_2_ [M + H]^+^). Calcd: 343.2632. Found: 343.2627.

(4a*S*,5*S*)-5-(3,5-Dimethoxybenzyl)-1,1,4a,6-tetramethyl-1,2,3,4,4a,5,8,8a-octahydronaphthalene (**11d**).

Colorless oil, yield: 36%. ^1^H NMR (400 MHz, CDCl_3_, δ, ppm): 0.88 (3H, s, CH_3_-14), 0.89 (3H, s, CH_3_-13), 0.91 (3H s, CH_3_-15), 1.44 (3H, bs, CH_3_-12), 2.36 (1H, d, *J* = 9.3 Hz, CH-9), 2.57 (1H, m, CH_2_-11), 2.72 (1H, dd, *J* = 15.4, 9.4 Hz, CH_2_-11), 3.77 (3H, s, CH_3_-5′), 3.78 (3H, s, CH_3_-3′), 5.37 (1H, m, CH-7), 6.66 (1H, dd, *J* = 8.9, 3.0 Hz, CH-4′), 6.74 (1H, d, *J* = 8.7 Hz, CH-2′), 6.84 (1H, d, *J* = 3.0 Hz, CH-6′). ^13^C NMR (101 MHz, CDCl_3_): 14.06 (C-13), 19.13 (C-2), 22.15 (C-15), 22.41 (C-12), 23.93 (C-6), 26.36 (C-11), 33.22 (C-4), 33.45 (C-14), 37.03 (C-10), 39.68 (C-1), 42.47 (C-3), 50.48 (C-5), 54.51 (C-9), 55.81 (OCH_3_-5′), 56.00 (OCH_3_-3′), 110.05 (C-4′), 111.31 (C-2′), 116.37 (C-6′), 122.12 (CH-7), 133.91 (C-1′), 136.06 (C-8), 151.69 (C-3′), 153.55 (C-5′).

#### 3.2.4. General Procedure for the Synthesis of Ketone Compounds **12a**–**d**

PCC (1.74 mmol) was slowly added to a solution of the alcohol **10a**–**d** (0.7 mmol) in CH_2_Cl_2_ (15 mL), and the mixture was stirred for 2 h. The mixture was filtrated through a short silica gel filter column with a mixture of hexane/ethyl acetate (9:1). The solvent was evaporated under reduced pressure to obtain the product.

(2,4-Dimethoxyphenyl)((1*S*,8a*S*)-2,5,5,8a-tetramethyl-1,4,4a,5,6,7,8,8a-octahydronaphthalen-1-yl)methanone (**12a**).

Yellow oil, yield: 94.5%. ^1^H NMR (400 MHz, CDCl_3_, δ, ppm): 0.85 (3H, s, CH_3_-14), 0.87 (3H, s, CH_3_-13), 0.89 (3H, s, CH_3_-15), 1.61 (3H, s, CH_3_-12), 3.82 (3H, s, OCH_3_-4′), 3.85 (3H, s, OCH_3_-2′), 4.29 (1H, m, CH-9), 5.56 (1H, dt, *J* = 3.9, 1.8 Hz, H-7), 6.43 (1H, d, *J* = 2.3 Hz, CH-3′), 6.49 (1H, dd, *J* = 8.6, 2.3 Hz, CH-5′), 7.45 (1H, d, *J* = 8.5 Hz, CH-6′). ^13^C (101 MHz, CDCl_3_, δ, ppm): 15.04 (C-15), 18.66 (C-2), 21.99 (C-12), 22.01 (C-13), 23.70 (C-6), 33.09 (C-4), 33.48 (C-14), 37.78 (C-10), 40.78 (C-1), 42.02 (C-3), 49.84 (C-5), 55.43 (OCH_3_-4′), 55.51 (OCH_3_-2′), 65.93 (C-9), 98.60 (C-3′), 105.02 (C-5′), 123.78 (C-7), 126.59 (C-1′), 131.66 (C-6′), 132.22 (C-8), 159.39 (C-4′), 163.42 (C-2′), 203.96 (C-11). HRMS for (C_23_H_32_O_3_ [M + H]^+^). Calcd: 357.2424. Found: 357.2435.

(2,5-Dimethoxyphenyl)((1*S*,8a*S*)-2,5,5,8a-tetramethyl-1,4,4a,5,6,7,8,8a-octahydronaphthalen-1-yl)methanone (**12b**).

Yellow oil, yield: 95%. ^1^H NMR (400 MHz, CDCl_3_, δ, ppm): 0.85 (3H, s, CH_3_-14), 0.87 (3H, s, CH_3_-13), 0.90 (3H, s, CH_3_-15), 1.62 (3H, bs, CH_3_-12), 3.77 (3H, s, CH_3_-5′), 3.83 (3H, s, OCH_3_-2′), 4.31 (1H, s, CH-9), 5.58 (1H, m, CH-7), 6.86 (1H, m, CH-4′), 6.95 (2H, m, CH-3′ + CH-6′). ^13^C NMR (101 MHz, CDCl_3_, δ, ppm): 14.98 (C-15), 18.58 (C-2), 21.89 (C-12 + C13), 23.62 (C-6), 33.03 (C-4), 33.39 (C-14), 37.77 (C-10), 40.84 (C-1), 41.88 (C-3), 49.68 (C-5), 55.78 (OCH_3_-5′), 56.07 (OCH_3_-2′), 66.31(C-9), 113.09 (C-4′), 114.10 (C-3′), 118.20 (C-6′), 124.02 (C-7), 131.86 (C-5′), 133.76 (C-2′), 151.87 (C-8), 153.51 (C-1′), 205.46 (C-11). HRMS for (C_23_H_32_O_3_ [M + H]^+^). Calcd: 357.2424. Found: 357.2434.

(3,4-Dimethoxyphenyl)((1*S*,8a*S*)-2,5,5,8a-tetramethyl-1,4,4a,5,6,7,8,8a-octahydronaphthalen-1-yl)methanone (**12c**).

Yellow oil, yield: 83%. ^1^H NMR (400 MHz, CDCl_3_, δ, ppm): 0.84 (3H, s, CH_3_-14), 0.85 (3H, s, CH_3_-13), 0.91 (3H, s, CH_3_-15), 1.45 (3H, s, CH_3_-12), 3.88 (6H, s, OCH_3_-3′ + OCH_3_-4′), 4.06 (1H s, CH-9), 5.55 (1H, m, CH-7), 6.81 (1H, d, *J* = 8.3 Hz, CH-5′), 7.45 (1H, d, *J* = 2.1 Hz, CH-2′), 7.53 (1H, m, CH-6′). ^13^C (101 MHz, CDCl_3_, δ, ppm): 14.44 (C-15), 18.59 (C-2), 21.64 (C-12), 21.95 (C-13), 23.73 (C-6), 33.07 (C-10), 33.43 (C-14), 37.64 (C-4), 41.83 (C-1), 42.10 (C-3), 49.88 (C-5) 55.91 (OCH_3_-4′), 55.96 (OCH_3_-3′), 60.40 (C-9), 109.62 (C-5′), 110.09 (C-2′), 123.00 (C-6′), 124.44 (C-7), 131.20 (C-8), 133.91 (C-1′), 148.96 (C-3′), 152.91 (C-4′), 200.93 (C-11). HRMS for (C_23_H_32_O_3_ [M + H]^+^). Calcd: 357.2424. Found: 357.2435.

(3,5-Dimethoxyphenyl)((1*S*,8a*S*)-2,5,5,8a-tetramethyl-1,4,4a,5,6,7,8,8a-octahydronaphthalen-1-yl)methanone (**12d**).

White solid, yield: 94.3%, m.p.: 75.6–76.4 °C. ^1^H NMR (400 MHz, CDCl_3_, δ, ppm): 0.85 (3H, s, CH_3_-14), 0.87 (3H, s, CH_3_-13), 0.90 (3H, s, CH_3_-15), 1.62 (3H, bs, CH_3_-12), 3.77 (3H, s, CH_3_-5′), 3.83 (3H, s, OCH_3_-2′), 4.31 (1H, s, CH-9), 5.58 (1H, m, CH-7), 6.86 (1H, m, CH-4′), 6.95 (2H, m, CH-3′ + CH-6′). ^13^C NMR (101 MHz, CDCl_3_, δ, ppm): 14.98 (C-15), 18.58 (C-2), 21.89 (C-12 + C13), 23.62 (C-6), 33.03 (C-4), 33.39 (C-14), 37.77 (C-10), 40.84 (C-1), 41.88 (C-3), 49.68 (C-5), 55.78 (OCH_3_-5′), 56.07 (OCH_3_-2′), 66.31(C-9), 113.09 (C-4′), 114.10 (C-3′), 118.20 (C-6′), 124.02 (C-7), 131.86 (C-5′), 133.76 (C-2′), 151.87 (C-8), 153.51 (C-1′), 205.46 (C-11). HRMS for (C_23_H_32_O_3_ [M + H]^+^). Calcd: 357.2424. Found: 357.2431.

#### 3.2.5. The General Procedure for the Synthesis of Diene Compounds **13a**–**d**

The procedure used by Domingo et al. [45] was followed with some modifications. Iodine (0.15 mmol) and DMSO (3.63 mmol) were added to a solution of the alcohol **10a**–**d** (0.73 mmol) in 7.25 mL of benzene. The mixture was left to reflux, and the reaction was monitored by TLC. When it was found that no starting product was present, the mixture was diluted with ethylic ether (30 mL), and the organic phase was washed with saturated sodium thiosulfate solution and brine, and then dried and concentrated to obtain a crude, which underwent silica gel chromatography to obtain the product.

(4a*S*)-5-((*E*)-2,4-Dimethoxybenzylidene)-1,1,4a,6-tetramethyl-1,2,3,4,4a,5,8,8a-octahydronaphthalene (**13a**).

White solid, yield: 71.3%, m.p.: 88.4–89.5 °C. ^1^H NMR (400 MHz, CDCl_3_, δ, ppm): 0.87 (3H, s, CH_3_-14), 0.95 (3H, s, CH_3_-13), 1.04 (3H, s, CH_3_-15), 1.46 (3H, bs, CH_3_-12), 3.80 (3H, s, OCH_3_-4′), 3.80 (3H, s, OCH_3_-2′), 5.55 (1H, m, CH-7), 6.21 (1H, s, CH-11), 6.40 (2H, m, CH-3′ + CH5′), 7.00 (1H, dd, *J* = 9.0, 0.8 Hz, CH-6′). ^13^C NMR (101 MHz, CDCl_3_, δ, ppm): 19.21 (C-2), 19.49 (C-15), 21.79 (C-13), 22.87 (C-12), 25.24 (C-6), 32.52 (C-14), 33.74 (C-4), 38.01 (C-1), 38.63 (C-10), 42.35 (C-3), 48.24 (C-5), 55.23*, 55.43*, 97.85 (C-3′), 103.71 (C-5′), 115.44 (C-11), 122.07 (C-1′), 128.05 (C-7), 131.83 (C-8), 131.86 (C-6′), 150.55 (C-9), 157.64 (C-2′), 159.58 (C-4′). *Interchangeable signs: (OCH_3_-2′), (OCH_3_-4′). HRMS for (C_23_H_32_O_3_ [M + H]^+^). Calcd: 341.2475. Found: 341.2462.

(4a*S*)-5-((*E*)-3,4-Dimethoxybenzylidene)-1,1,4a,6-tetramethyl-1,2,3,4,4a,5,8,8a-octahydronaphthalene (**13c**).

Yellow oil, yield: 71.7%. ^1^H NMR (400 MHz, CDCl_3_, δ, ppm): 0.88 (3H, s, CH_3_-14), 0.95 (3H, s, CH_3_-13), 1.02 (3H, s, CH_3_-15), 1.47 (3H, s, CH_3_-12), 3.86 (3H, s, OCH_3_-4′), 3.87 (3H, s, OCH_3_-3′), 5.57 (1H, m, CH-7), 6.24 (1H, s, CH-11), 6.72 (2H d, *J* = 6.0 Hz, CH-2′ y CH-6′), 6.76 (1H, d, *J* = 8.7 Hz, CH-5′). ^13^C NMR (101 MHz, CDCl_3_, δ, ppm): 19.31(C-2), 19.52 (C-15), 21.86 (C-13), 23.11(C-12), 25.36 (C-6), 32.56 (C-14), 33.90 (C-4), 38.00 (C-1), 38.66 (C-10), 42.40 (C-3), 48.28 (C-5), 55.84 (OCH_3_-4′), 55.85 (OCH_3_-3′), 110.48 (C-5′), 112.49 (C-2′), 119.51 (C-11), 121.73 (C-6′), 128.70 (C-7), 131.64 (C-8), 133.42 (C-1′), 147.33 (C-3′), 148.13 (C-4′), 151.03 (C-9). HRMS for (C_23_H_32_O_3_ [M + H]^+^). Calcd: 341.2475. Found: 341.2473.

#### 3.2.6. The General Procedure for the Synthesis of Cyclic Compounds **14a**–**d**

The procedure used by Domingo et al. [45] was followed with some modifications. Iodine (0.37 mmol) was added to a solution of the alcohol **10a**–**d** (0.73 mmol) in 7.25 mL of benzene. The mixture was left to reflux, and the reaction was monitored by TLC. When it was found that no starting product was present, the mixture was diluted with ethylic ether (30 mL), and the organic phase was washed with saturated sodium thiosulfate solution and brine, and then dried and concentrated to obtain a crude, which underwent silica gel chromatography to obtain the product.

(6a*R*,11b*S*)-8,10-Dimethoxy-4,4,6a,11b-tetramethyl-2,3,4,4a,5,6,6a,11b-octahydro-1*H*-benzo[*a*]fluorene (**14a**).

Yellow oil, yield: 54.6%. ^1^H NMR (400 MHz, CDCl_3_, δ, ppm): 0.84 (3H, s, CH_3_-14), 0.93 (3H, s, CH_3_-13), 1.23 (3H, s, CH_3_-15), 1.37 (3H, s, CH_3_-12), 2.15 (2H, dt, *J* = 12.6, 3.0 Hz, CH_2_-7), 3.82 (3H, s, OCH_3_-4′), 3.85 (3H, s, OCH_3_-2′), 6.31 (1H, s, CH-11), 6.34 (1H, d, *J* = 2.0 Hz, CH-3′), 6.45 (1H, d, *J* = 2.0 Hz, CH-5′). ^13^C NMR (101 MHz, CDCl_3_, δ, ppm): 18.64 (C-2), 19.35 (C-6), 19.53 (C-15), 21.62 (C-13), 24.92 (C-12), 33.59 (C-14), 33.73 (C-4), 38.19 (C-1), 38.80 (C-7), 39.22 (C-10), 42.35 (C-3), 51.81 (C-8), 55.40 (OCH_3_-2′), 55.64 (OCH_3_-4′), 57.07 (C-5), 96.38 (C-3′), 99.18 (C-5′), 113.01 (C-11), 123.28 (C-1′), 153.04 (C-2′), 157.60 (C-6′), 158.83 (C-4′), 164.42 (C-9). HRMS for (C_23_H_32_O_3_ [M + H]^+^). Calcd: 341.2475. Found: 341.2471

(6a*R*,11b*S*)-8,9-Dimethoxy-4,4,6a,11b-tetramethyl-2,3,4,4a,5,6,6a,11b-octahydro-1*H*-benzo[*a*]fluorene (**14c**).

Yellow oil, yield: 80%. ^1^H NMR (400 MHz, CDCl_3_, δ, ppm): 0.85 (3H, s, CH_3_-14), 0.94 (3H, s, CH_3_-13), 1.24 (3H, s, CH_3_-15), 1.37 (3H, s, CH_3_-12), 3.88 (3H, s, OCH_3_-3′), 3.89 (3H, s, OCH_3_-4′), 6.15 (1H, s, CH-11), 6.81 (1H, s, CH-5′), 6.84 (1H, s, CH-2′). ^13^C NMR (101 MHz, CDCl_3_, δ, ppm): δ18.65 (C-2), 19.37 (C-15), 19.43 (C-6), 21.66 (C-13), 24.92 (C-12), 33.62 (C-14), 33.78 (C-4), 38.29 (C-1), 39.08 (C-7), 39.29 (C-10), 42.35 (C-3), 51.34 (C-8), 56.15 (OCH_3_-4′), 56.40 (OCH_3_-3′), 57.03 (C-5), 104.45 (C-5′), 105.61 (C-2′), 117.10 (C-11), 134.65 (C-1′), 146.55 (C-4′), 147.09 (C-6′), 147.97 (C-3′), 166.98 (C-9). HRMS for (C_23_H_32_O_3_ [M + H]^+^). Calcd: 341.2475. Found: 341.2474.

(6a*R*,11b*S*)-7,9-Dimethoxy-4,4,6a,11b-tetramethyl-2,3,4,4a,5,6,6a,11b-octahydro-1*H*-benzo[*a*]fluorene (**14d**).

Brown oil, yield: 52%. ^1^H NMR (400 MHz, CDCl_3_, δ, ppm): 0.85 (3H, s, CH_3_-14), 0.90 (3H, s, CH_3_-13), 1.26 (3H, s, CH_3_-15), 1.46 (3H, s, CH_3_-12), 2.53 (1H, dt, *J* = 12.6, 3.0 Hz, CH_2_-7), 3.81 (6H, s, OCH_3_-3′ + OCH_3_-5′), 6.12 (1H, s, CH-11), 6.25 (1H, d, *J* = 2.1 Hz, CH-4′), 6.47 (1H, d, *J* = 2.1 Hz, CH-2′).^13^C NMR (101 MHz, CDCl_3_, δ, ppm): 18.60 (C-2), 18.99 (C-6), 19.33 (C-15), 21.40 (C-12), 21.63 (C-13), 33.57 (C-14), 33.74 (C-4), 37.17 (C-1), 38.34 (C-7), 39.35 (C-10), 42.26 (C-3), 51.72 (C-8), 55.08*, 55.47*, 56.86 (C-5), 95.10 (C-4′), 97.83 (C-2′), 117.44 (C-11), 132.79 (C-6′), 144.34 (C-1′), 155.37 (OCH_3_-5′), 160.20 (OCH_3_-3′), 170.34 (C-9). * Interchangeable signals: (OCH_3_-3′), (OCH_3_-5′). HRMS for (C_23_H_32_O_3_ [M + H]^+^). Calcd: 341.2475. Found: 341.2475.

### 3.3. Biological Assays

#### 3.3.1. Cultured Cell Lines

The American Type Culture Collection (Rockville, MD, USA) supplied the following experimental cell lines: MCF-7, a human breast cancer cell line, and MCF-10A, a human epithelial breast cell line (ATCC N° CRL-10317). Both cell lines were grown in DMEM-F12 medium containing 10% FCS, 100 U/mL penicillin, 100 μg/mL streptomycin, and 1 mM glutamine. The cells were seeded at a concentration of 100 μL per well at a plating density of 3 × 10^3^ cells/well and incubated for 24 h at 37 °C under 5% humidified CO_2_ to enable attachment. The cells were then treated with varying concentrations of synthesized compounds for 72 h under the same conditions. The compound stock solution was prepared in DMSO, and the final concentration of DMSO was kept at 0.1%. Control cultures were treated with only 1% ethanol.

#### 3.3.2. In Vitro Cytotoxicity Screening by Using MTT Assay

Cells from the MCF-7 and MCF-10 lines were seeded into 96-well plates at a density of 1 × 10^4^ per well. After 24 h, the compounds were added at increasing concentrations (500–100–20-4–0.8–0.16–0.032–0 µM), and the cells were incubated at 37 °C in a humidified 5% CO_2_/95% air mixture for 48 h. Following incubation, the cells were washed twice with PBS, and 100 μL of 0.5 mg/mL MTT solution was added to each well. After 2 h of incubation, the MTT solution was removed, and the formazan crystals were dissolved in 50 μL of DMSO per well. Absorbance was then measured at 570 nm using a microplate ELISA reader. IC_50_ values, determined from three independent experiments conducted in triplicate, were calculated using SigmaPlot^®^ software (version 11.0). The selectivity index (SI) of each compound in each cell line was determined by calculating the ratio of the IC_50_ values for MCF-10 and cancer cell lines. Compounds were considered selective if SI values were equal to or greater than 3 [46].

#### 3.3.3. ROS in Cell Lines Exposed to Compounds

Reactive oxygen species (ROS) production was measured using flow cytometry. Cells were treated with compounds (each with respective IC_50_ values) for 24 h, with untreated cells serving as the negative control and cells treated with 1 µM daunorubicin as the positive control. Following the treatment, intracellular ROS levels were visualized after incubation with 2′,7′-dichlorodihydro-fluorescein diacetate (DCFH2-DA) at a final concentration of 10 μM for the last 30 min of the treatment period. After incubation, the cells were washed with PBS, trypsinized, and centrifuged, and the resulting pellet was resuspended in PBS and immediately examined by flow cytometry [47].

#### 3.3.4. Determination of Mitochondrial Membrane Potential (ΔΨmt) by Flow Cytometry

Rhodamine 123 (Rho123), a positively charged dye that reacts to voltage changes and enters the mitochondria, was utilized to detect modifications in the mitochondrial membrane potential. The cells were exposed to specific compounds (IC_50_ values for each compound) for 24 h. Control cells that had not been treated were used as negative control, while cells treated with daunorubicin at a concentration of 1 µM served as the positive control. Following this, the cells were incubated with Rho123 (at a concentration of 1 µM) in darkness for an hour at 37 °C. Afterward, the medium was taken out, and the cells were washed with PBS. The cells were then dissociated using trypsin and collected through centrifugation for 10 min at 1500×*g*. The supernatant was thrown away, and the cell pellets were resuspended in PBS and analyzed using flow cytometry with the FL1 filter. The results are shown as the percentage of Rho123-stained cells.

#### 3.3.5. Determination of Caspases-3/7 Activation

The activity of caspase 3/7 in MCF-7 cells treated with the compounds was determined using a Caspase-Glo-3/7 Assay kit from Promega (UK). This assay is a homogenous and luminescent method that measures the activity of caspase-3 and -7, which are essential for apoptosis. MCF-7 cells (1 × 10^4^ cells per well) were seeded in a 96-well plate and incubated overnight. The cells were then treated for 24 h with the compounds (50 µM) and a blank as a control. After treatment, the Caspase-Glo^®^ 3/7 reagent was prepared according to the manufacturer’s instructions. Specifically, 100 μL of the reagent was added to 100 μL of culture medium containing the treated cells in each well, and the mixture was incubated for 1 h at room temperature in the dark. Luminescence was then measured using a luminometer (Berthold Sirius Single Tube Luminometer). The fold change in caspase activity was calculated by expressing the average luminescence of each treatment as a fold change with respect to the average luminescence of the negative control.

#### 3.3.6. Topoisomerase I/II Activity Assay

The effects of test compounds were analyzed to measure changes in topoisomerase I or II activity.

TOP1 activity was assayed by the relaxation of supercoiled DNA (pEGFP-N1) as the manufacturer’s instructions indicated. Briefly, human recombinant TOP1 and buffer assay were obtained from Sigma-Aldrich (St. Louis, MO, USA). An amount of 1U of human topoisomerase I, 200 ng of plasmid DNA, and the indicated concentrations (20 μM) of compounds were incubated for 30 min at 37 °C in 2 μL of Topo I buffer (500 mM Tris·HCl, pH 7.5, 1 M KCl, 10 mM dithiothreitol, 100 mM EDTA, 50 μg/mL acetylated bovine serum albumin (BSA)) in a total volume of 20 μL. The reaction was stopped by the addition of 5 μL of 5× loading dye. Samples were electrophoresed on a 0.8% agarose gel without ethidium bromide for 2–3 hours at 5 to 10 V/cm. The gel was stained with ethidium bromide and photographed with a UV transilluminator.

Topoisomerase II-mediated DNA cleavage was assessed using a plasmid linearization assay adapted from previously described methods [48,49]. Supercoiled plasmid DNA (200 ng final concentration) was incubated with purified human topoisomerase II (10 U) in the presence or absence of test agents or a positive control. Reactions were carried out in a total volume of 20 μL, composed of 2 μL of 10× topoisomerase II reaction buffer, an appropriate volume of compound or control, and nuclease-free water. ATP (final concentration adjusted from a 20 mM stock) was freshly added to the reaction buffer immediately prior to use. Reactions were initiated by the addition of topoisomerase II and incubated at 37 °C for 30 min. To terminate the reactions, 2 μL of 10% SDS was added directly to the tubes while still in the 37 °C water bath, followed by the addition of 1.5 μL of 250 mM tetrasodium EDTA (pH 8.0) and 2 μL of proteinase K solution (0.8 mg/mL). Samples were then incubated at 30 °C for 1–2 h to ensure the complete digestion of protein–DNA adducts. Thereafter, 2 μL of 5× loading dye was added to each sample, and the mixtures were subjected to electrophoresis on a 0.8% agarose gel at 5 V/cm for 2–4 h. After electrophoresis, gels were stained with ethidium bromide (1 μg/mL) for 30–60 min and destained in water for 5–10 min. The gel was stained with ethidium bromide and photo-graphed with a UV transilluminator.

### 3.4. In Silico Studies

#### 3.4.1. Docking

The Induced Fit Docking module within the Schrödinger suite was employed to carry out molecular docking studies. The TOP2, DNA, and etoposide complex (PDB ID: 3QX3) was obtained using the Protein Preparation Wizard module. The protein was preprocessed at pH 7.0 ± 2.0, and all water molecules, ions, and other compounds were removed from the crystal structure. The internal hydrogen bonds of the complex were refined using the “optimize” option, followed by final minimization using the OPLS3e force field.

Meanwhile, the compounds underwent minimization using the LigPrep module at pH = 7.0 ± 2.0, with the chirality of each stereocenter being retained. The OPLS3e force field was used to minimize the compounds, and the chirality of each atom was manually verified to prevent configuration inversions. The final docking of the molecules took place in the Induced Fit Docking module, with the etoposide site serving as the grid center, and a 20 Å cubic box was defined. The analysis utilized the OPLS3e force field and an XP precision level, and the results were manually processed using the suite’s visualization module.

#### 3.4.2. Molecular Dynamics Simulation

Simulation research was carried out with the aid of the Desmond component. To achieve this, the optimal docking configuration of the complex and compound was utilized. The simulation parameters were as follows using the NPT ensemble: 100 ns, 1000 frames, 1 atm of pressure, and 300 K. The final simulation stance was manually extracted and assessed using the visualizer included in the suite.

## 4. Conclusions

Continuing with our program for new antitumoral sesquiterpene derivatives bioinspired by natural products, in this study, we present the design and synthesis of seventeen novel aryl-sesquiterpenes that were prepared using (-)-drimenol as a starting material. The cytotoxic effect of MCF-7 cells showed that the substitution of the C-11 methylene linker and dimethoxy groups in the aryl fragments are pivotal factors for biological activity. **14c** was one of the most potent antitumoral merosesquiterpenes among all derivatives with an IC_50_ value of 9.0 μM and higher selectivity than the reference drug daunorubicin. Furthermore, the results indicate that the toxicity of **14c** in MCF-7 cells could be attributed to its capacity to elevate the intracellular levels of ROS, activate caspases-3/7, and selectively inhibit TOP2, ultimately suggesting an apoptosis-mediated cell death. In silico studies explained the selectivity to TOP1/2 for **14c**, which was shown by an intercalative binding mode between the nitrogenous bases of the DNA-TOP2 complex, with this being very stable at 100 ns. These data suggest that compound **14c** would act as a topoisomerase 2 poison. Finally, the predicted pharmacokinetic profile of **14c** indicates that it would be an effective candidate for oral administration. These results imply that **14c** is a potential candidate for the development of novel agents targeting breast cancer.

## Figures and Tables

**Table 1:** In vitro cytotoxicity of final products on MCF-7 and MCF-10 cells and selectivity index (SI) values.

Compound	IC_50_ (µM) ^[a]^		Selectivity Index (SI) ^[b]^
	MCF-7	MCF-10	
**10a**	74.2 ± 7.0	53.2 ± 10.0	0.7
**10b**	130.7 ± 26.6	10.1 ± 3.0	0.1
**10c**	25.1 ± 2.2	247.9 ± 42.3	9.9
**10d**	125.6 ± 11.5	12.3 ± 3.1	0.1
**11a**	75.2 ± 11.3	76.7 ± 3.0	1.0
**11b**	132.7 ± 14.9	54.4 ± 3.5	0.4
**11c**	13.5 ± 1.8	87.7 ± 17.8	6.5
**11d**	187.2 ± 9.0	164.6 ± 12.5	0.9
**12a**	56.2 ± 7.3	45.4 ± 4.8	0.8
**12b**	88.1 ± 19.9	72.7 ± 4.3	0.8
**12c**	30.1 ± 6.6	130.8 ± 29.3	4.3
**12d**	111.8 ± 25.4	107.4 ± 15.4	1.0
**13a**	48.9 ± 8.0	70.9 ± 15.1	1.4
**13c**	22.5 ± 2.6	134.9 ± 26.6	6.0
**14a**	8.4 ± 1.8	52.4 ± 3.5	6.3
**14c**	9.0 ± 2.8	393.0 ± 47.1	44
**14d**	119.7 ± 29.8	31.4 ± 4.6	0.3
**Daunorubicin**	0.33 ± 0.02 ^[c]^	9.32 ± 1.01 ^[c]^	28

^[a]^ The IC_50_ values were determined in three separate experiments, with each experiment conducted in triplicate. The results are expressed as the IC_50_ ± SD values. ^[b]^ Selectivity index (SI) value = IC_50_ on MCF-10 cells/IC_50_ on MCF-7 cells. ^[c]^ IC_50_ values from reference [33].

**Table 2:** Molecular properties, ADME parameters, and toxicity predicted for **14c**.

Compound	MW (Da)	HBA	HBD	cLogP	TPSA (Å^2^)	NRB	hERG	CYP Substrate	Pgp Inhibition
Desirable value	≤500	≤10	≤5	≤5	≤140	≤10	M or L	-	Yes
**14c**	340.5	2	0	5.49	18.46	2	M	3A4	Yes

The predicted values were obtained from the SwissADME website (http://www.swissadme.ch/index.php, accessed on 15 March 2025). hERG = in vitro human ether-a-go-go-related gene channel inhibition. M, L = medium or low risk. hERG inhibition, CYP, and Pgp estimations were calculated using PreAdmet (https://preadmet.webservice.bmdrc.org/toxicity/, accessed on 15 March 2025).

## Data Availability

The data presented in this study are available in the Supplementary Material.

## Supplementary Materials

The following supporting information can be downloaded at https://www.mdpi.com/article/10.3390/ijms26104539/s1.
