# Supplementary material for: New Dimethoxyaryl-Sesquiterpene Derivatives with Cytotoxic Activity Against MCF-7 Breast Cancer Cells: From Synthesis to Topoisomerase I/II Inhibition and Cell Death Mechanism Studies
Source: Int J Mol Sci. 2025 May 9;26(10):4539. doi: 10.3390/ijms26104539 (PMC12111648; doi:10.3390/ijms26104539)
Supplement: Supplementary file 1 [file ijms-26-04539-s001.zip › ijms-3609690-supplementary.pdf]

New dimethoxyaryl-sesquiterpene derivatives with cytotoxic activity against MCF-7 breast cancer cells: From Synthesis, Topoisomerase I/II Inhibition to Cell Death Mechanism Studies

Ileana Araque [1, 6&], Rut Vergara [2&], Jaime Mella [3,4], Pablo Aránguiz [5], Luis Espinoza [6], Cristian O. Salas [7], Alejandro F. Barrero [8], José Quílez del Moral [8], Joan Villena [2*] and Mauricio A. Cuellar [1,4*]

[1] Facultad de Farmacia, Escuela de Química y Farmacia, Universidad de Valparaíso, Av. Gran Bretaña 1093, Valparaíso 2340000, Chile; ileana.araque@postgrado.uv.cl (I.A.).
[2] Centro Interdisciplinario de Investigación Biomédica e Ingeniería para la Salud (MEDING), Escuela de Medicina, Facultad de Medicina, Universidad de Valparaíso, Viña del Mar 2520000, Chile; rut.vergara@postgrado.uv.cl (R.V.); juan.villena@uv.cl (J.V.)
[3] Instituto de Química y Bioquímica, Facultad de Ciencias, Universidad de Valparaíso, Valparaíso 2340000, Chile; jaime.mella@uv.cl
[4] Centro de Investigación, Desarrollo e Innovación de Productos Bioactivos (CINBIO), Universidad de Valparaíso, Valparaíso 2340000, Chile; mauricio.cuellar@uv.cl
[5] Escuela de Ciencias de la Salud. Carrera de Química y Farmacia, Universidad Viña del Mar, Viña del Mar Chile 2520000, Chile; pablo.aranguiz@uvm.cl
[6] Departamento de Química, Universidad Técnica Federico Santa María, Avenida España 1680, Valparaíso 2340000, Chile; luis.espinozac@usm.cl
[7] Departamento de Química Orgánica, Facultad de Química y de Farmacia, Pontificia Universidad Católica de Chile, Avenida Vicuña Mackenna 4860, Macul, Santiago de Chile 702843, Chile; cosalas@uc.cl
[8] Departamento de Química Orgánica, Instituto de Biotecnología, Universidad de Granada, Granada 18071, Spain; jfquilez@ugr.es, afbarre@ugr.es
[#] These authors contributed equally to this work
* Correspondence: juan.villena@uv.cl (J.V.) and mauricio.cuellar@uv.cl (M.A.C.); Tel.: +56-32-2508439Corresponding authors

Mauricio A. Cuellar
Email: mauricio.cuellar@v.cl

Supplementary Information

Index

^{1}H and ^{13}C NMR spectra and HRMS of selected compounds……………..…………………………….....p. 2-36

Figure S1. Best docking pose for compound **13c** in the DNA-Topoisomerase II complex (PDB ID: 3QX3). ……………………………………………………………………………………………………....p. 37

Figure S2. 2D interaction diagram for compound **13c** in the DNA-Topoisomerase II complex (PDB ID: 3QX3). ……………………………………………………………………………........................p. 37

Figure S3. Far view of docking pose for compound **14c** in the DNA-Topoisomerase I complex (PDB ID: 1K4T)…………………………………………………………………………………….……...p. 38

Figure S4. Close view of the docking pose for compound **14c** in the DNA-Topoisomerase I complex (PDB ID: 1K4T)……………………………………………………………………………...…...p. 38

Drimenal (8)

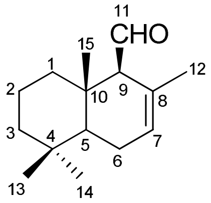

^{1}H NMR Drimenal

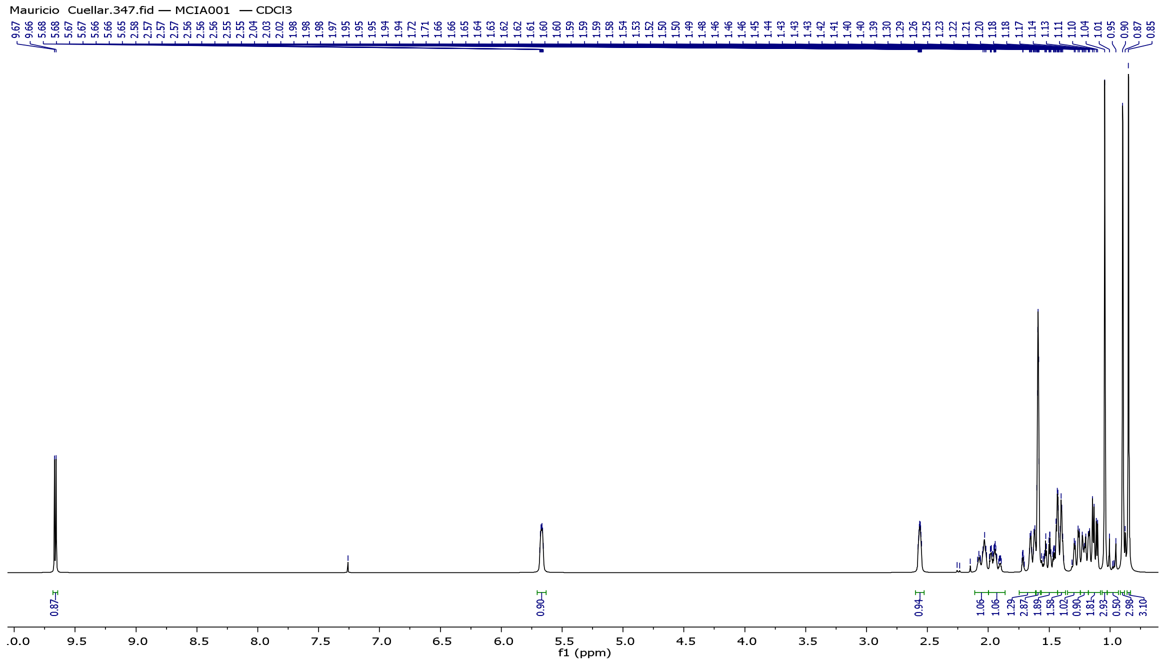

^{13}C NMR Drimenal

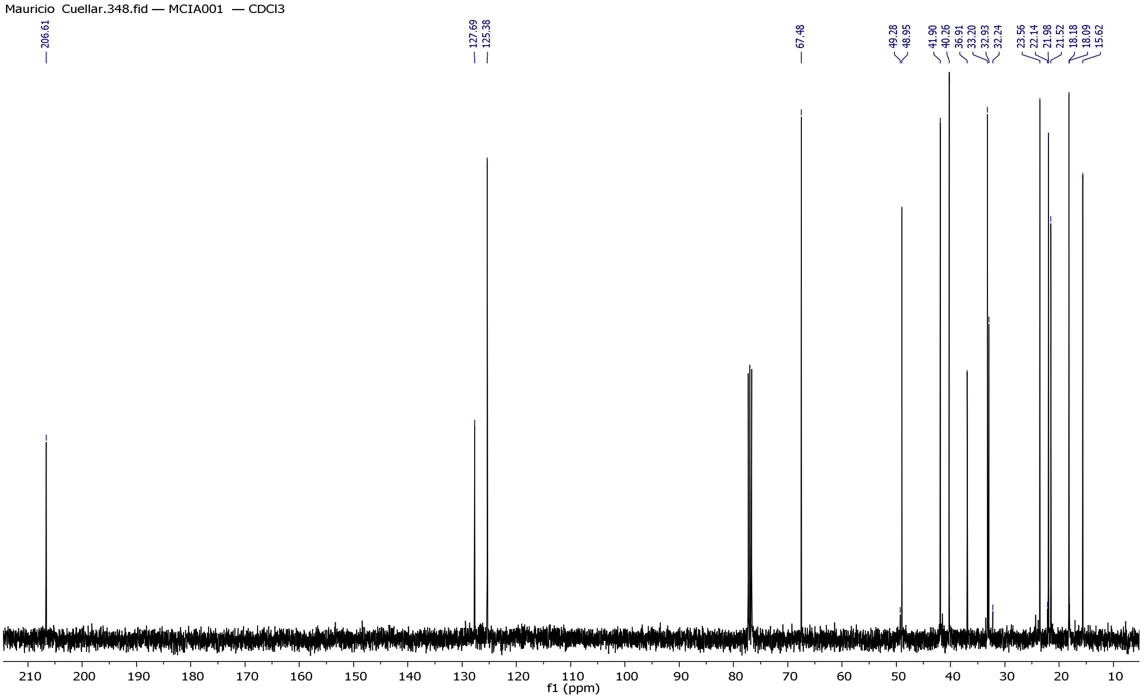

Alcohol compounds 10a-d

(1*S*)-(2,4-Dimethoxyphenyl) ((1S,8aS)-2,5,5, 8a-tetramethyl-1,4, 4a,5,6,7,8, 8a-octahydronaphthalen-1-yl) methanol (**10a**)

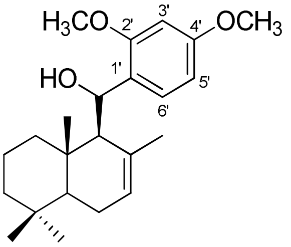

^{1}H NMR 10a

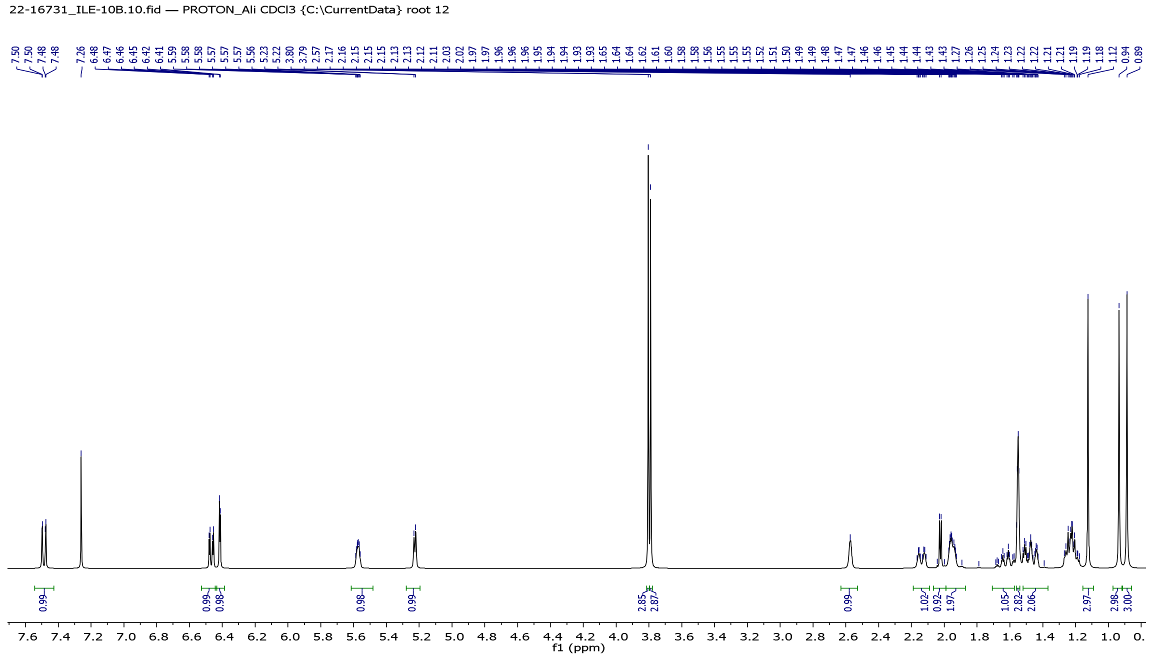

^{13}C NMR 10a

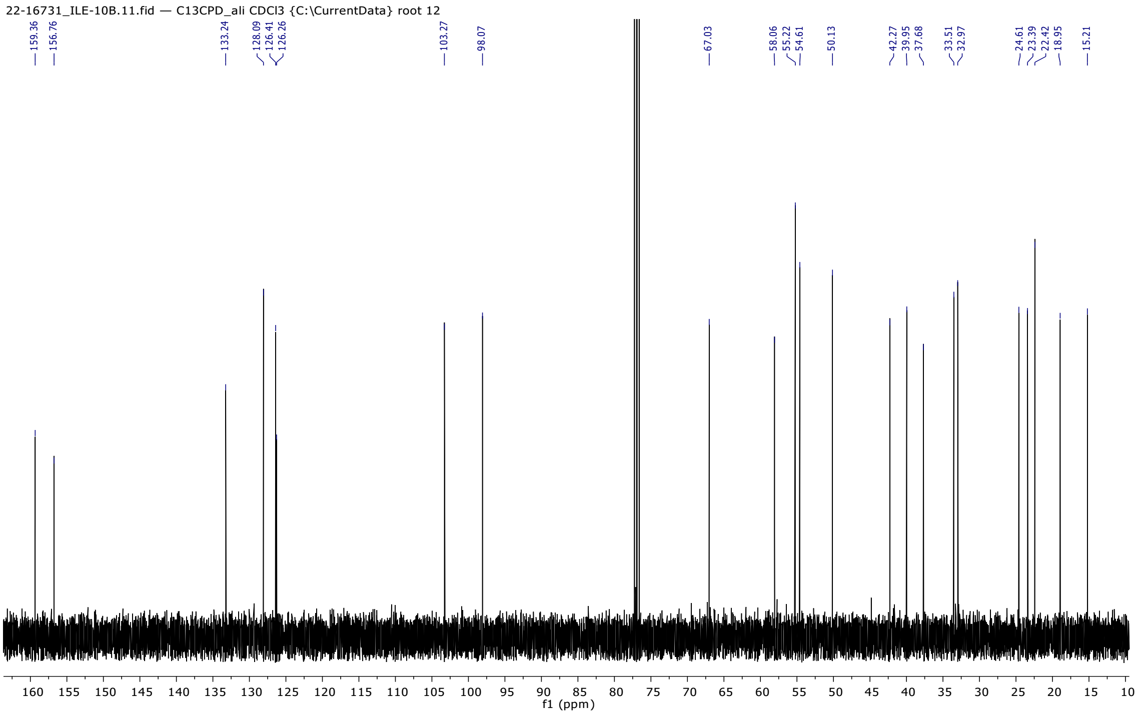

HRMS for 10a

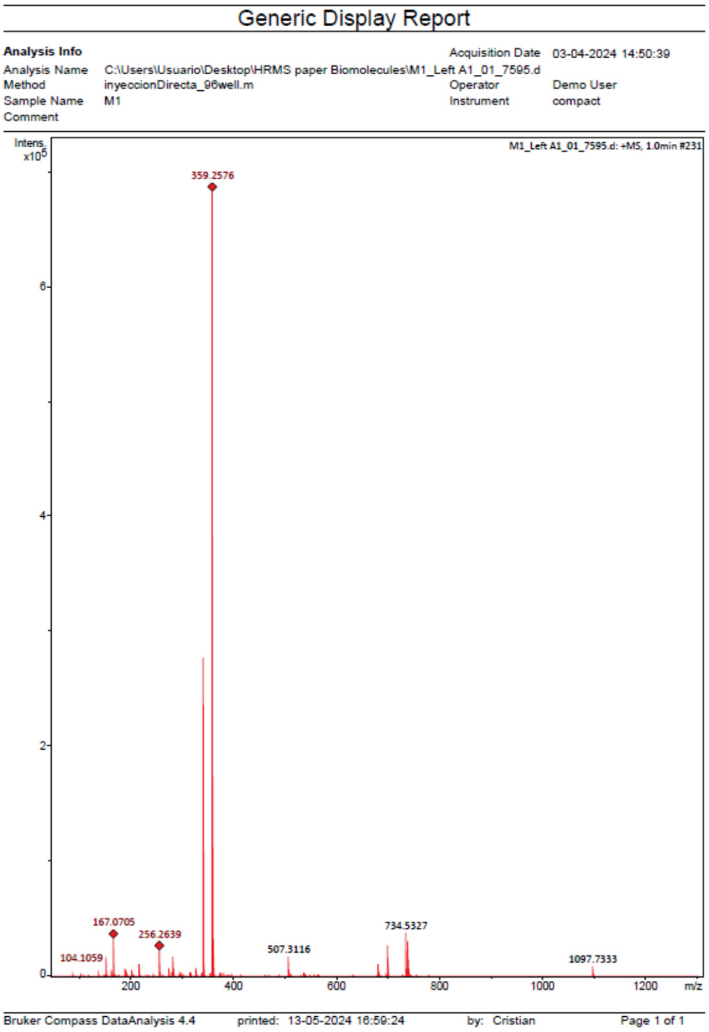

(1*S*)-(2,5-Dimethoxyphenyl)((1S,8aS)-2,5,5,8a-tetramethyl-1,4,4a,5,6,7,8,8a-octahydronaphthalen-1-yl)methanol (**10b**)

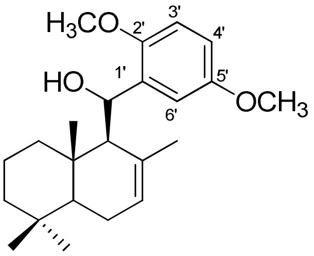

^{1}H NMR 10b

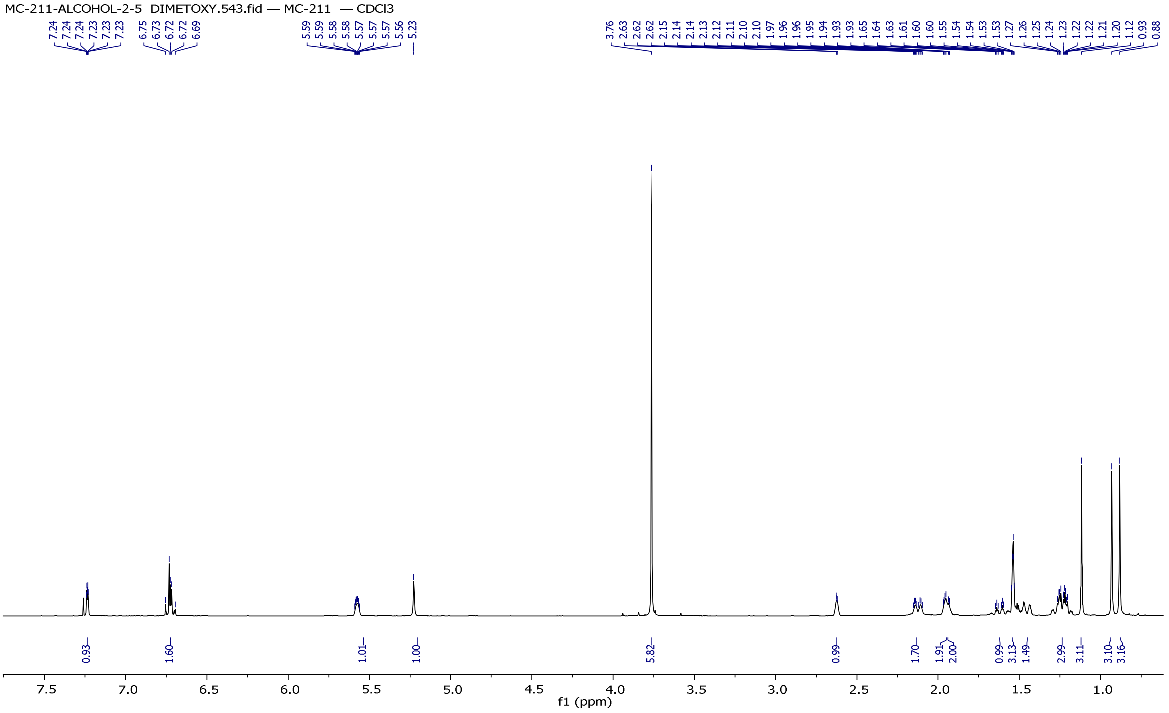

^{13}C NMR 10b

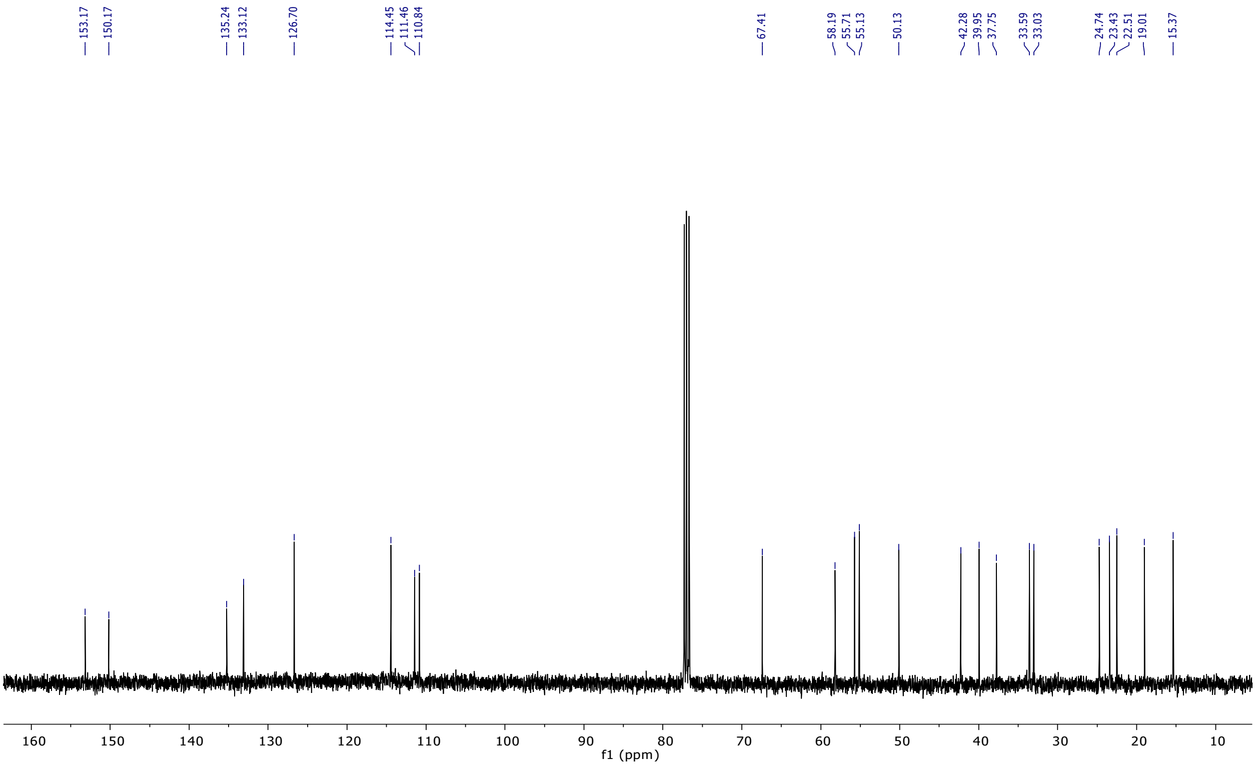

HRMS for 10b

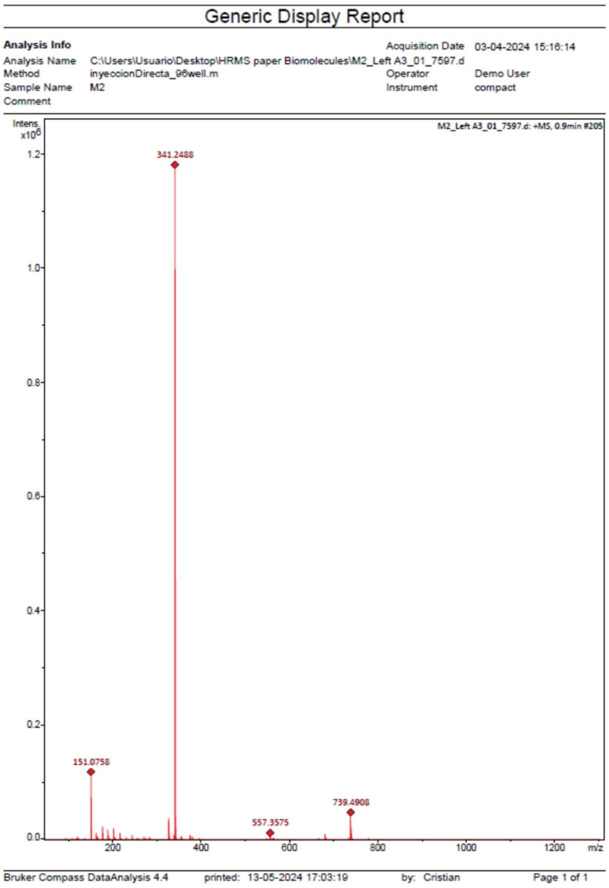

(1*S*)-(3,4-Dimethoxyphenyl) ((1S,8aS)-2,5,5, 8a-tetramethyl-1,4, 4a,5,6,7,8,8a-octahydronaphthalen-1-yl) methanol (**10c**)

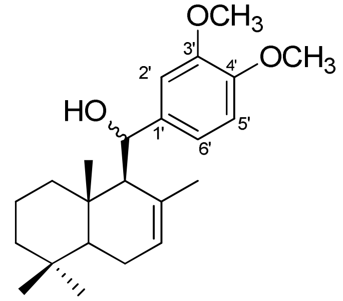

^{1}H NMR 10c

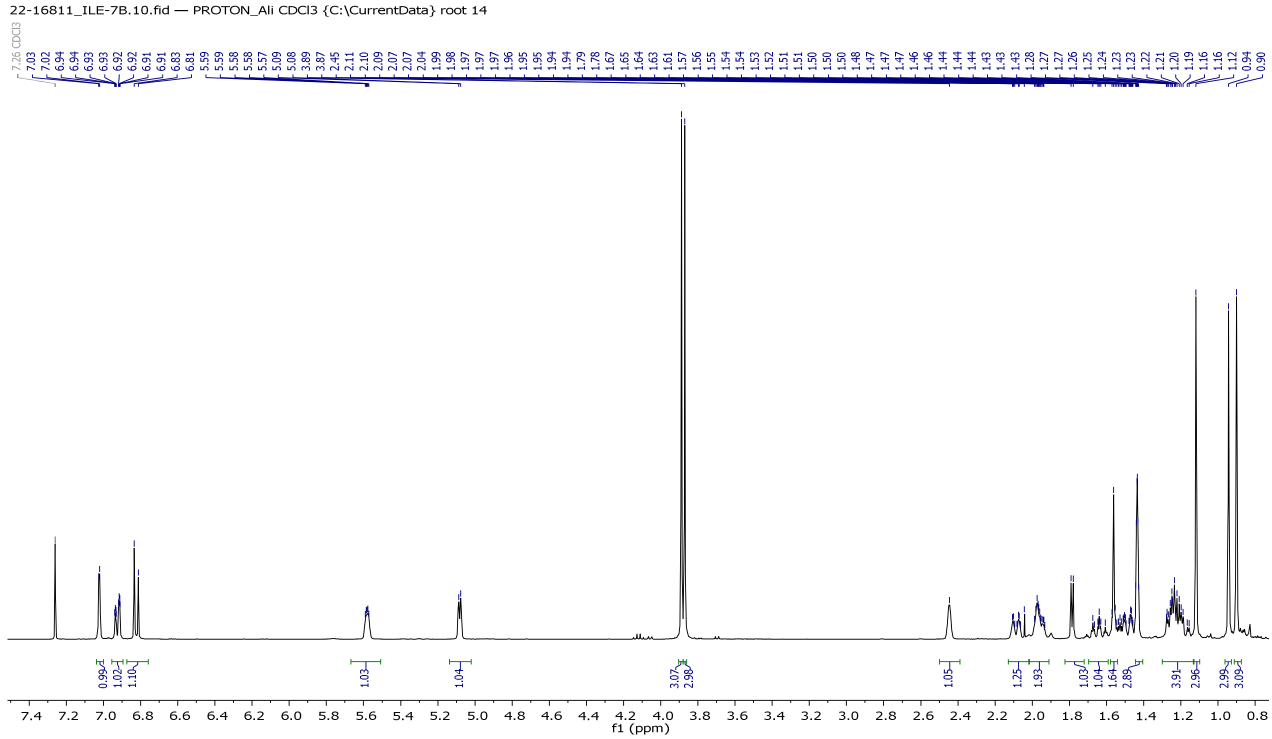

^{13}C NMR 10c

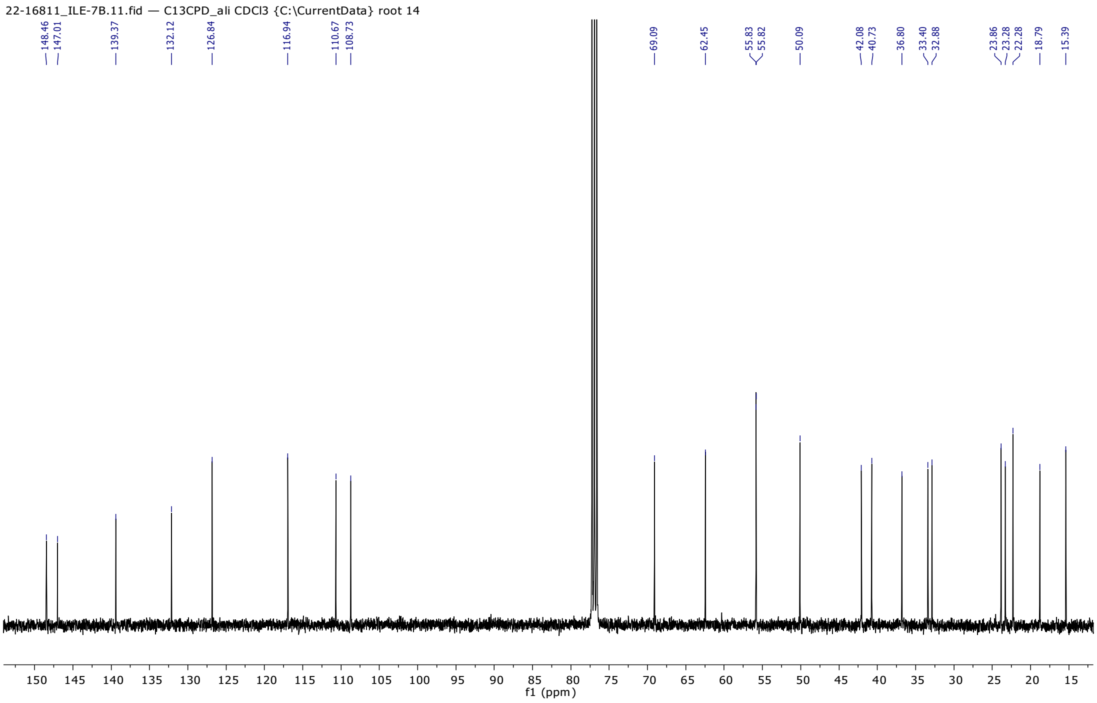

HRMS for 10c

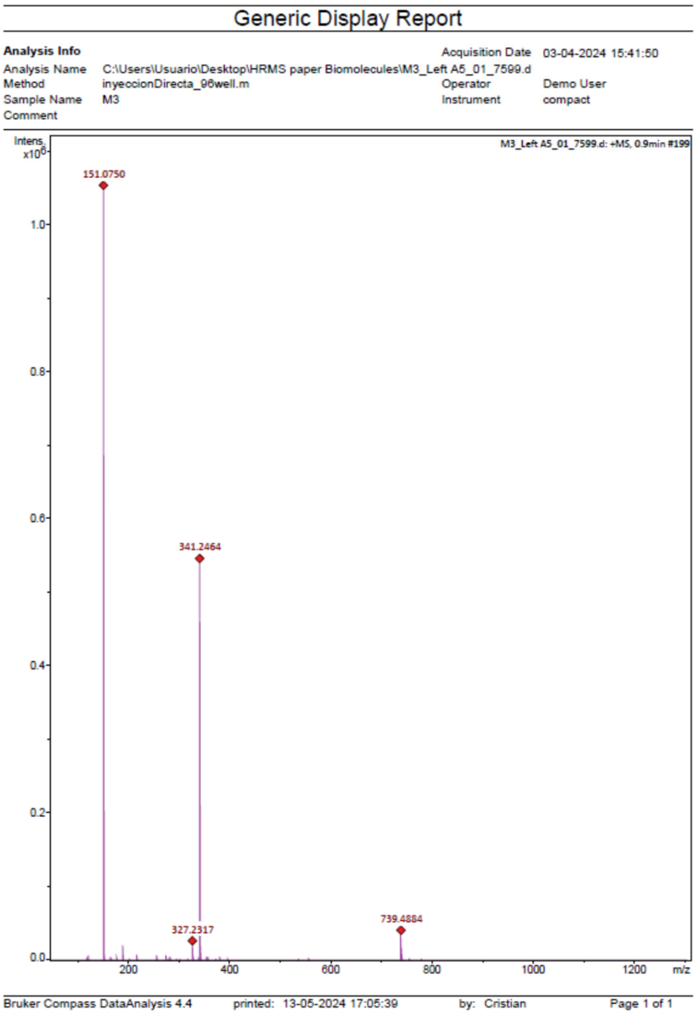

(1*S*)-(3,5-Dimethoxyphenyl)((1S,8aS)-2,5,5,8a-tetramethyl-1,4,4a,5,6,7,8,8a-octahydronaphthalen-1-yl)methanol (**10d**)

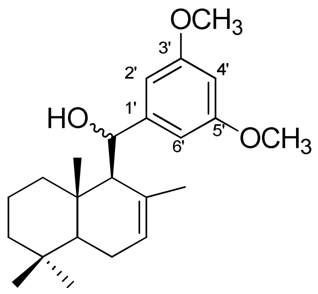

^{1}H NMR 10d

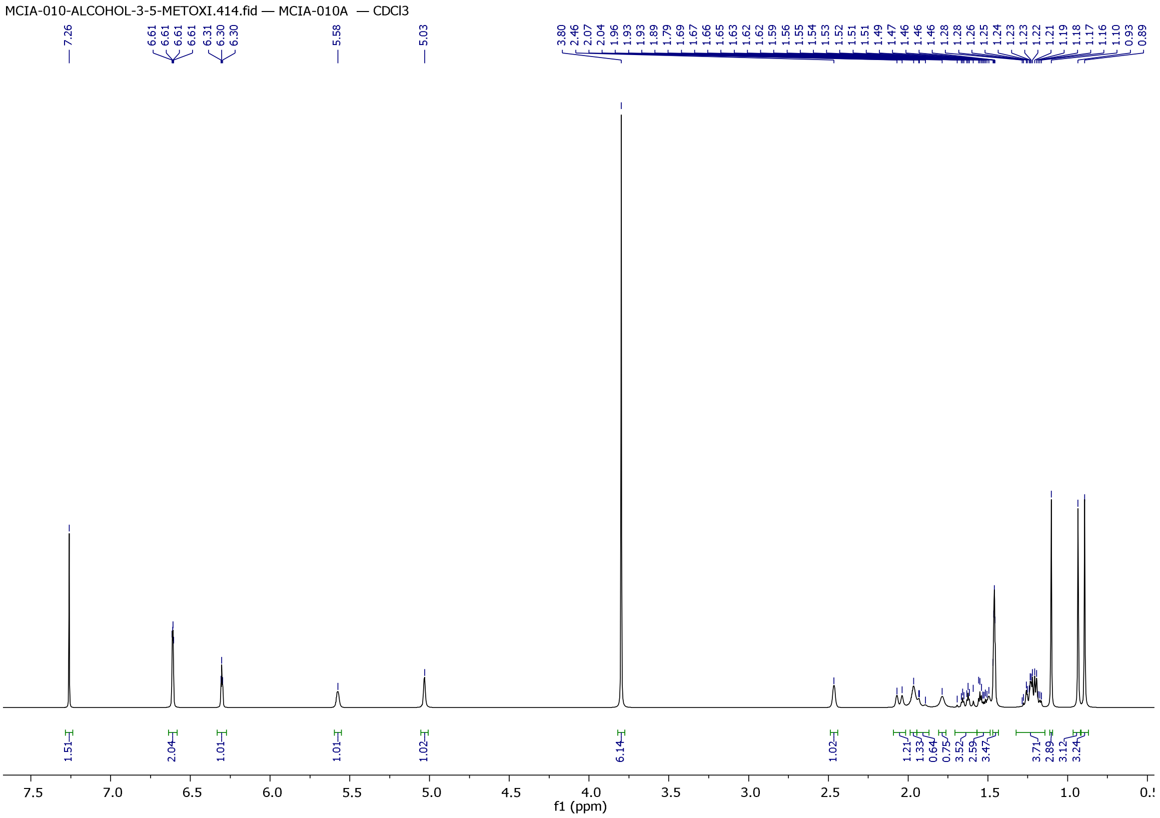

^{13}C NMR 10d

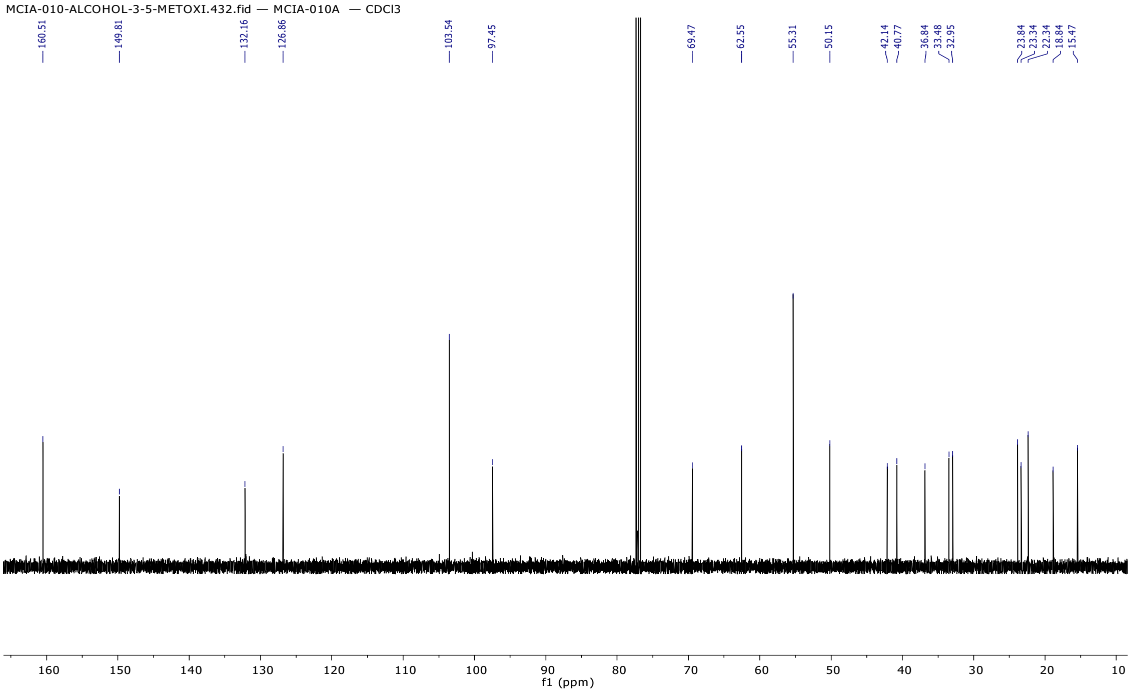

HRMS for 10d

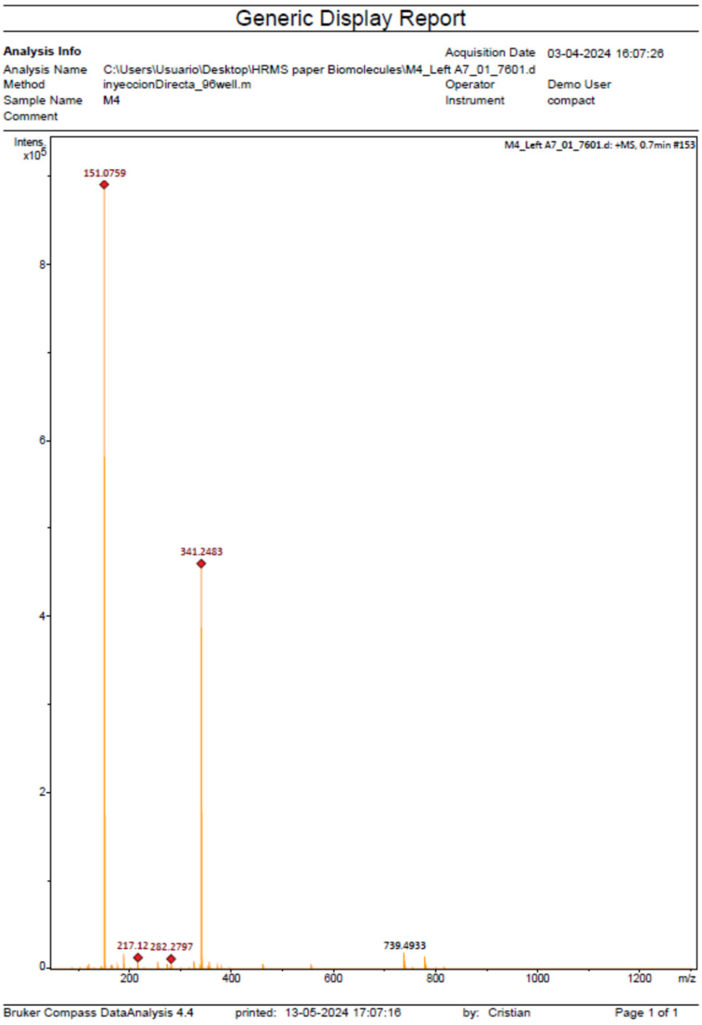

Reduced compounds 11a-d.

(4a*S*,5*S*)-5-(2,4-Dimethoxybenzyl)-1,1,4a,6-tetramethyl-1,2,3,4,4a,5,8,8a-octahydronaphthalene (**11a**)

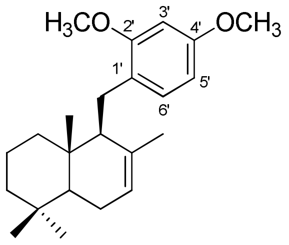

^{1}H NMR 11a

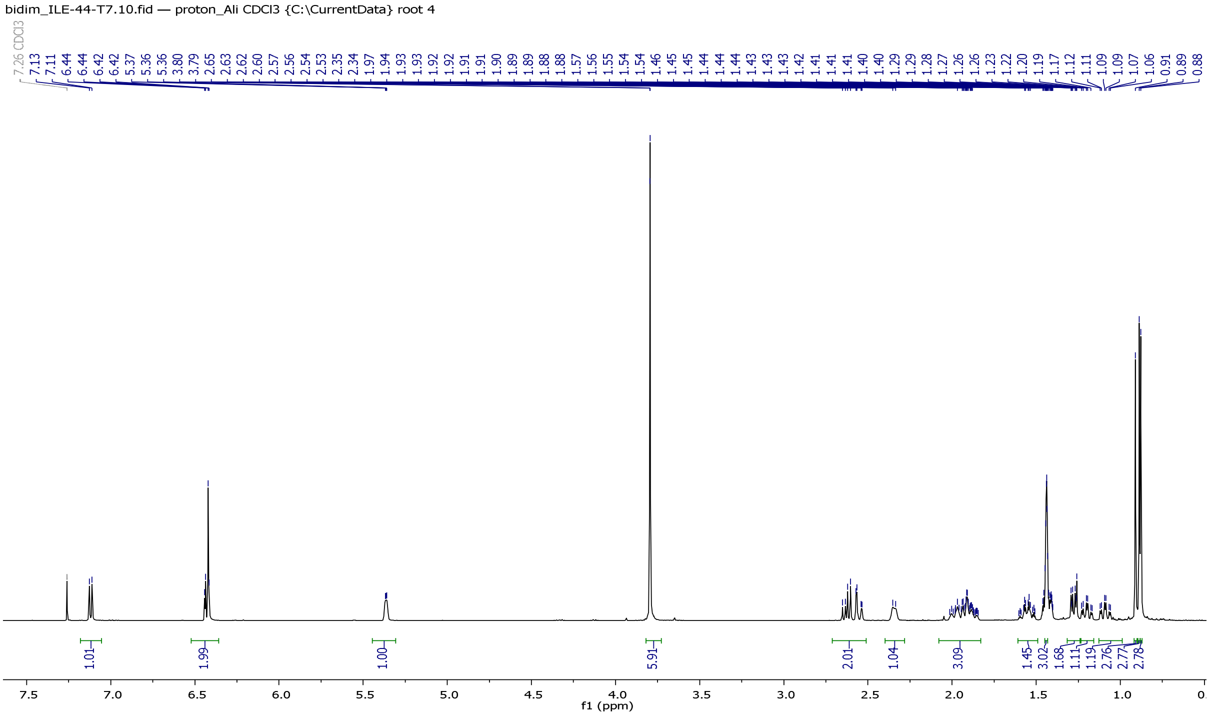

^{13}C NMR 11a

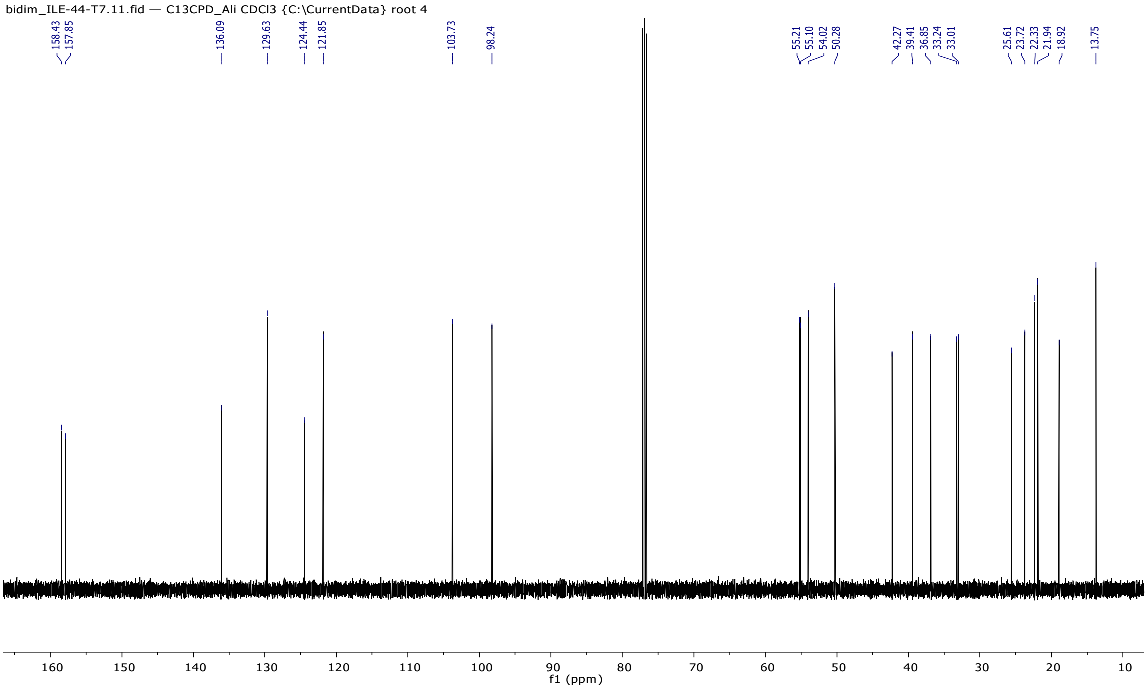

HRMS for 11a

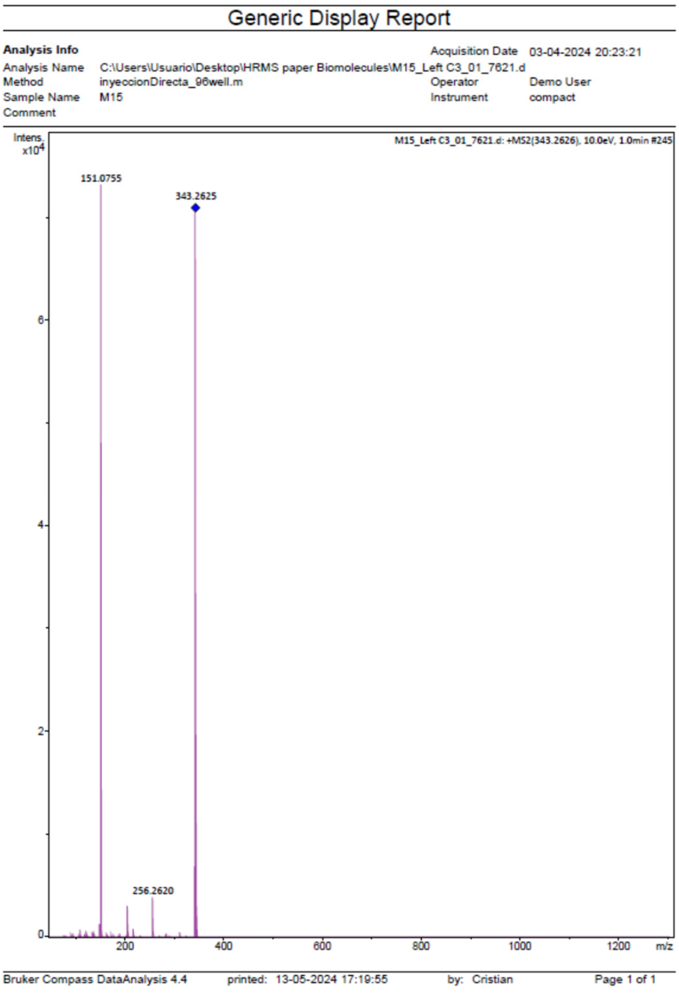

(4a*S*,5*S*)-5-(2,5-Dimethoxybenzyl)-1,1,4a,6-tetramethyl-1,2,3,4,4a,5,8,8a-octahydronaphthalene (**11b**)

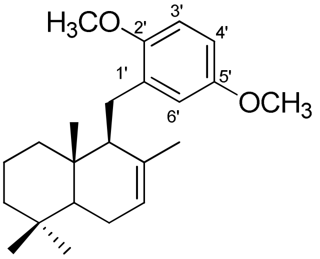

^{1}H NMR 11b

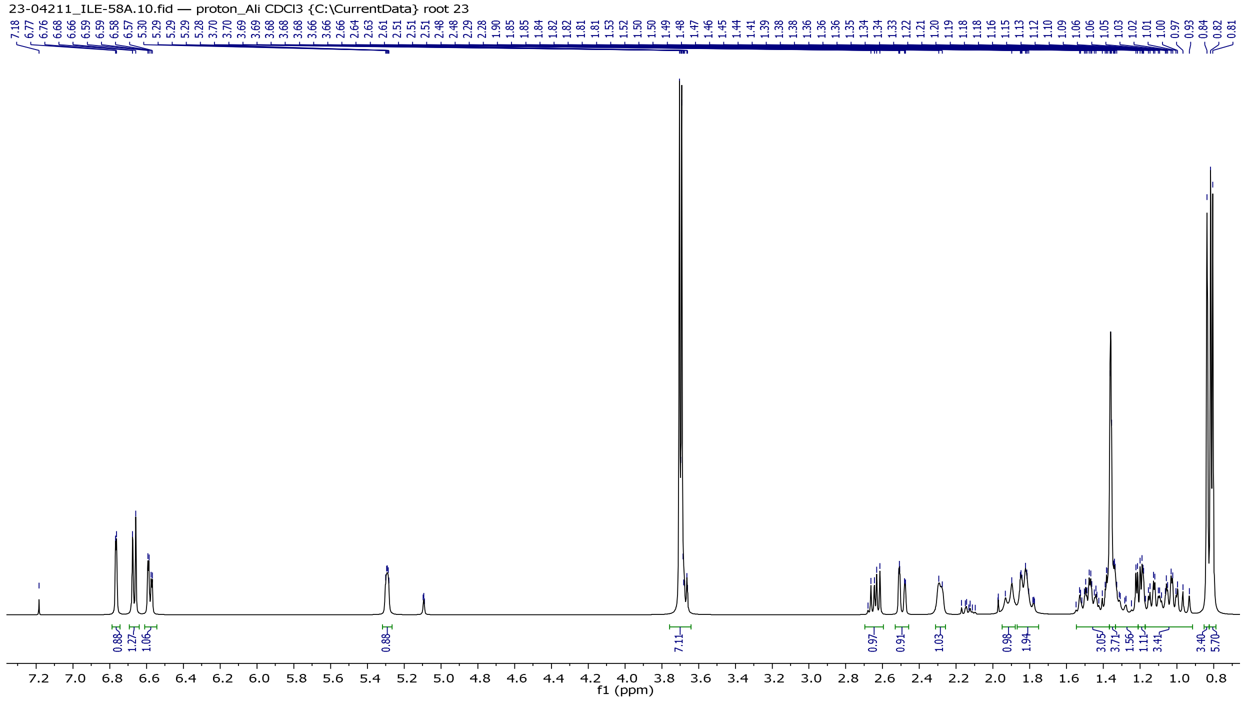

^{13}C NMR 11b

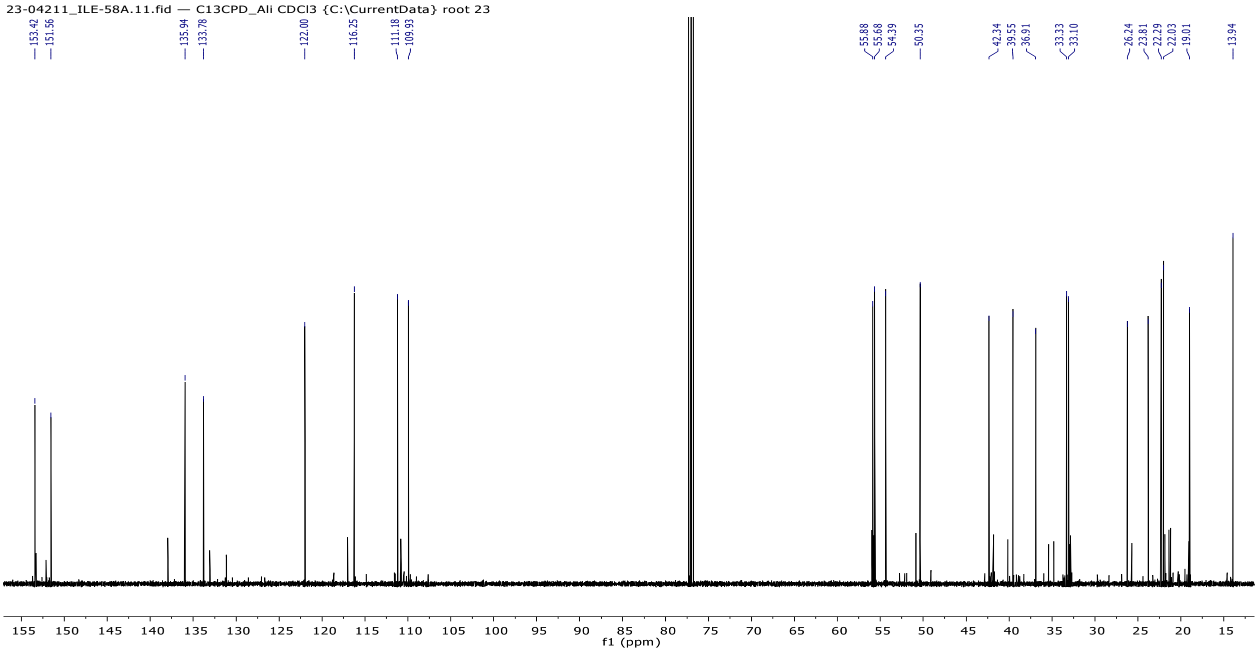

HRMS for 11b

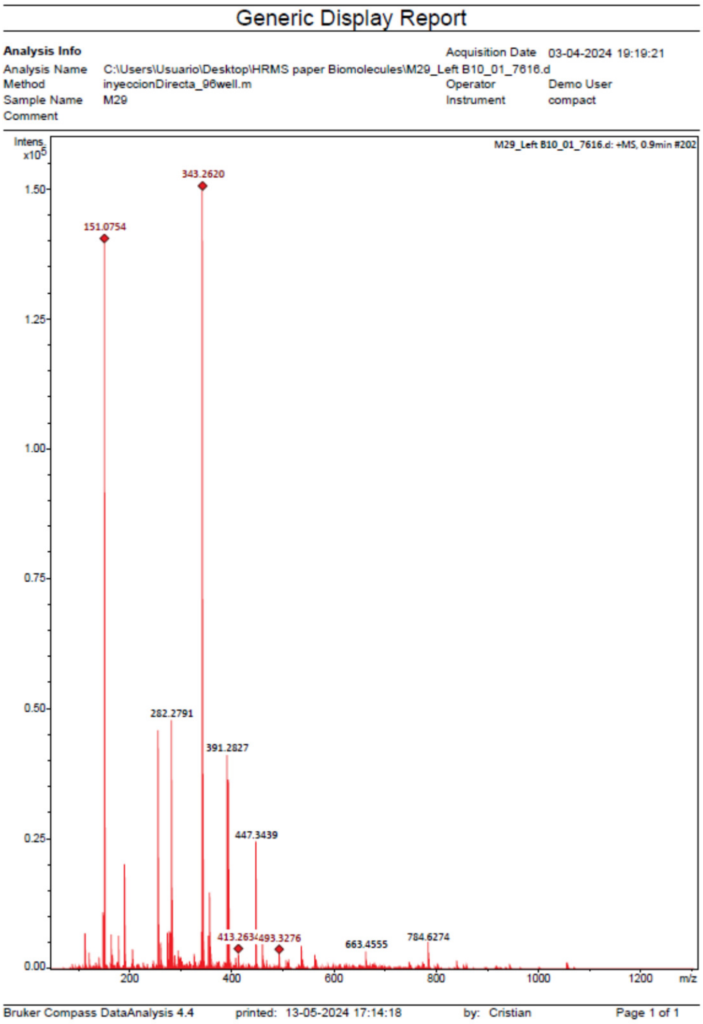

(4a*S*,5*S*)-5-(3,4-Dimethoxybenzyl)-1,1,4a,6-tetramethyl-1,2,3,4,4a,5,8,8a-octahydronaphthalenene (**11c**)

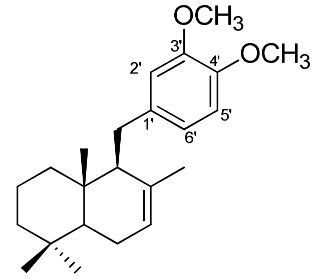

^{1}H NMR 11c

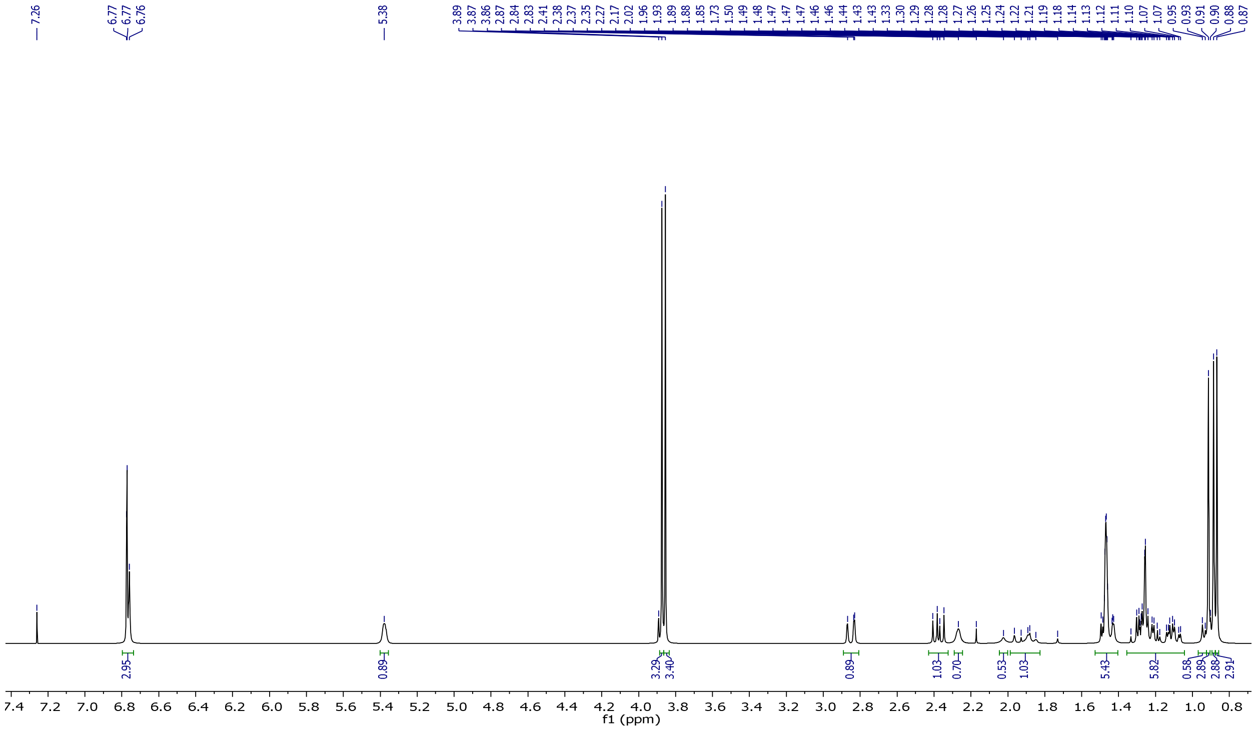

^{13}C NMR 11c

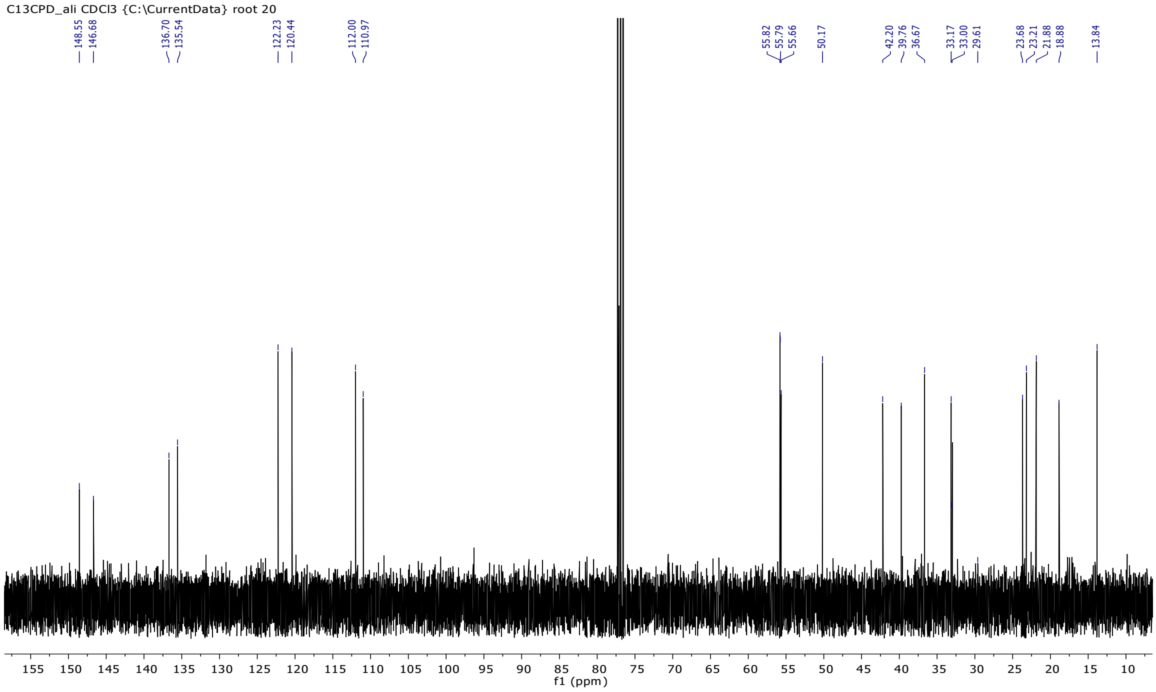

HRMS for 11c

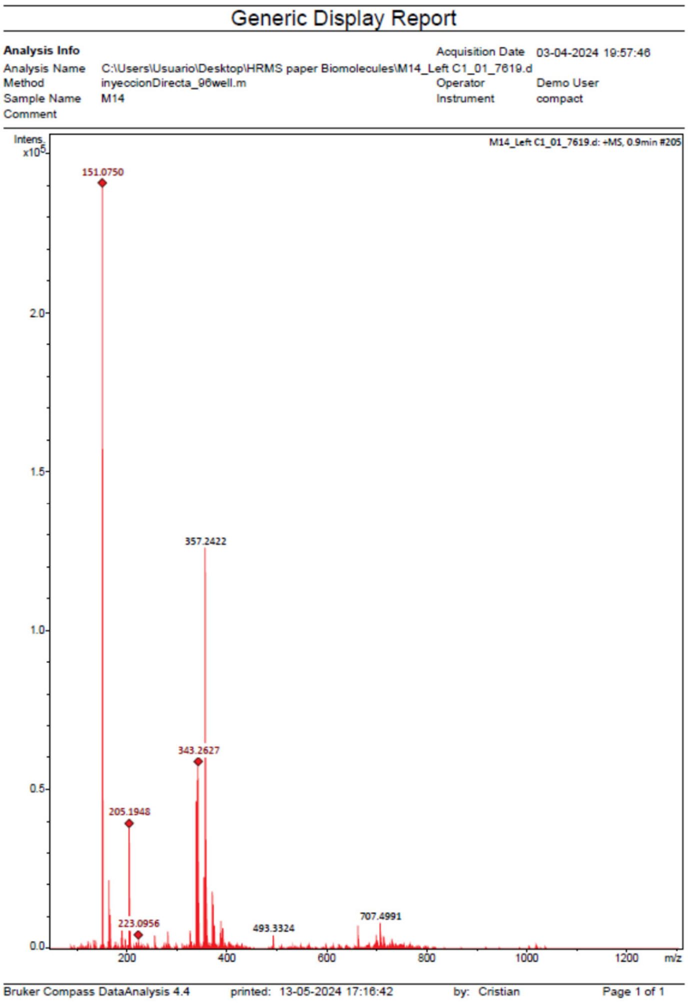

(4a*S*,5*S*)-5-(3,5-Dimethoxybenzyl)-1,1,4a,6-tetramethyl-1,2,3,4,4a,5,8,8a-octahydronaphthalene (**11d**)

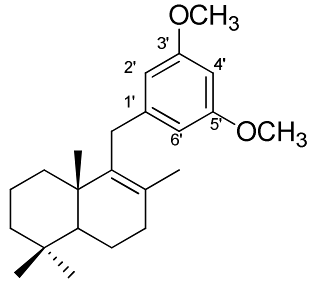

^{1}H NMR 11d

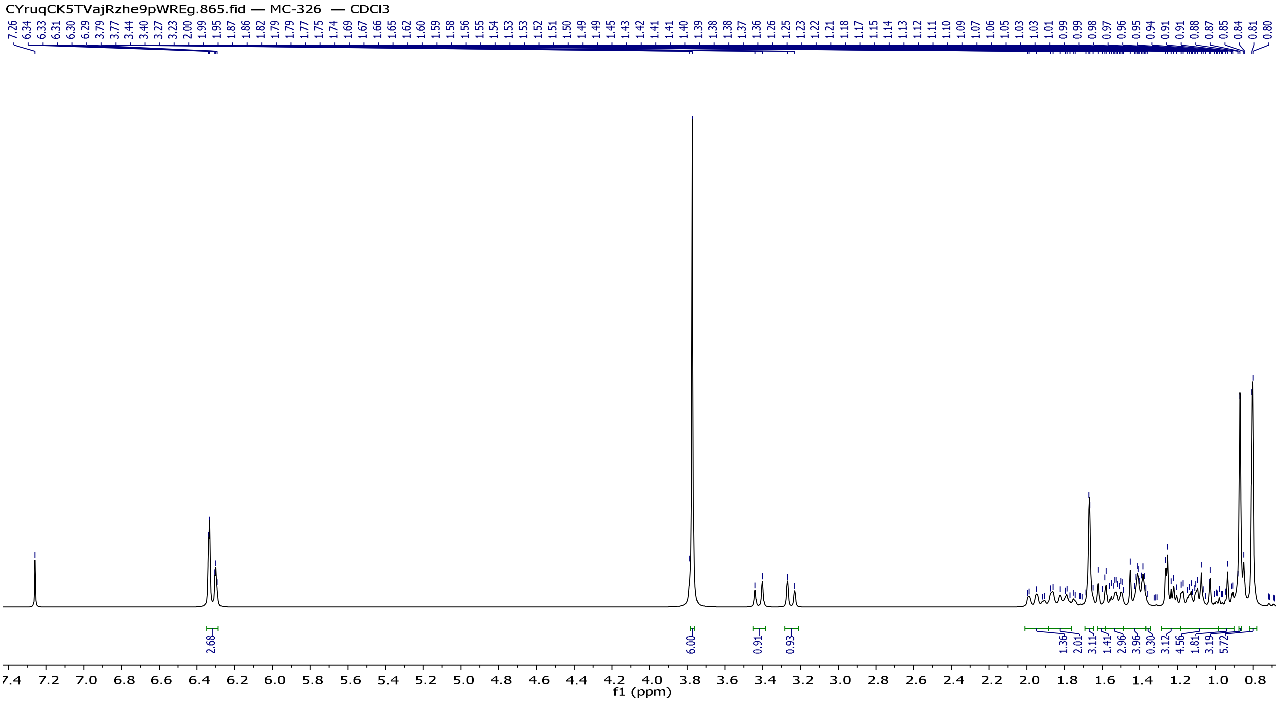

^{13}C NMR 11d

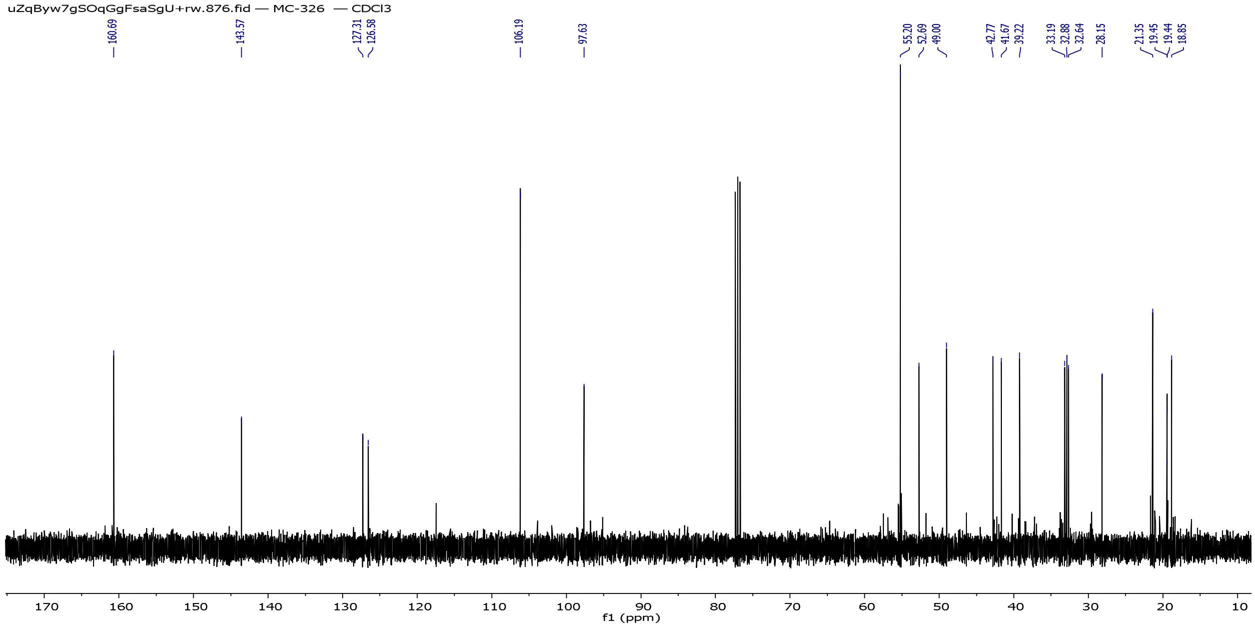
uZqByw7gSOqGgFsaSgU+rw.876.fid — MC-326 — CDCl3
160.69
143.57
127.31
126.58
106.19
97.63
55.20
52.69
49.00
42.77
41.67
39.22
33.19
32.88
32.64
28.15
21.35
19.45
19.44
18.85
170
160
150
140
130
120
110
100
90
80
70
60
50
40
30
20
10
f1 (ppm)

Ketone compounds 12a-d.

(2,4-Dimethoxyphenyl)((1*S*,8a*S*)-2,5,5,8a-tetramethyl-1,4,4a,5,6,7,8,8a-octahydronaphthalen-1-yl)methanone (**12a**)

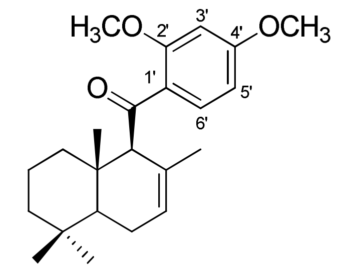

^{1}H NMR 12a

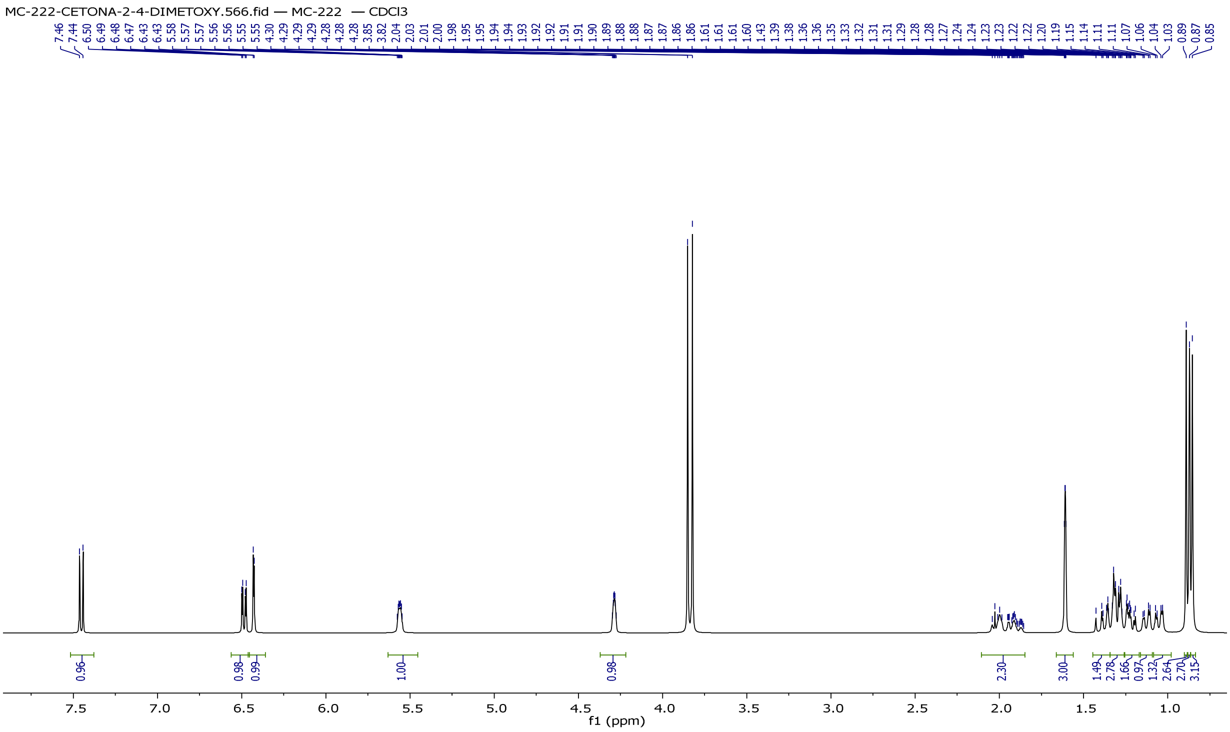

^{13}C NMR 12a

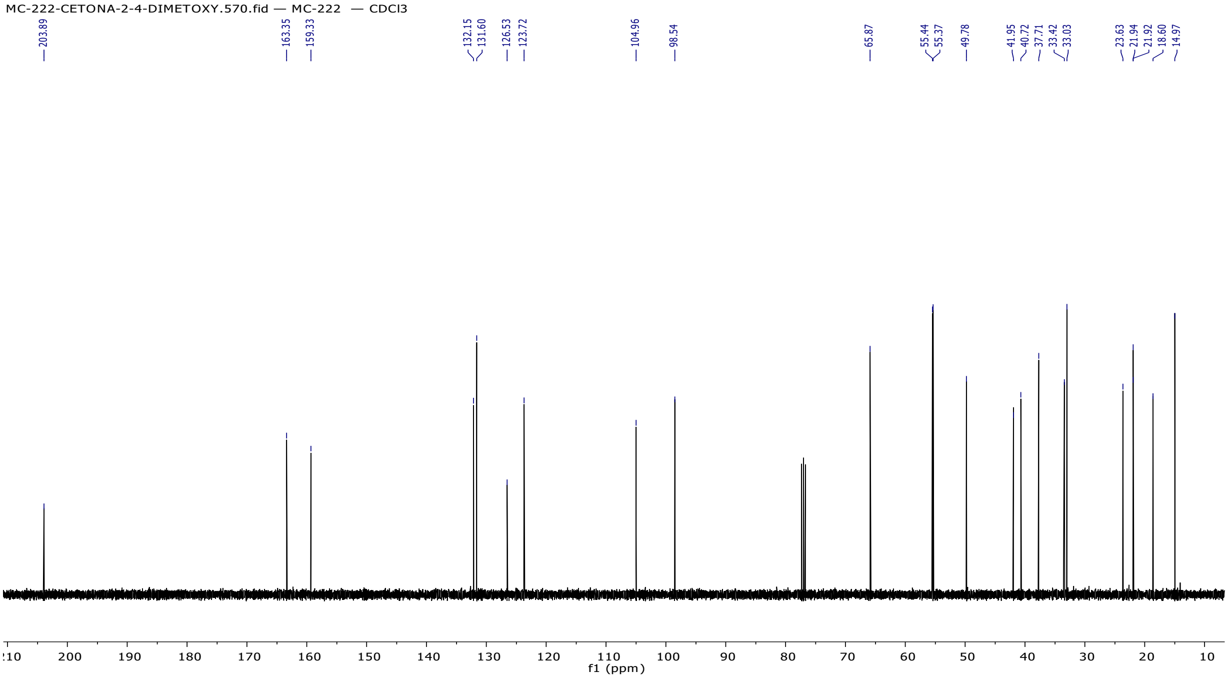

HRMS for 12a

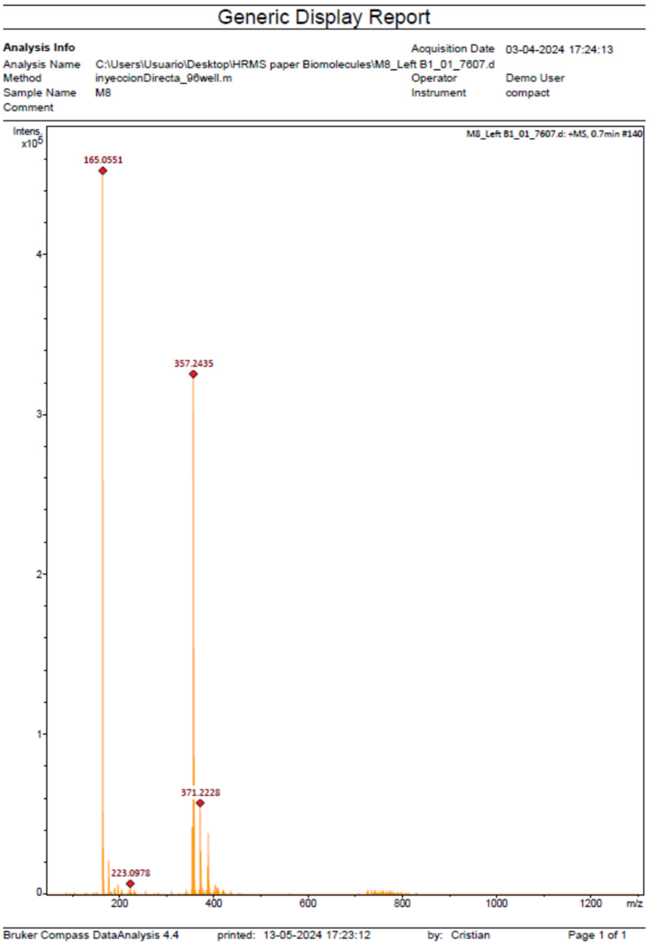

(2,5-Dimethoxyphenyl)((1*S*,8a*S*)-2,5,5,8a-tetramethyl-1,4,4a,5,6,7,8,8a-octahydronaphthalen-1-yl)methanone (**12b**)

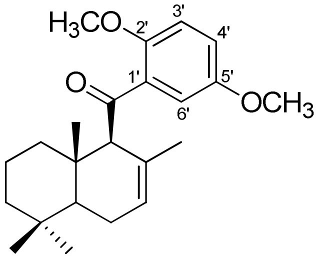

^{1}H NMR 12b

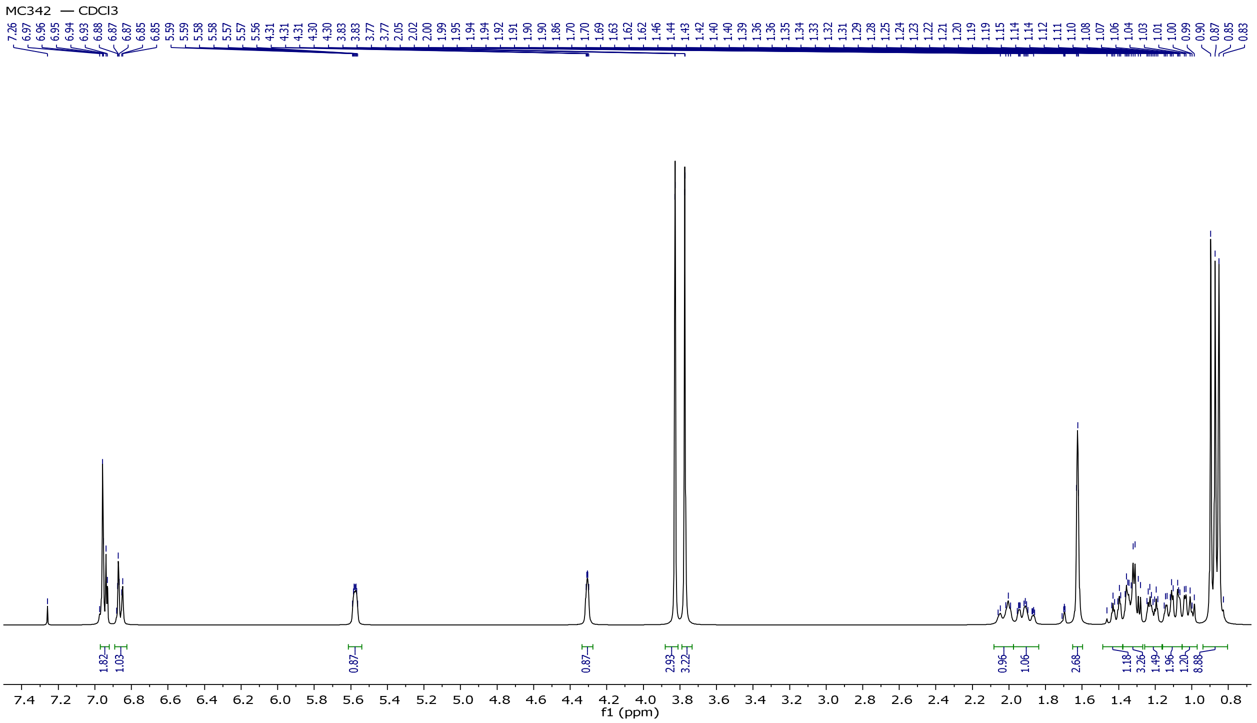

^{13}C NMR 12b

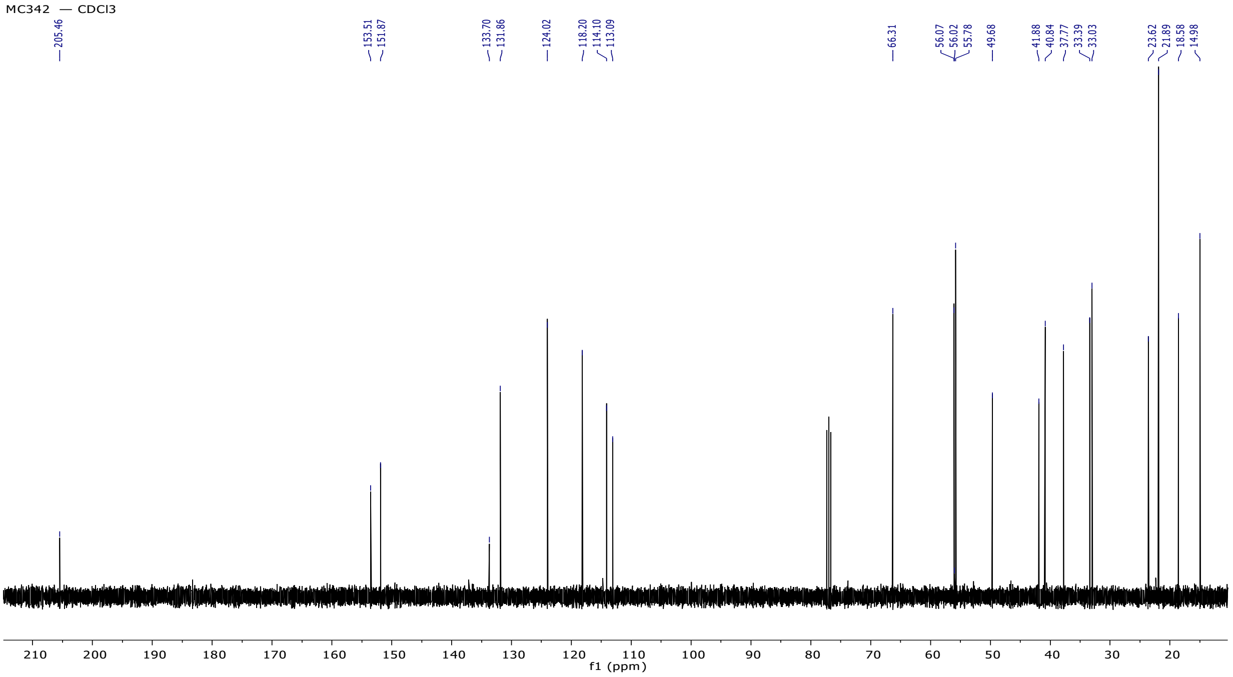

HRMS for 12b

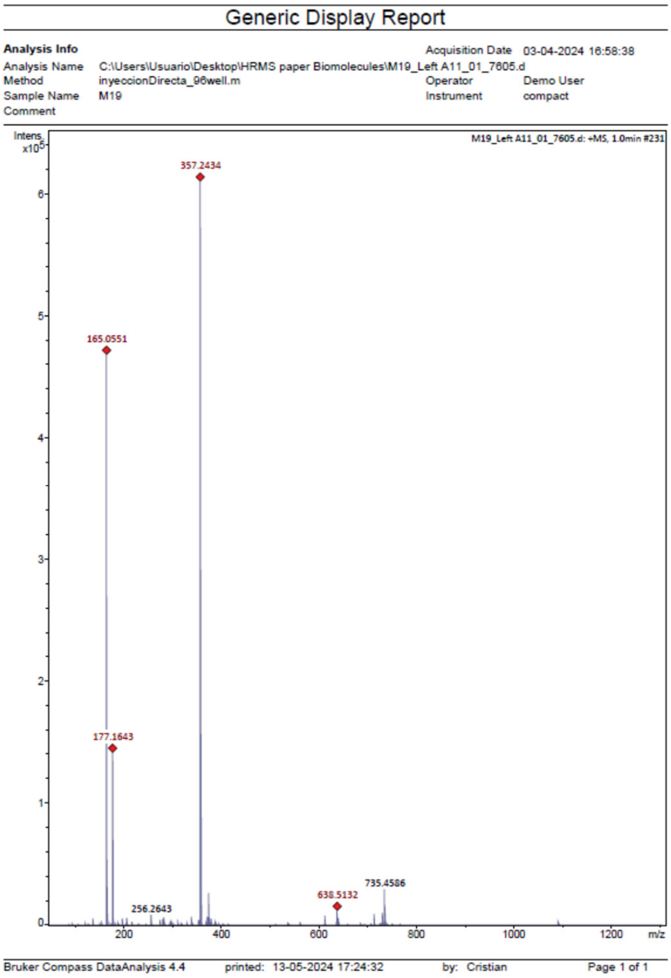

(3,4-Dimethoxyphenyl)((1*S*,8a*S*)-2,5,5,8a-tetramethyl-1,4,4a,5,6,7,8,8a-octahydronaphthalen-1-yl)methanone (**12c**)

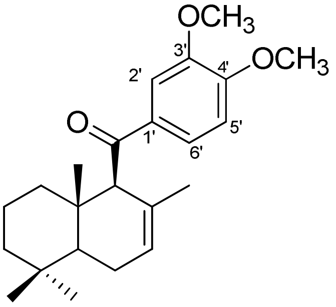

^{1}H NMR 12c

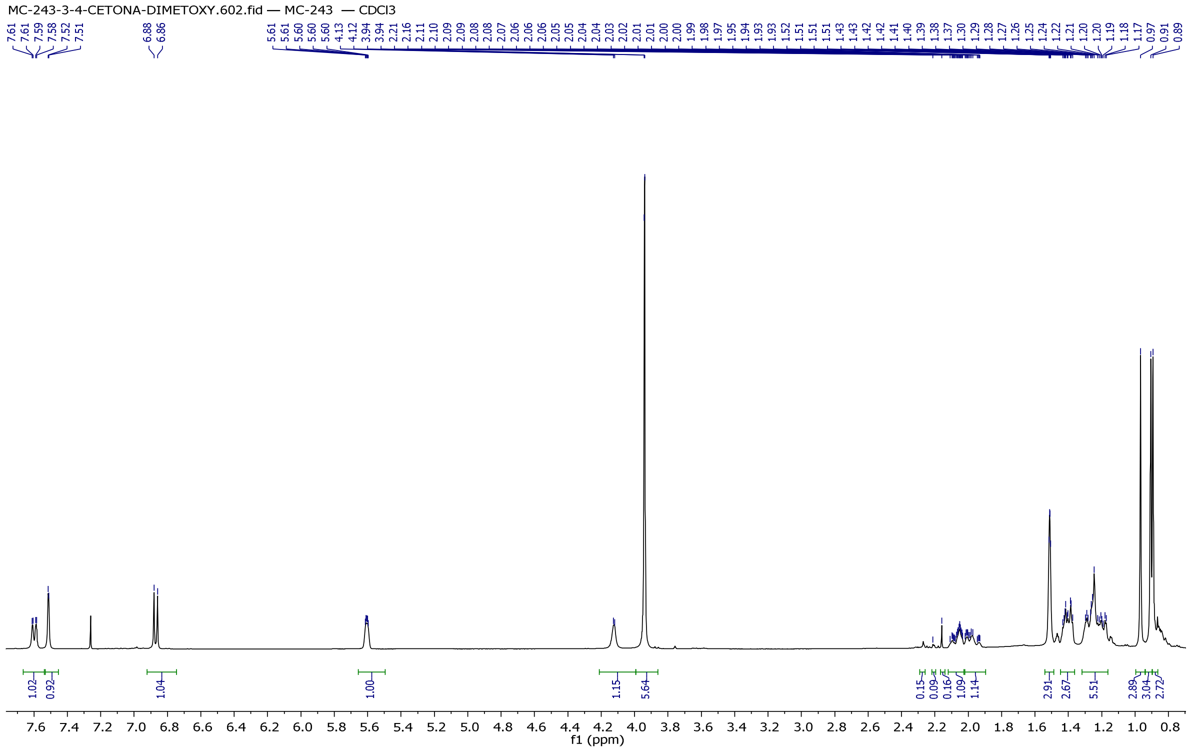

^{13}C NMR 12c

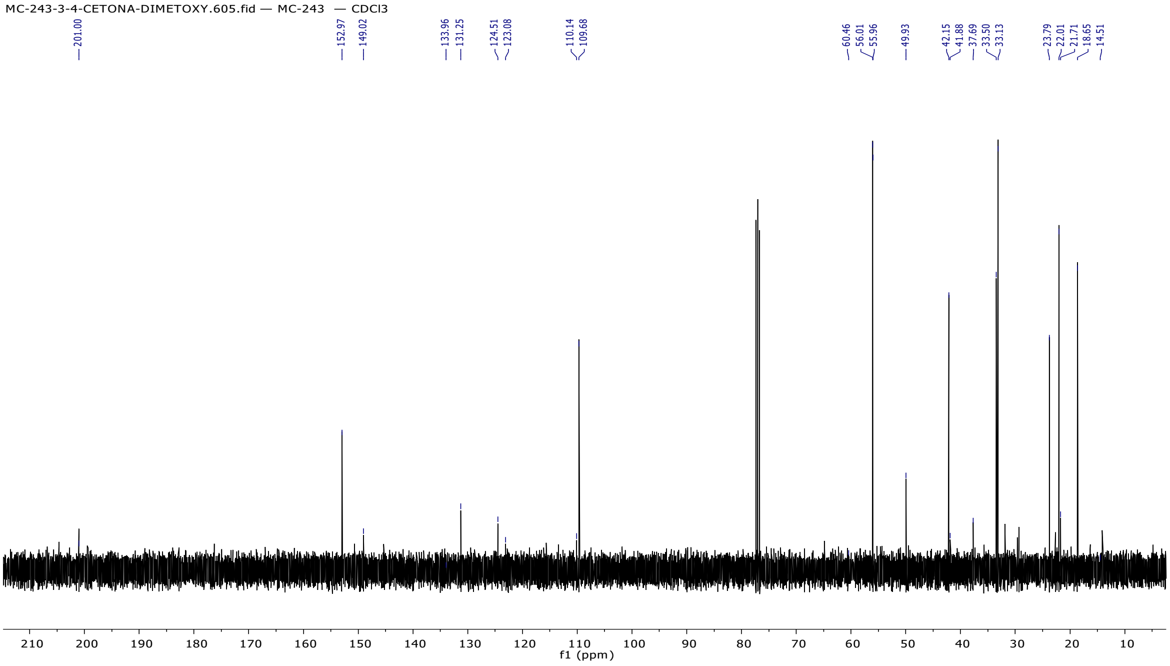

HRMS for 12c

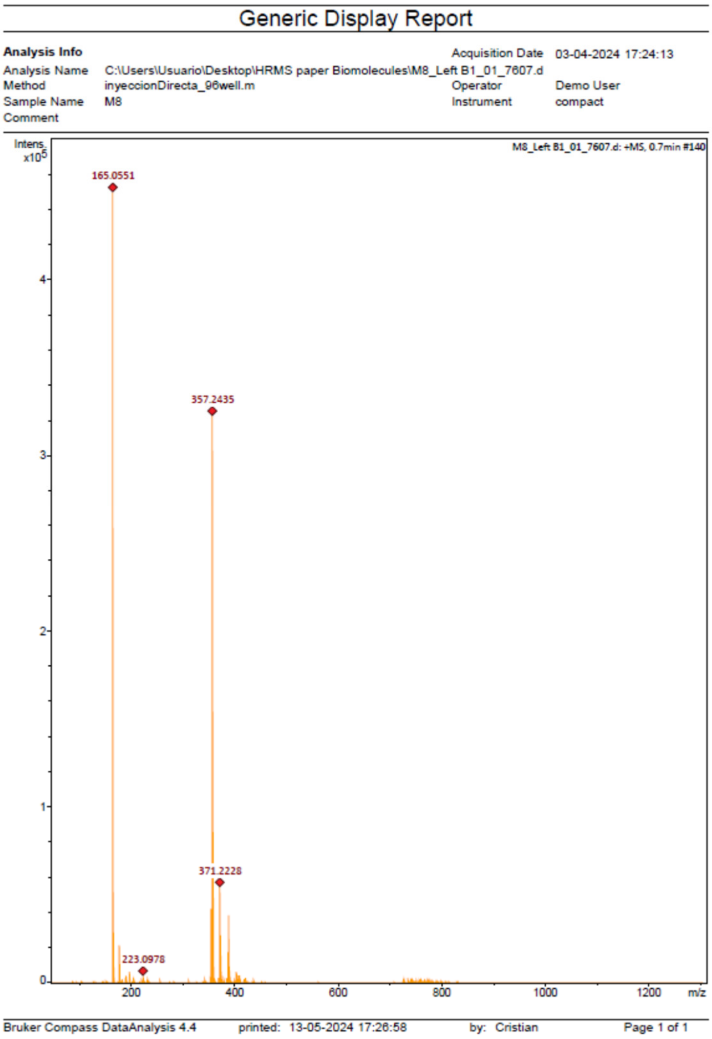

(3,5-Dimethoxyphenyl)((1*S*,8a*S*)-2,5,5,8a-tetramethyl-1,4,4a,5,6,7,8,8a-octahydronaphthalen-1-yl)methanone (**12d**)

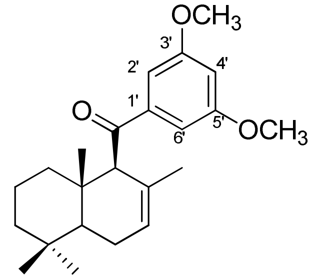

^{1}H NMR 12d

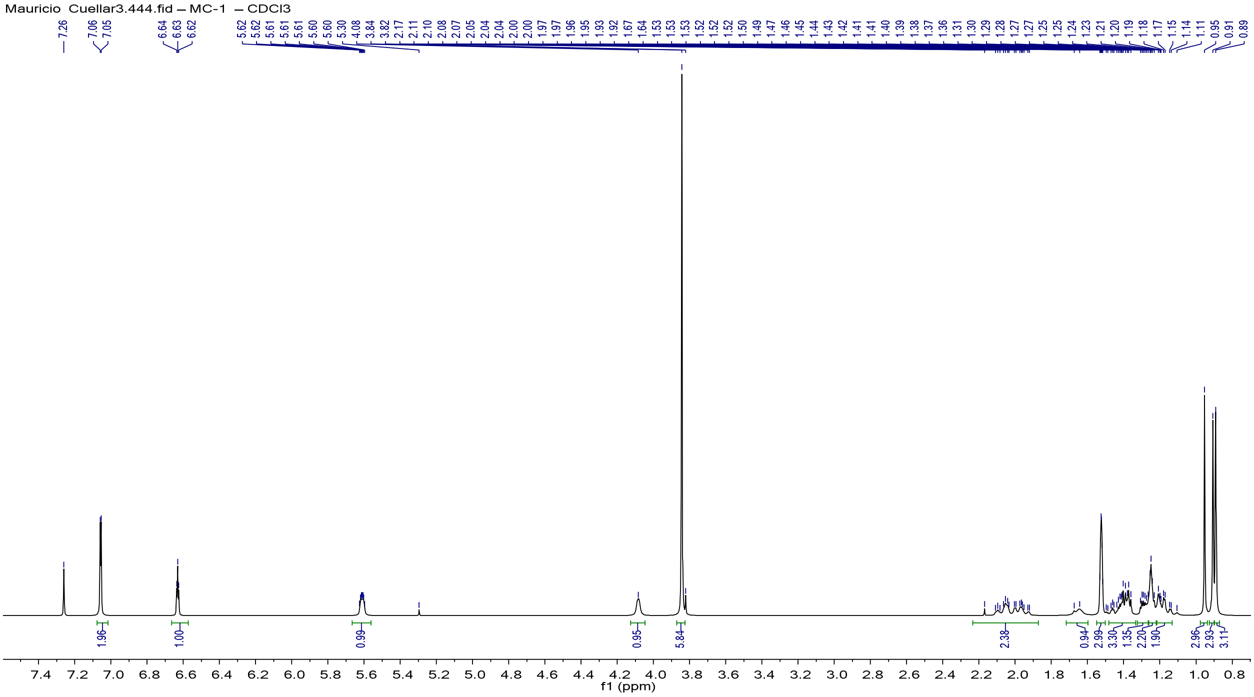

^{13}C NMR 12d

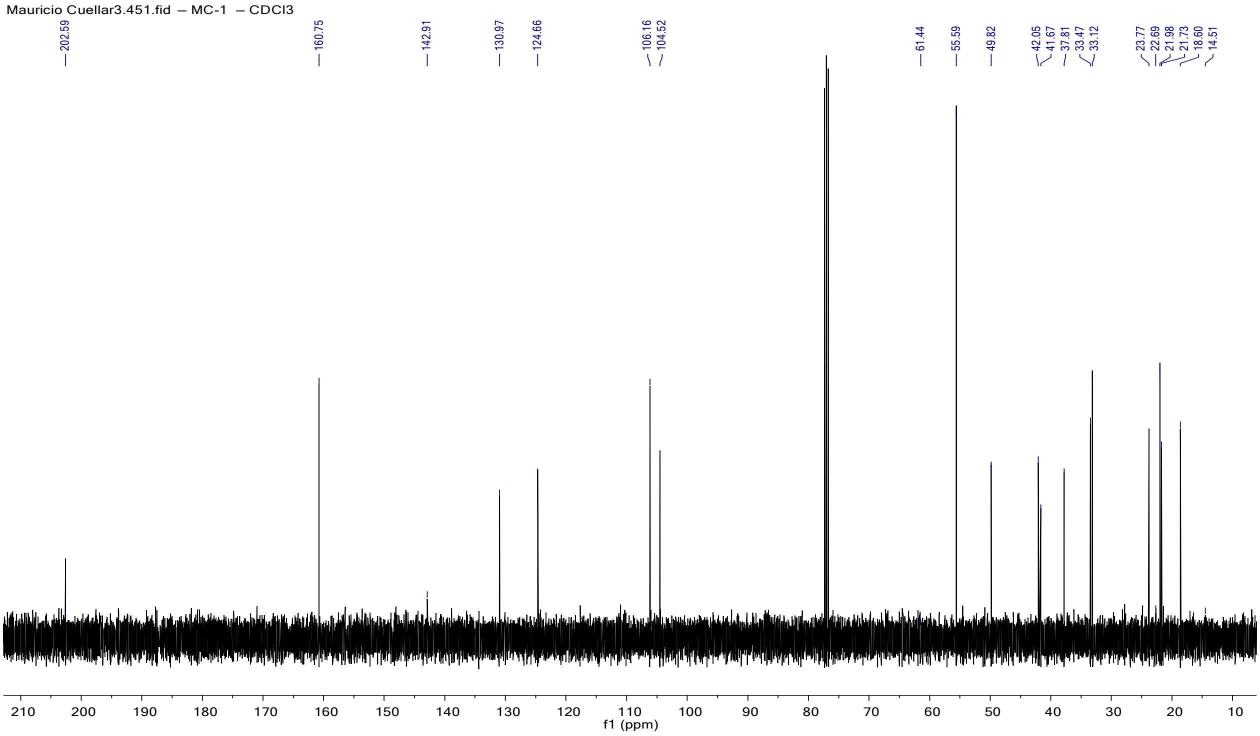

HRMS for 12d

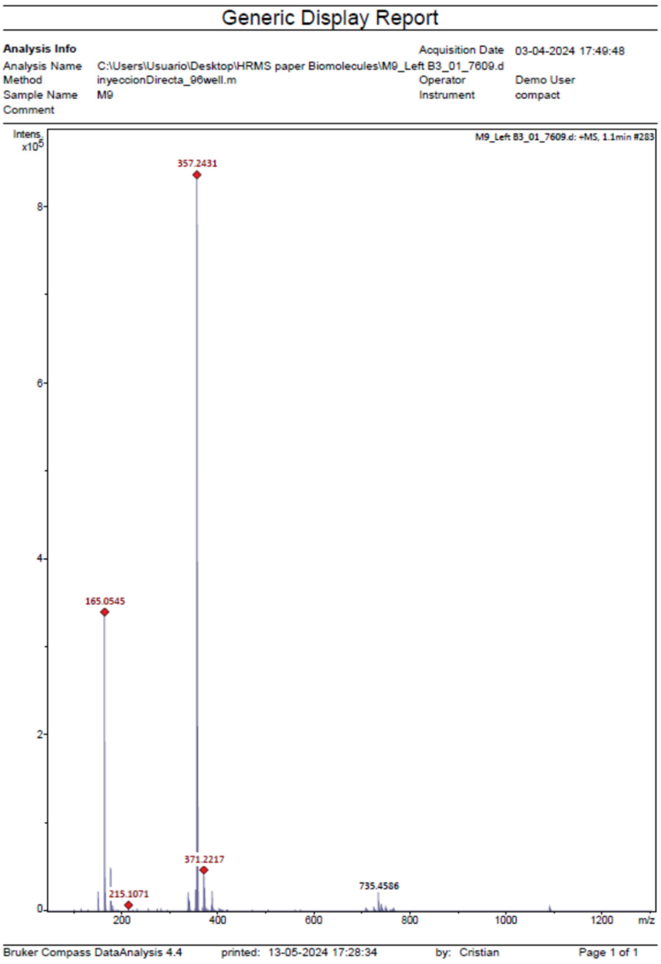

Generic Display Report

Analysis Info		Acquisition Date	03-04-2024 17:49:48
Analysis Name	C:\Users\Usuario\Desktop\HRMS paper Biomolecules\M9_Left B3_01_7609.d		
Method	inyeccionDirecta_96well.m	Operator	Demo User
Sample Name	M9	Instrument	compact
Comment			

Bruker Compass DataAnalysis 4.4 printed: 13-05-2024 17:28:34 by: Cristian Page 1 of 1

Diene compounds 13a and 13c.

(4a*S*)-5-((*E*)-2,4-Dimethoxybenzylidene)-1,1,4a,6-tetramethyl-1,2,3,4,4a,5,8,8a-octahydronaphthalene (**13a**)

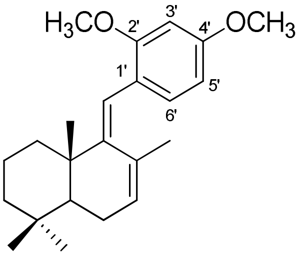

^{1}H NMR 13a

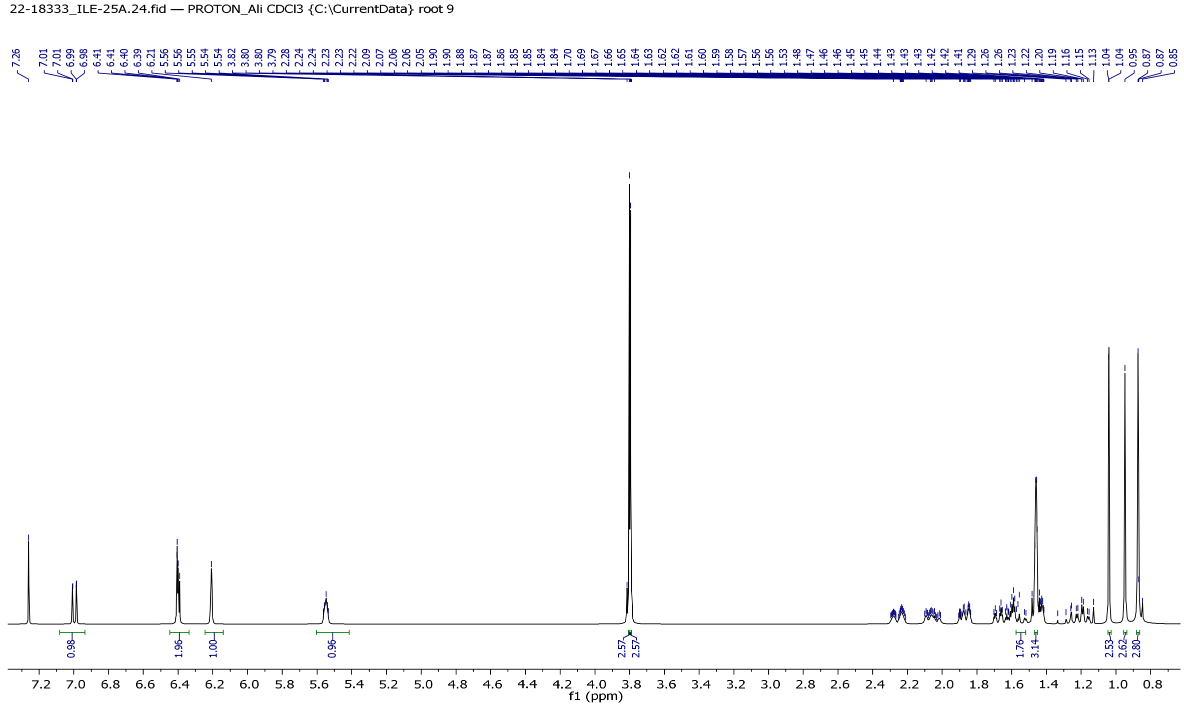

^{13}C NMR 13a

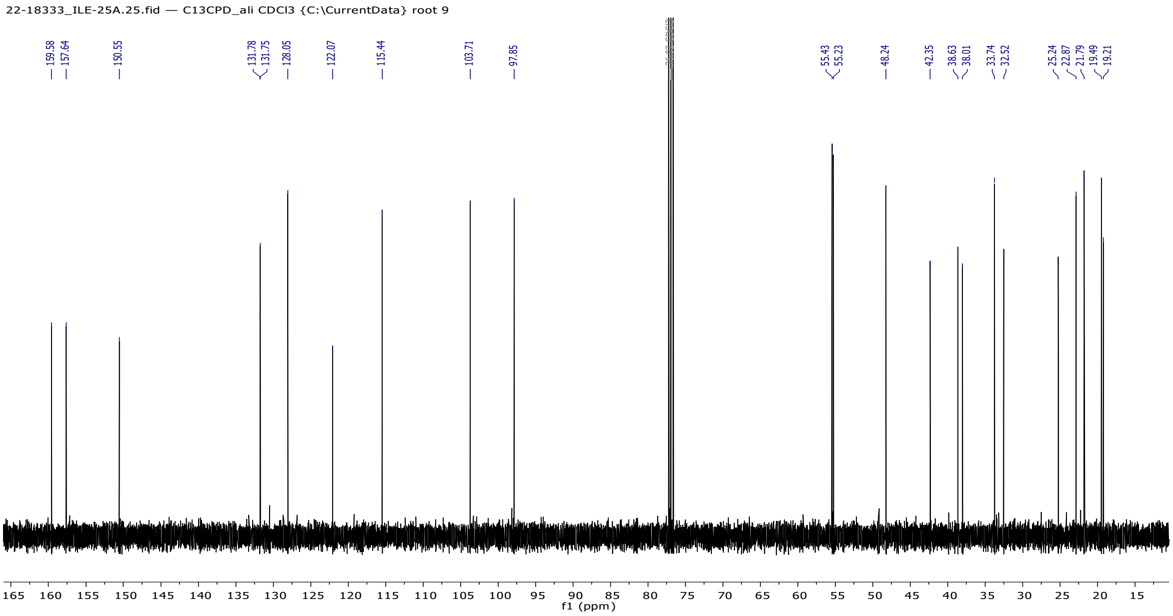
22-18333_ILE-25A.25.fid — C13CPD_ali CDCl3 {C:\CurrentData} root 9
159.58
157.64
150.55
131.78
131.75
128.05
122.07
115.44
103.71
97.85
55.43
55.23
48.24
42.35
38.63
38.01
33.74
32.52
25.24
22.87
21.79
19.49
19.21
f1 (ppm)

HRMS for 13a

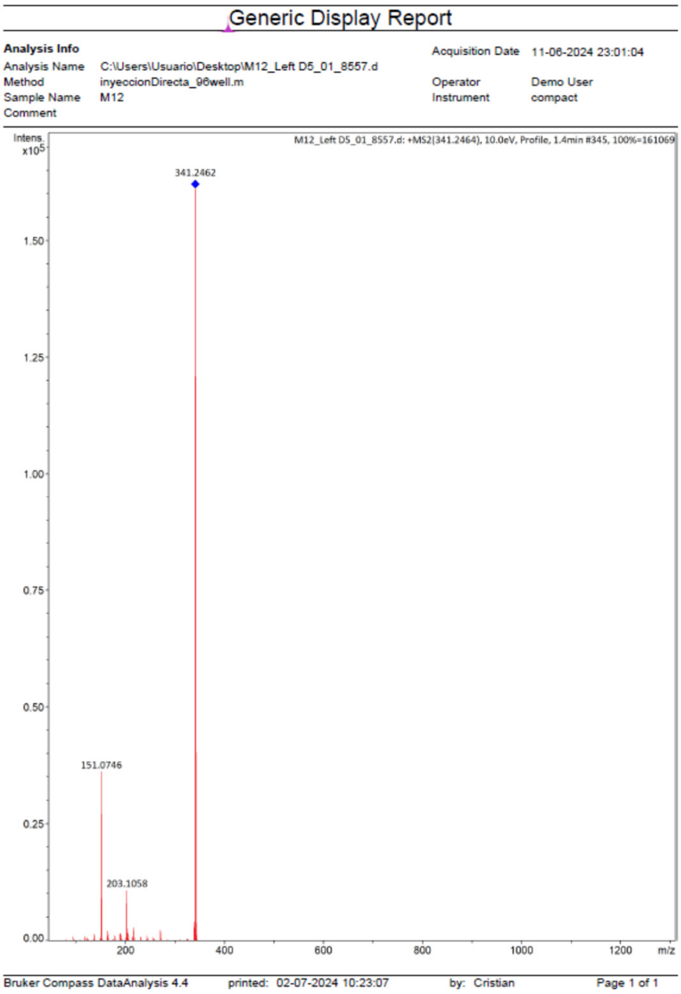
Generic Display Report
Analysis Info
Acquisition Date 11-06-2024 23:01:04
Analysis Name C:\Users\Usuario\Desktop\M12_Left D5_01_8557.d
Method inyeccionDirecta_96well.m
Operator Demo User
Sample Name M12
Instrument compact
Comment
Intens. x10^5
M12_Left D5_01_8557.d: +MS2(341.2464), 10.0eV, Profile, 1.4min #345, 100%=161069
341.2462
151.0746
203.1058
m/z
Bruker Compass DataAnalysis 4.4
printed: 02-07-2024 10:23:07
by: Cristian
Page 1 of 1

(4a*S*)-5-((*E*)-3,4-Dimethoxybenzylidene)-1,1,4a,6-tetramethyl-1,2,3,4,4a,5,8,8a-octahydronaphthalene (**13c**)

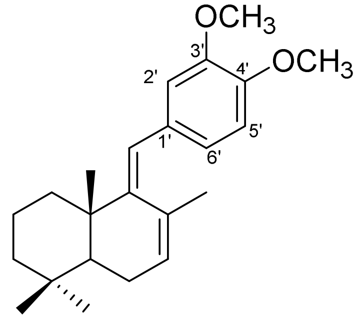

^{1}H NMR 13c

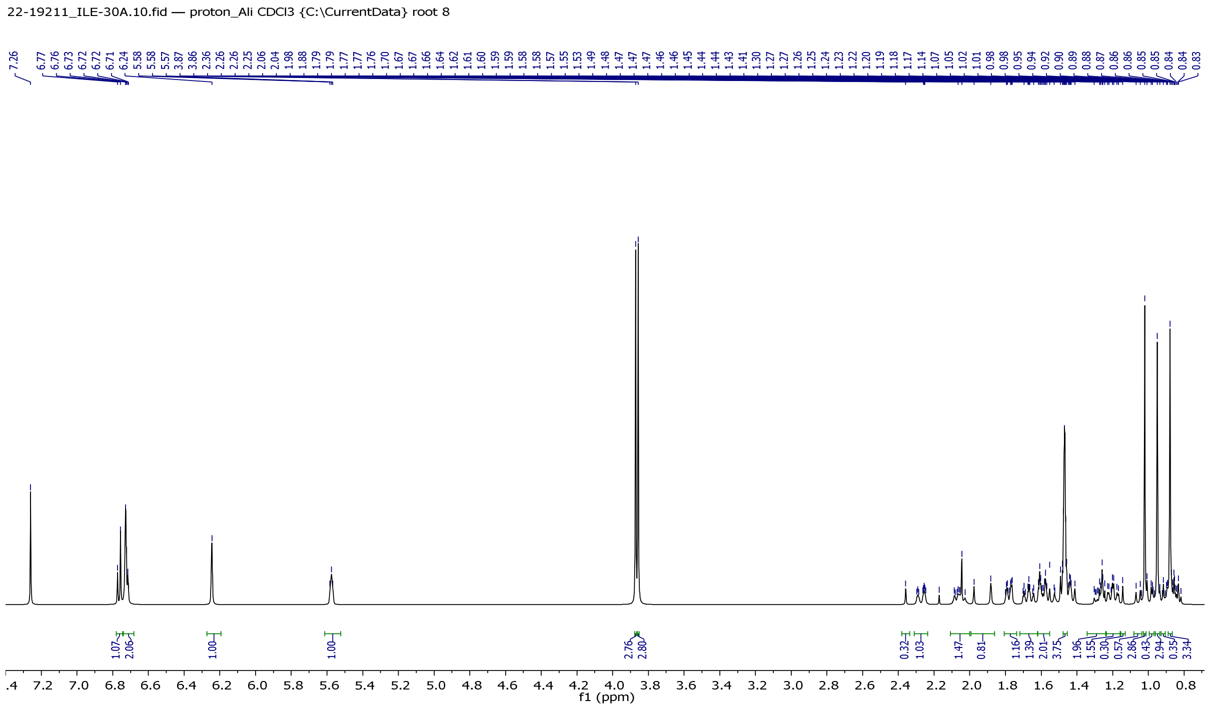

^{13}C NMR 13c

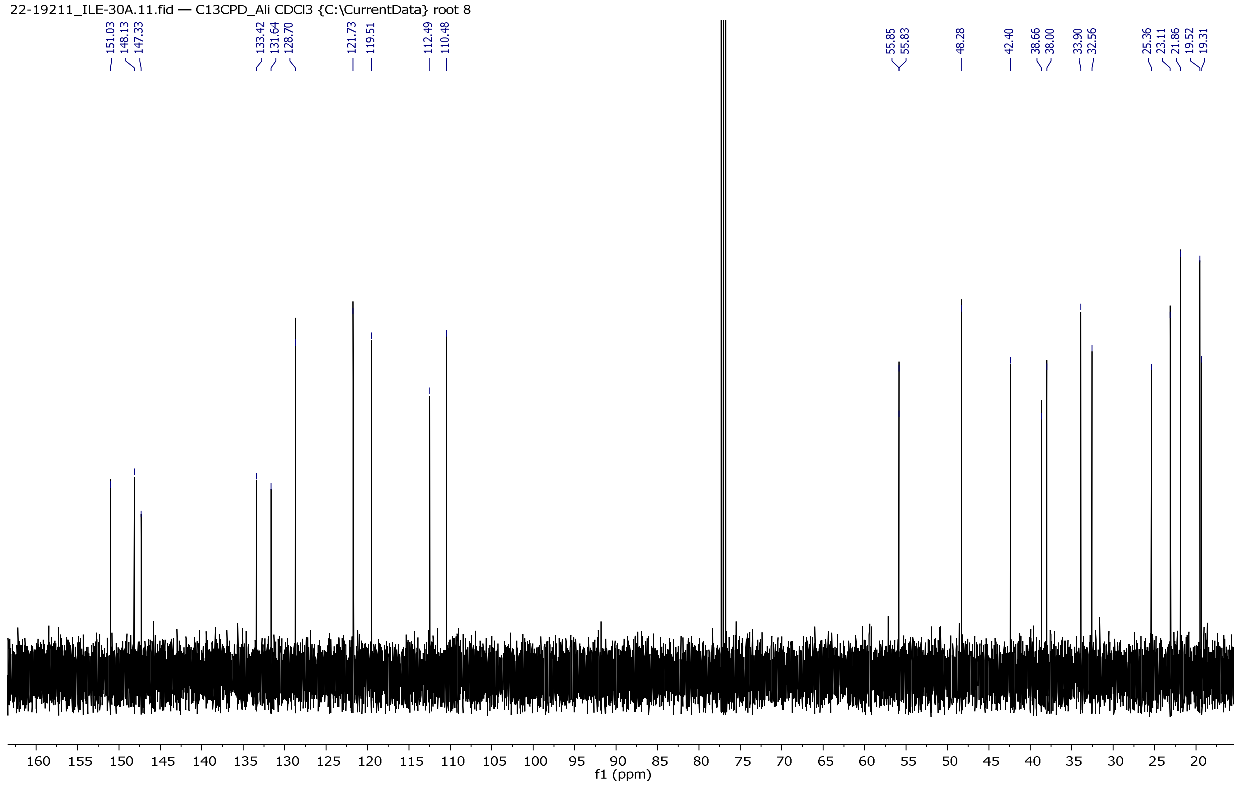

HRMS for 13c

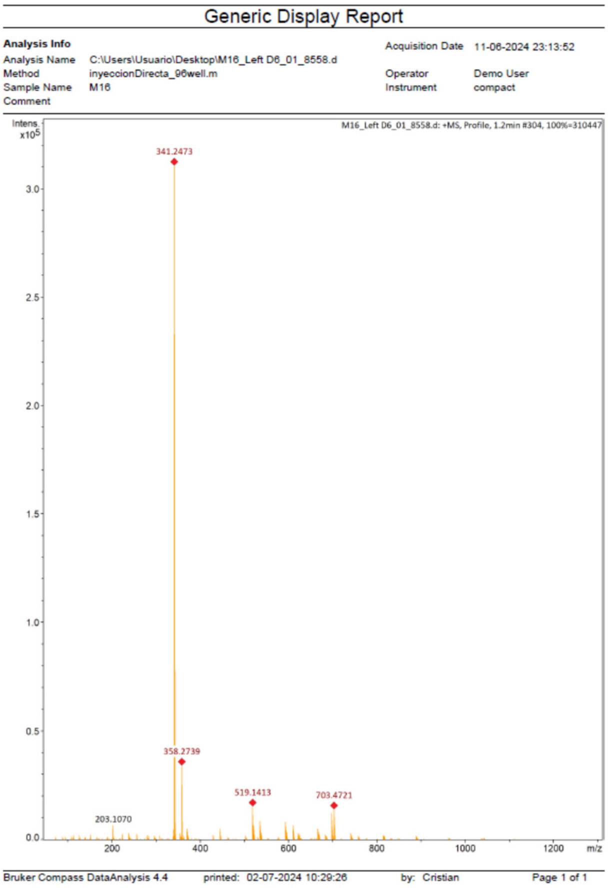

Cyclic compounds 14a, 14c, and 14d.

(6a*R*,11b*S*)-8,10-Dimethoxy-4,4,6a,11b-tetramethyl-2,3,4,4a,5,6,6a,11b-octahydro-1H-benzo[a]fluorene (**14a**)

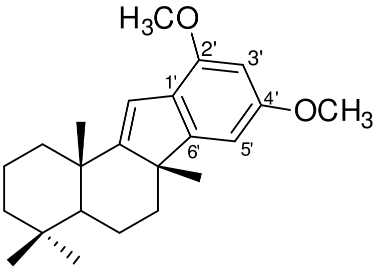

^{1}H NMR 14a

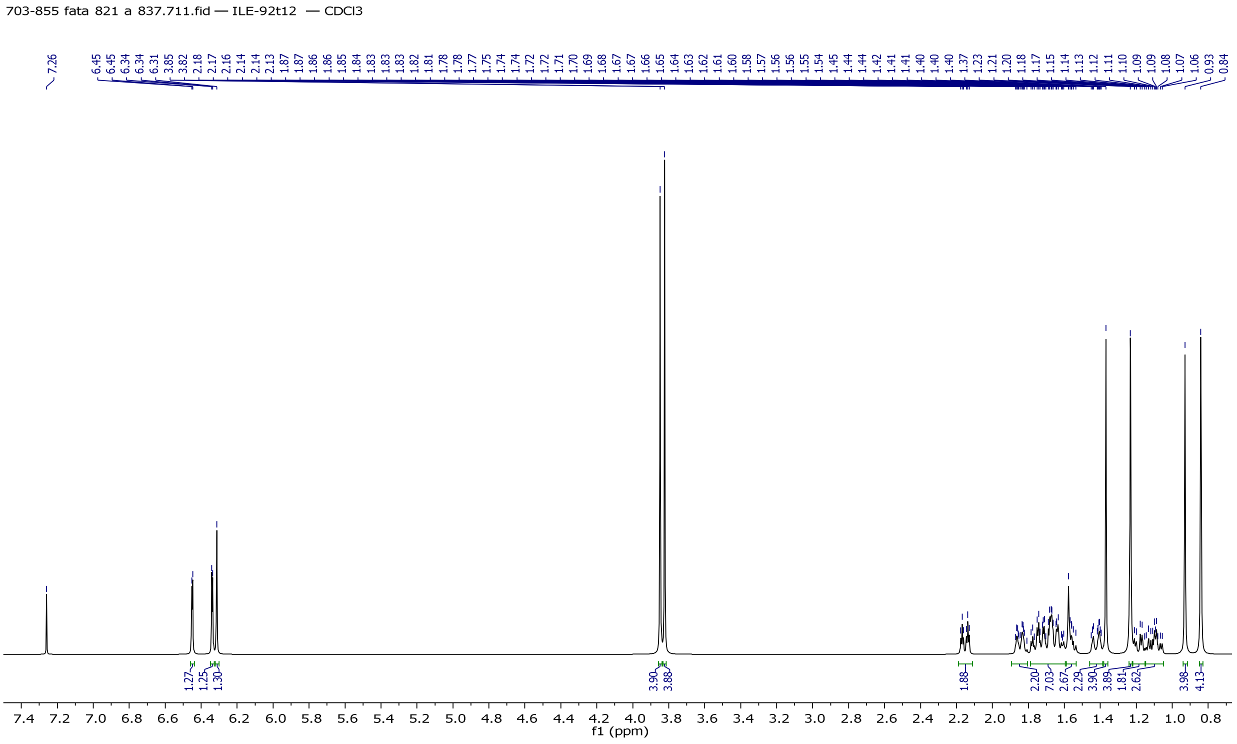

^{13}C NMR 14a

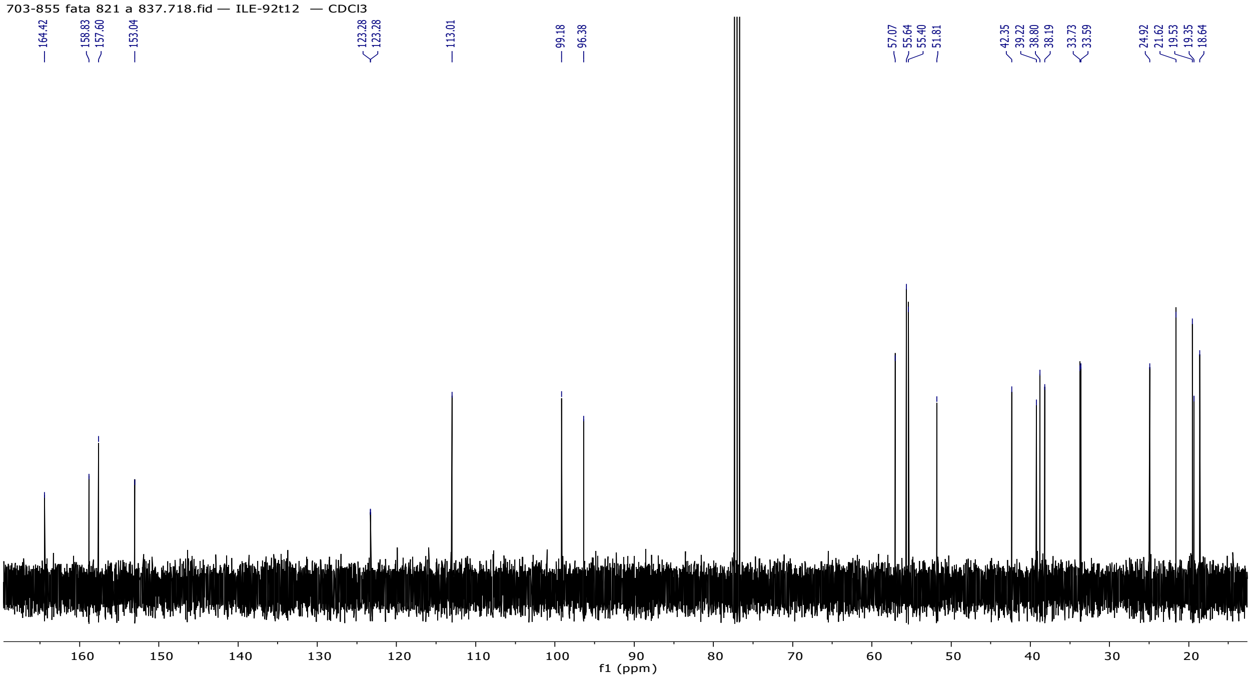
703-855 fata 821 a 837.718.fid — ILE-92t12 — CDCl3
164.42
158.83
157.60
153.04
123.28
123.28
113.01
99.18
96.38
57.07
55.64
55.40
51.81
42.35
39.22
38.80
38.19
33.73
33.59
24.92
21.62
19.53
19.35
18.64
160 150 140 130 120 110 100 90 80 70 60 50 40 30 20
f1 (ppm)

HRMS for 14a

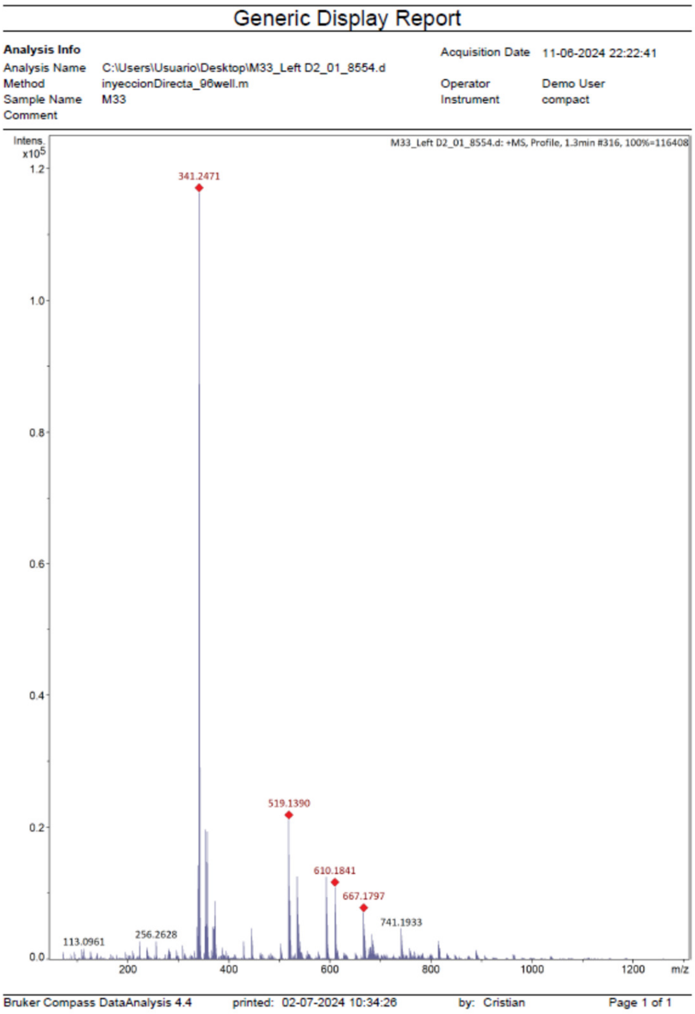
Generic Display Report
Analysis Info
Analysis Name C:\Users\Usuario\Desktop\M33_Left D2_01_8554.d
Method inyeccionDirecta_96well.m
Sample Name M33
Comment
Acquisition Date 11-06-2024 22:22:41
Operator Demo User
Instrument compact
M33_Left D2_01_8554.d: +MS, Profile, 1.3min #316, 100%=116408
Intens.
x10^5
341.2471
519.1390
610.1841
667.1797
741.1933
256.2628
113.0961
m/z
Bruker Compass DataAnalysis 4.4
printed: 02-07-2024 10:34:26
by: Cristian
Page 1 of 1

(6a*R*,11b*S*)-8,9-Dimethoxy-4,4,6a,11b-tetramethyl-2,3,4,4a,5,6,6a,11b-octahydro-1H-benzo[a]fluorene (**14c**)

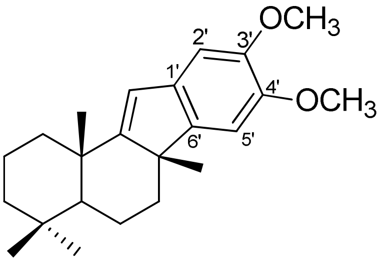

^{1}H NMR 14c

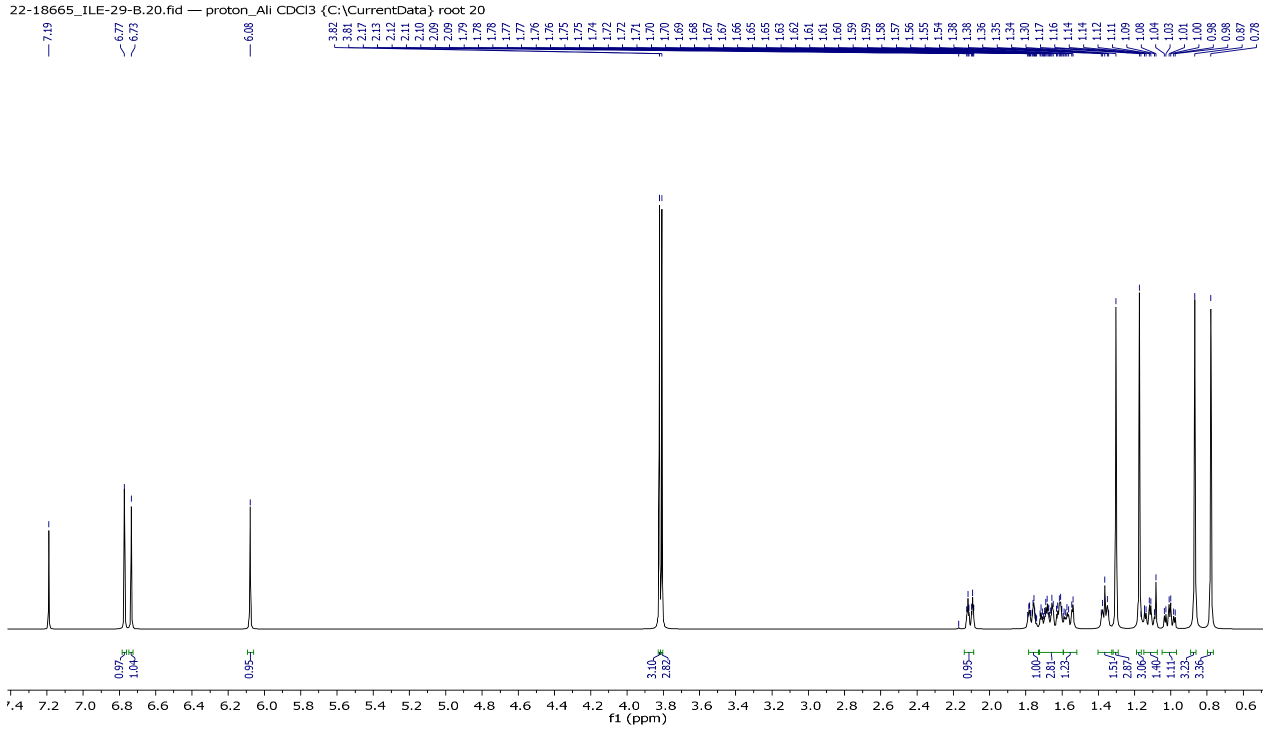

^{13}C NMR 14c

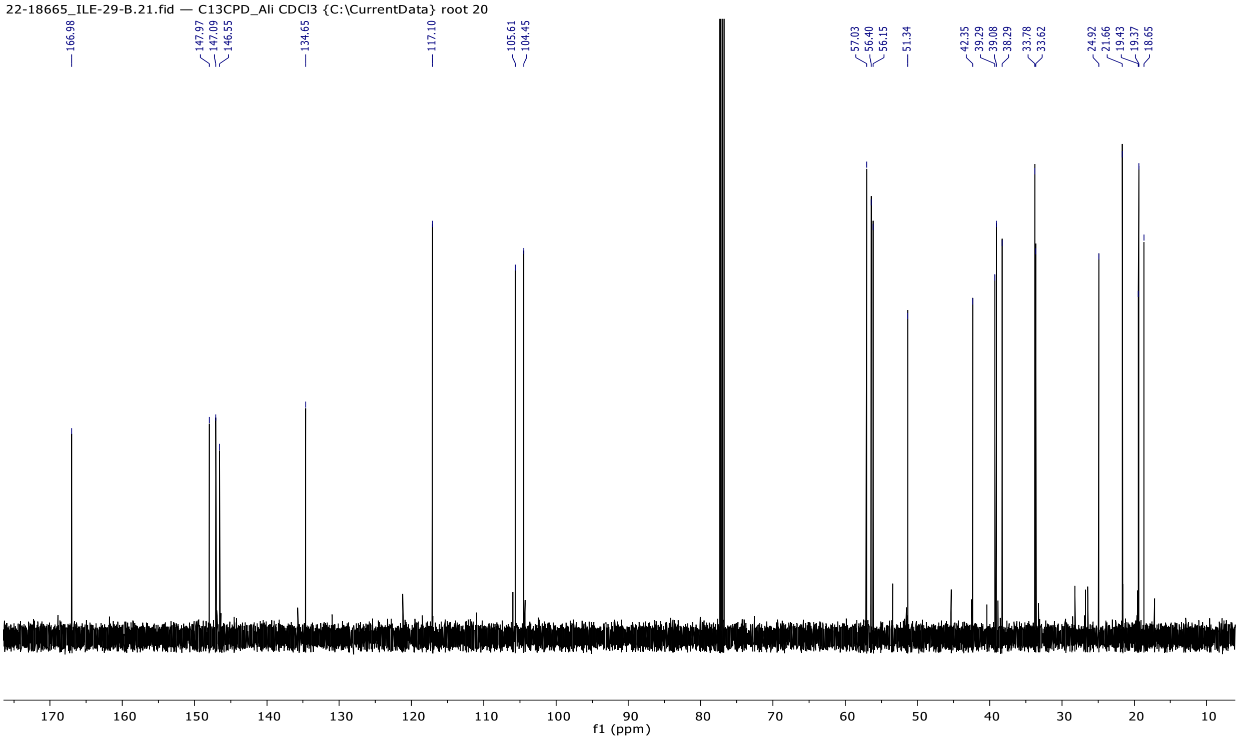

HRMS for 14c

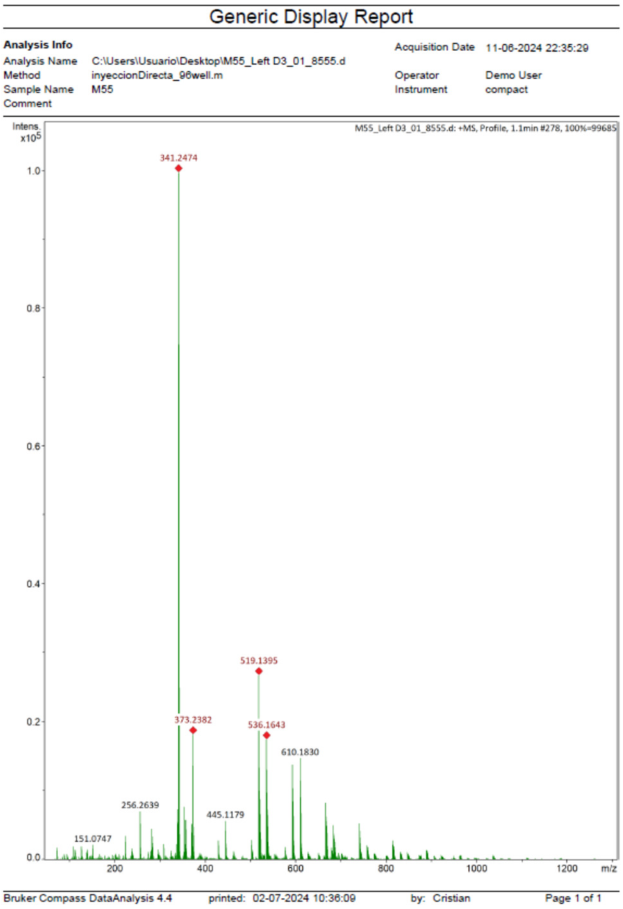

(6a*R*,11b*S*)-7,9-Dimethoxy-4,4,6a,11b-tetramethyl-2,3,4,4a,5,6,6a,11b-octahydro-1H-benzo[a]fluorene (**14d**)

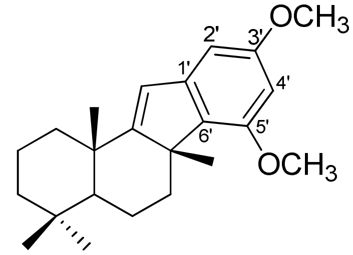

^{1}H NMR 14d

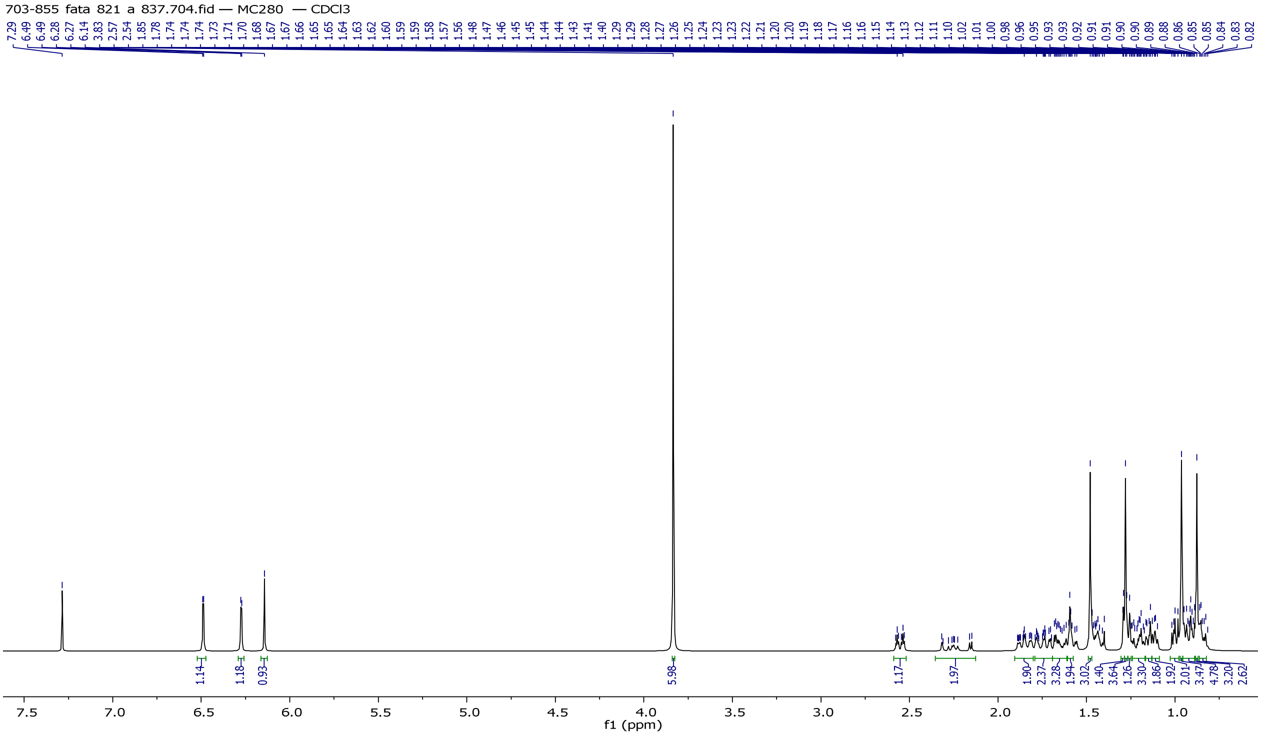

^{13}C NMR 14d

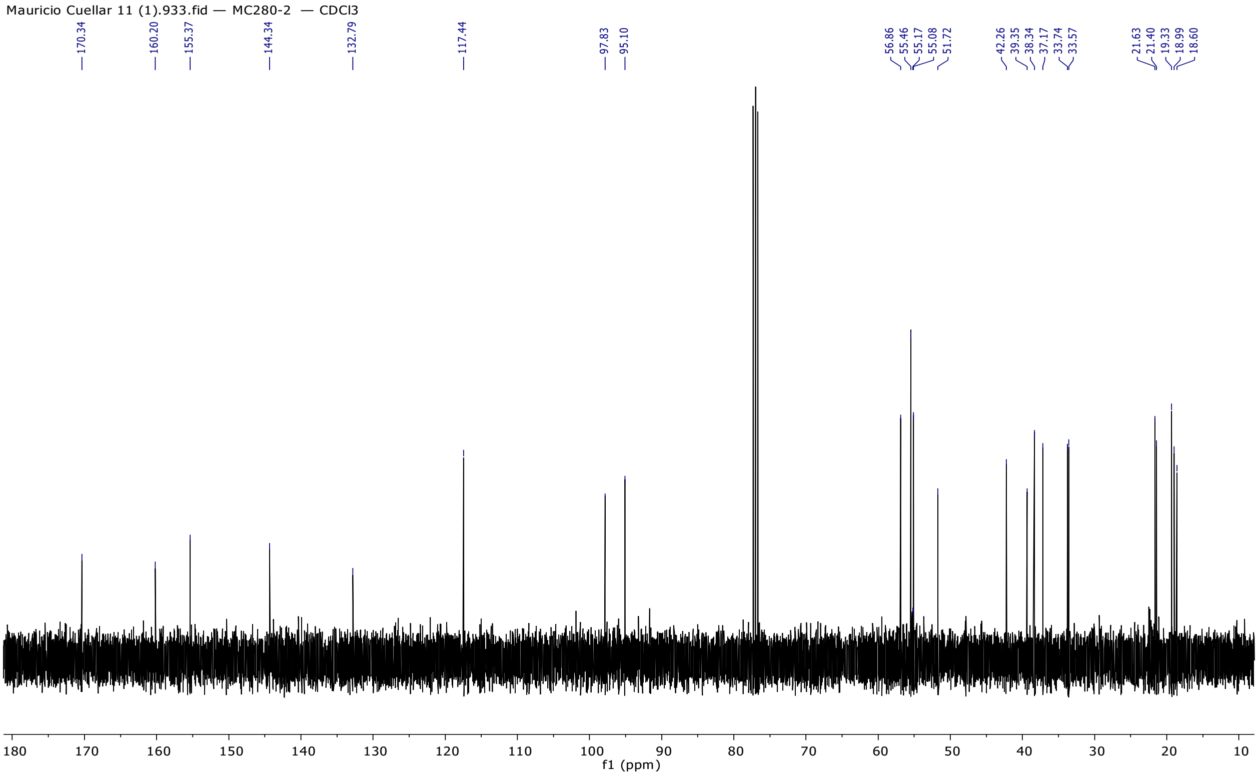

HRMS for 14d

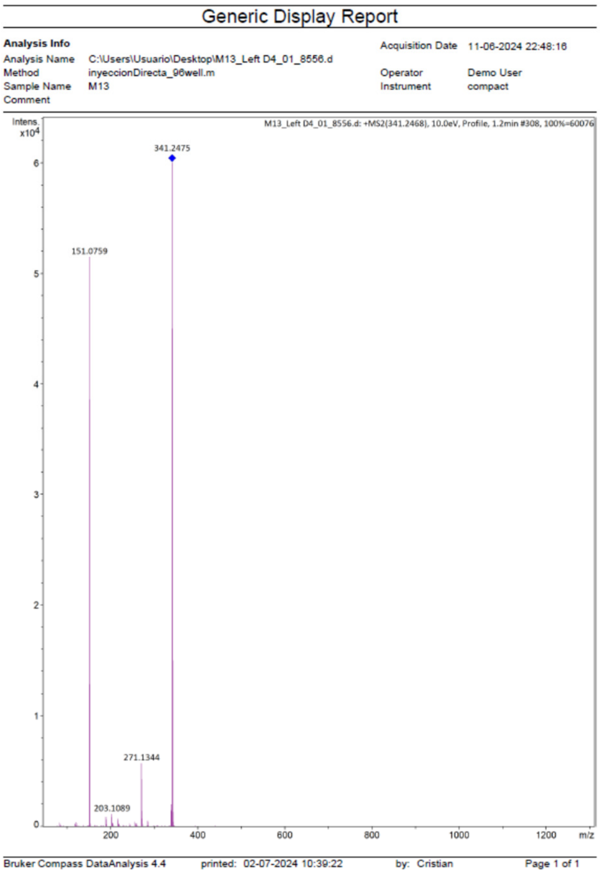

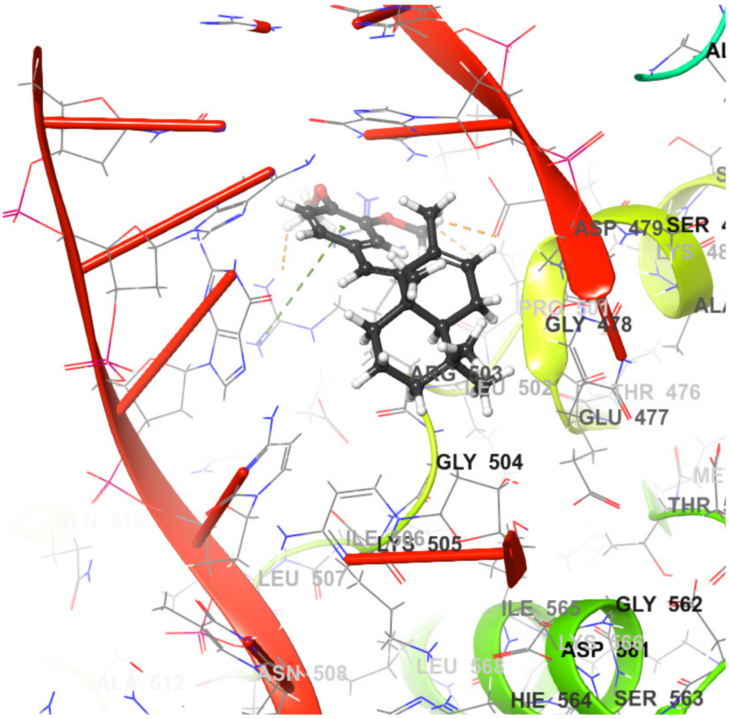

Figure S1.1. Best docking pose for compound **13c** in the DNA-Topoisomerase II complex (PDB ID: 3QX3). XP GScore = -5.321 kcal/mol. In orange dotted lines: bad contact clashes. In green dotted line cation-π interaction between benzene ring and Arg503.

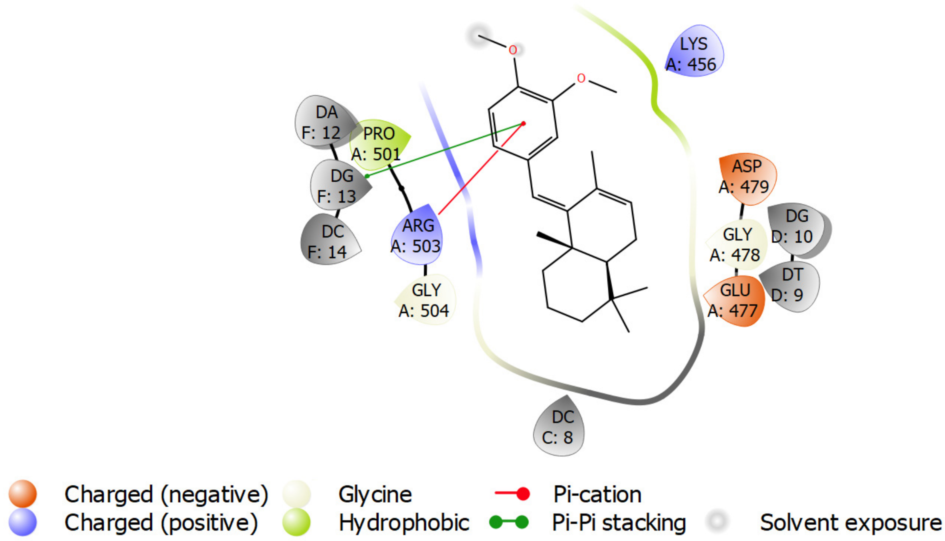

Figure S1.2. 2D interaction diagram for compound **13c** in the DNA-Topoisomerase II complex (PDB ID: 3QX3). The 4Å distance environment from the ligand is depicted. The interaction with DG13 correspond to a π-T.

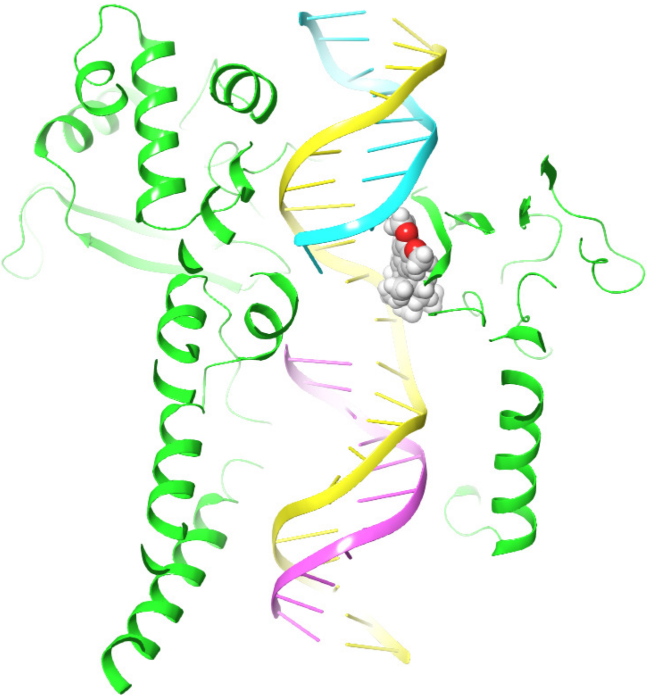

Figure S2.3. Far view of docking pose for compound **14c** in the DNA-Topoisomerase I complex (PDB ID: 1K4T). The compound does not achieve an intercalating accommodation in the DNA structure. No significative XP GScore was reported for this pose.

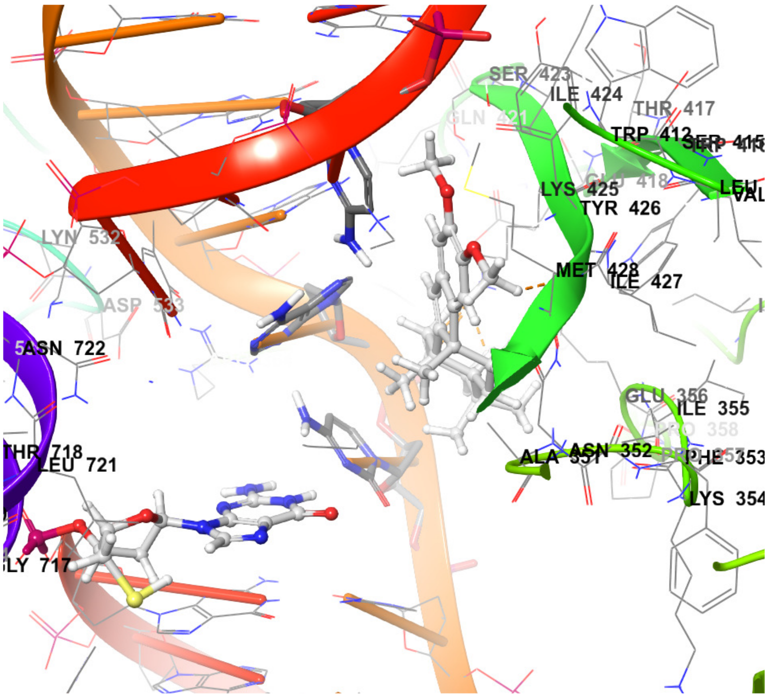

Figure S2.4. Close view of the docking pose for compound **14c** in the DNA-Topoisomerase I complex (PDB ID: 1K4T). In orange dotted lines bad contact clashes. No other significative interactions were obtained.
